# Programmable Chaotic Suspension Electrolysis for Scalable Manufacturing of Vacancy‐Tunable Electrolytic MnO_2_

**DOI:** 10.1002/advs.76766

**Published:** 2026-07-23

**Authors:** Zhihao Wu, Jie Yang, Yidan Fan, Wentao Li, Jiaxing Li, Jing Guo, Chunbiao Li, Lei Shi, Jiaoyuan Zhang, Xingna Cai, Zukang Chen, Qian Zhang, Jun Du, Changyuan Tao, Qizhi Chen, Guocan Zheng, Zuohua Liu

**Affiliations:** ^1^ School of Chemistry and Chemical Engineering Chongqing University Chongqing China; ^2^ School of Artificial Intelligence Nanjing University of Information Science and Technology Nanjing China; ^3^ Guangxi Huiyuan Manganese Industry Co., Ltd Laibin China; ^4^ School of Chemistry and Chemical Engineering Guangxi University Nanning China

**Keywords:** chaotic suspension electrolysis, defect engineering, scalable manufacturing, thermal safety evaluation, Zn‐MnO_2_ batteries

## Abstract

High‐performance MnO_2_ for aqueous zinc‐manganese batteries (AZMBs) is still predominantly produced through laboratory‐scale syntheses that deliver low output and are difficult to scale, creating a persistent barrier to practical deployment. Here, we introduce a “programmable chaotic suspension electrolysis” strategy that incorporates nonlinear dynamics into the electrodeposition process to achieve macroscopic production of MnO_2_ with precise microstructural control. Compared with conventional synthesis routes, this approach increases the MnO_2_ yield by orders of magnitude. Furthermore, by regulating the aperiodic oscillations of the chaotic current, we induce abundant, tunable in situ oxygen vacancies and construct a robust γ/β intergrown tunnel framework. This distinct defect engineering significantly enhances the electrochemical activity of the material. Additionally, we establish a multi‐dimensional evaluation framework that confirms the superior thermal stability of the optimized product. Coupled with Life Cycle Assessment (LCA) and Techno‐Economic Analysis (TEA), the process demonstrates significant potential for reducing energy consumption and production costs. Overall, this work provides a generalizable pathway for the scalable manufacturing of high‐performance electrode materials and highlights the promise of chaotic electrochemistry in constructing next‐generation safe, low‐cost energy storage systems.

## Graphical abstract

Programmable chaotic suspension electrolysis bridges laboratory synthesis and industrial manufacturing of high‐performance MnO_2_ cathodes. This scalable strategy enables tunable oxygen vacancies and a robust γ/β tunnel framework, delivering superior electrochemical performance and thermal stability. Pilot‐scale validation, LCA, and TEA confirm significant reductions in energy consumption, CO_2_ emissions, and production costs.

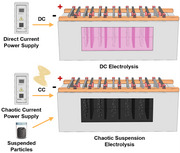

## 1 Introduction

Aqueous zinc‐manganese batteries (AZMBs) have emerged as frontrunners for next‐generation energy storage owing to their intrinsic cost‐effectiveness and environmental benignity [1, 2, 3, 4, 5]. Central to the performance of these devices is the structural optimization of the MnO_2_ cathode [6, 7, 8]. However, while academia has made significant strides in material design, current high‐performance fabrication processes remain heavily reliant on traditional solvothermal synthesis and rudimentary beaker‐type reactions [9, 10, 11, 12]. These laboratory‐scale methods are universally plagued by extremely low yields (milligram‐scale), discontinuous batch processing, and safety hazards associated with high‐temperature and high‐pressure reactors [13, 14, 15, 16]. Such intrinsic limitations severely impede the feasibility of transitioning high‐performance cathodes from the laboratory to scalable production. Consequently, developing a novel manufacturing process that combines precise microstructural regulation with macroscopic scalability is a prerequisite for the commercialization of high‐performance AZMBs.

Electrodeposition is inherently a non‐equilibrium, nonlinear dynamic process [17, 18]. Achieving precise control over microcrystalline growth within macroscopic electrolysis requires dynamic perturbations that transcend traditional direct or pulsed currents. Drawing upon Prigogine's nonequilibrium thermodynamics and modern nonlinear chemical dynamics, the introduction of “deterministic chaos” offers a novel regulatory dimension for electrochemical systems [19, 20]. Unlike periodic waveforms, chaotic signals possess infinite non‐periodicity and extreme sensitivity to initial conditions, enabling effective perturbation of the electric double layer and significant alteration of ion transport and nucleation kinetics [21, 22, 23, 24]. Coupling a controllable chaotic current into a suspension electrolysis system induces abundant oxygen vacancies in situ while maintaining a high‐crystallinity framework, thereby achieving microstructural tailoring at a macroscopic scale that is unattainable by conventional methods.

Herein, we propose a programmable chaotic suspension electrolysis strategy, successfully bridging the gap between laboratory synthesis and industrial manufacturing. Utilizing a chaotic circuit to drive suspension electrolysis, this method achieves kilogram‐scale single‐plate deposition of MnO_2_, increasing yields by orders of magnitude compared to traditional hydrothermal routes, and has been validated via industrial pilot testing. By tuning the amplitude of the chaotic current, we achieve “programmable” regulation of the oxygen vacancy concentration, yielding an optimized product with a robust γ/β intergrown tunnel framework (CS‐M4).

This scalably manufactured material demonstrates superior application potential: CS‐M4 exhibits excellent rate capability and cycling stability in AZMBs, as well as significantly enhanced discharge capacity in commercial alkaline Zn‐MnO_2_ batteries (LR6). Furthermore, we propose two universal metrics to quantify the thermal stability of electrode materials, highlighting the safety advantages of this system for large‐scale energy storage. Comprehensive Life Cycle Assessment (LCA) and Techno‐Economic Analysis (TEA) further confirm that this process significantly reduces energy consumption, carbon footprint, and production costs. Taken together, this work not only provides a scalable route for high‐performance electrode manufacturing but also underscores the immense potential of chaotic electrochemistry in realizing sustainable, low‐cost energy storage.

## 2 Results and Discussion

### 2.1 Programmable Chaotic Suspension Electrolysis System

We initially evaluated the impact of conventional electrolysis parameters—including H^+^ concentration, current density, temperature, and anode material—on the product structure. X‐ray diffraction (XRD) analysis revealed that manipulating these variables yielded exclusively γ‐phase MnO_2_ with limited crystallinity, which is insufficient for high‐performance AZMBs (Figure S1). To overcome this limitation, we implemented a suspension electrolysis strategy, screening four common MnO_2_ polymorphs (α, β, δ, and γ) as suspending agents. The results demonstrated that only β‐MnO_2_ effectively induced the emergence of characteristic β‐phase diffraction peaks, resulting in a highly crystalline γ/β mixed‐phase structure (Figure S2). Further optimization of the suspending agent concentration revealed a significant enhancement in crystallinity with increasing β‐MnO_2_ dosage. Scanning electron microscopy (SEM) indicated that the product surface evolved from a relatively smooth texture to a dense, needle‐like architecture, with both crystal structure and micromorphology stabilizing at a concentration of 0.8 g L^−1^ (Figure S3) [25]. Consequently, a 0.8 g L^−1^ β‐MnO_2_ suspension system (denoted as S‐M) was established as the optimal baseline for the subsequent introduction of chaotic current regulation.

**FIGURE 1:**
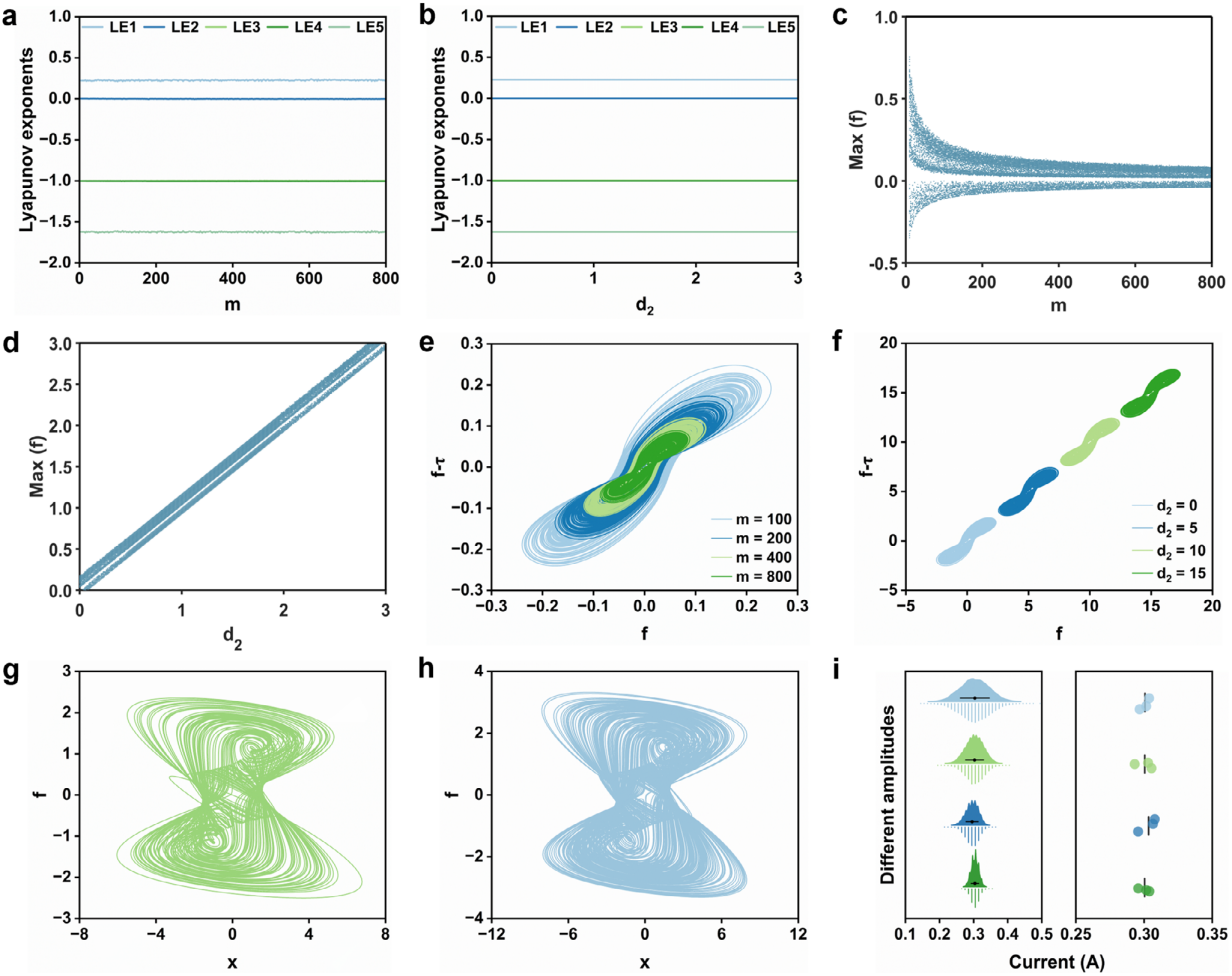
Parameter control and experimental validation of the chaotic system. (a, b) Influence of parameters *m* and *d*_2_ on the system's Lyapunov exponents. (c, d) Influence of parameters *m* and *d*_2_ on the system's bifurcation diagrams. (e) Amplitude control of signal *f* under different *m* values. (f) Bias control of signal *f* under different *d*_2_ values. (g) Phase portrait of signals *x* and *f* from numerical simulation (step size 0.005). (h) Phase portrait of electrical signals *x* and *f* from circuit simulation. (i) Electrical signal acquisition and mean value analysis for CS‐M under different chaotic‐current amplitudes.

To facilitate precise control over the suspension electrolysis process and circumvent the limitations of existing chaotic circuits regarding hardware implementation, signal amplification, and parameter precision, we constructed a novel five‐dimensional chaotic system (Equation 1) [26]. Lyapunov exponent analysis confirms that variations in system parameters *m* and *d*_2_ do not perturb the chaotic state, as the exponents remain invariant (Figure 1a,b). Bifurcation diagrams further elucidate the regulatory mechanisms: increasing *m* compresses the fluctuation amplitude of signal *f*, enabling amplitude modulation, whereas adjusting *d*_2_ induces a linear baseline shift in *f* without altering its amplitude, thereby achieving bias control (Figure 1c,d) [27]. Time‐delay phase portraits visually corroborate these findings, demonstrating that the topological structure of the chaotic attractors remains stable across varying *m* and *d*_2_ values, exhibiting solely dimensional scaling or positional translation (Figure 1e,f and Figure S4). These results establish a robust theoretical foundation for the independent regulation of chaotic signal amplitude and intensity via *m* and *d*_2_. Furthermore, the dynamical effects of other system parameters (*a*, *b*, *c*) and multistability characteristics were comprehensively assessed (Figures S5, S6 and Note S1).

(1) $$\left\{\begin{array}{c} \;\dot{x}=az-byz\\ \;\dot{y}=mz^{2}-cy\\ \;\dot{z}=x-z+d_{1}\\ w=y-w\\ f=z-f+d_{2} \end{array}\right.$$

The system exhibits chaotic behavior when parameters are set to *a* = 4, *b* = 1, *c* = 0.4, *m* = 1, *d*_1_ = 0, *d*_2_ = 0, with initial conditions (0.1, 0.1, 0.1, 0.1, 0.1).

Based on this mathematical model, we derived the corresponding circuit equations (Equation 2) and designed a simulation circuit (Figure S7). Numerical and circuit simulations exhibited exceptional consistency across the *x*‐*f*, *y*‐*f*, *z*‐*f*, and *w*‐*f* phase planes (Figure 1g,h and Figure S8), confirming the high fidelity of the simulated signals to the theoretical model. Ultimately, a hardware circuit was physically implemented; the measured waveform of signal *f* and its phase trajectory with *x* aligned perfectly with the simulation results (Figure S9), verifying the successful physical realization of the controllable chaotic system.

(2) $$\left\{\begin{array}{c} \;\frac{dx}{dt}=\frac{1}{C_{1}}\;\left(K\frac{-yz}{R_{1}}+\frac{z}{R_{2}}\right)\\ \;\frac{dy}{dt}=\frac{1}{C_{2}}\;\left(K\frac{z^{2}}{R_{6}}-\frac{y}{R_{5}}\right)\\ \;\frac{dz}{dt}=\frac{1}{C_{3}}\;\left(\frac{x}{R_{9}}-\frac{z}{R_{11}}-\frac{V_{3}}{R_{10}}\right)\\ \;\frac{dw}{dt}=\frac{1}{C_{4}}\;\left(\frac{y}{R_{14}}-\frac{w}{R_{15}}\right)\\ \;\frac{df}{dt}=\frac{1}{C_{5}}\;\left(\frac{z}{R_{18}}-\frac{f}{R_{19}}-\frac{V_{4}}{R_{20}}\right) \end{array}\right.$$

Here, *K* represents the gain coefficient, with a value of 0.1 in the equation.

Leveraging the controllable chaotic system, we integrated chaotic currents into the suspension electrolysis regime. By modulating circuit parameter *m*, four distinct chaotic signal amplitudes were applied to synthesize samples designated as CS‐M1 through CS‐M4. To ensure data reliability, each amplitude condition was validated via three independent experimental replicates (Figure 1i and Figure S10). X‐ray photoelectron spectroscopy (XPS) analysis revealed that the chaotic current in situ induces oxygen vacancies within the EMD lattice; the vacancy concentration scales positively with current amplitude, concomitant with a reduction in the average manganese oxidation state (Figure S11). This finding was further corroborated by electron paramagnetic resonance (EPR) spectroscopy, which revealed a consistent trend in defect signal intensity (Figure S12). To address the substantial material requirements for subsequent high‐performance AZMB pouch cells and commercial LR6 batteries, we scaled up the process, achieving a stable single‐plate yield of 1089.78 g of MnO_2_ (Figure S13). This production capacity far exceeds that of conventional hydrothermal synthesis and offers significant advantages regarding reduced cell voltage and low‐temperature operation (discussed below), highlighting its immense potential for industrial manufacturing.

### 2.2 Microstructural Evolution and Defect Engineering

**FIGURE 2:**
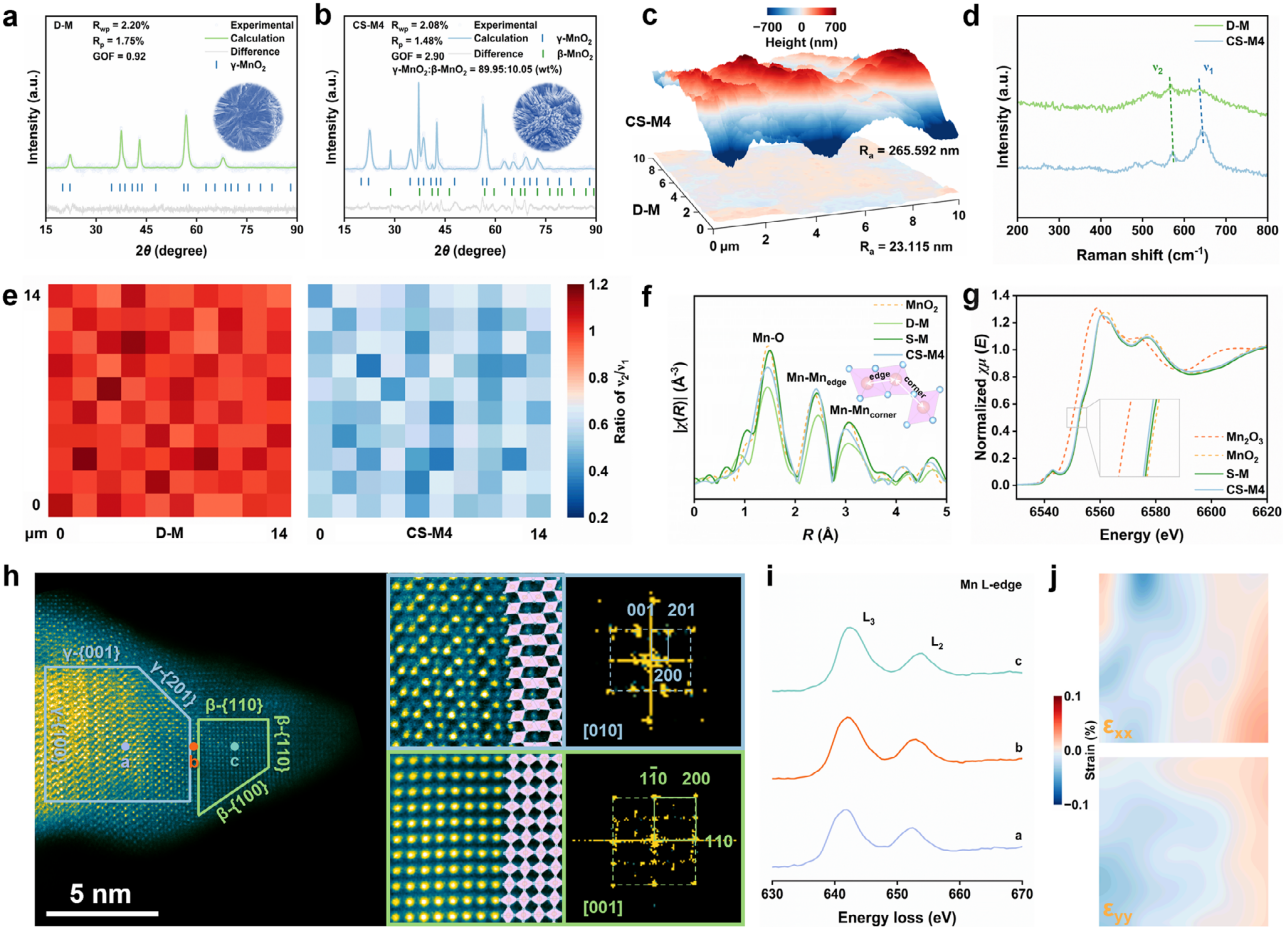
Structural and compositional characterization of EMD. (a, b) Rietveld‐refined XRD patterns of D‐M and CS‐M4. (c) AFM three‐dimensional topography images. (d) Raman spectra. (e) Heat maps of the *v*_2_/*v*_1_ intensity ratio distribution obtained from Raman mapping. (f) Mn K‐edge EXAFS spectra. (g) Mn K‐edge XANES spectra. (h) HAADF‐STEM image of CS‐M4 and atomic‐resolution HAADF‐STEM images with corresponding FFT patterns from the regions marked by blue and green squares. (i) Mn L_2, 3_‐edge EELS spectra acquired from different positions (points a, b and c) indicated in panel (h). (j) GPA strain distribution maps (ε_xx_ and ε_yy_) across the phase boundary region.

Rietveld‐refined XRD patterns (Figure 2a,b) indicate that CS‐M4 has markedly superior crystallinity compared to D‐M and displays a mixed γ/β‐phase structure. SEM images (insets) reveal that CS‐M4 exhibits a densely packed, pine‐needle‐like surface shape, signifying an optimal MnO_2_ growth mode prompted by the chaotic suspension electrolysis process (Figure S14) [28, 29]. Atomic force microscopy (AFM) 3D topography reveals that the surface roughness of both CS‐M4 and S‐M significantly exceeds that of D‐M (Figure 2c and Figure S15). This morphology is attributed to the suspension agent providing abundant nucleation sites, which increases the deposition‐specific surface area and reduces the local current density, thereby facilitating the growth of highly crystalline MnO_2_. Furthermore, conductive AFM (CAFM) measurements confirm a heightened current response signal in CS‐M4, indicating a substantial enhancement in electrical conductivity compared to D‐M (Figure S16).

Raman spectroscopy unveils significant disparities in local structural order between D‐M and CS‐M4 (Figure 2d). The peaks located at 640–670 cm^−1^ (*v*_1_, A_1g_ symmetry mode) and 560–580 cm^−1^ (*v*_2_) correspond to the symmetric and asymmetric stretching vibrations of [MnO_6_] octahedra, respectively, with the *v*_1_ intensity being highly sensitive to long‐range lattice order [30, 31]. Raman mapping results indicate a markedly lower *v*_2_/*v*_1_ intensity ratio for CS‐M4 compared to D‐M (Figure 2e), signifying superior crystallinity and a more ordered arrangement of [MnO_6_] octahedra [32].

Synchrotron x‐ray absorption fine structure (XAFS) further elucidates the regulatory mechanism of the chaotic current on the local atomic environment. The Mn K‐edge extended x‐ray absorption fine structure (EXAFS) spectra (Figure 2f) reveal that CS‐M4 exhibits Mn–Mn*_edge_* and Mn–Mn*_corner_* bonding characteristics highly similar to S‐M. This indicates that the chaotic current preserves the high‐crystallinity framework, maintaining structural stability. In contrast, D‐M displays significantly weaker bonding intensities, implying insufficient stability and poor connectivity of the [MnO_6_] octahedra, which predisposes the structure to collapse during cycling; this finding is corroborated by wavelet transform analysis (Figure S17). Conversely, the Mn K‐edge x‐ray absorption near‐edge structure (XANES) spectra show a shift of the CS‐M4 absorption edge toward lower energy relative to S‐M (Figure 2g), confirming that the introduction of oxygen vacancies reduces the average Mn valence, consistent with the preceding XPS results [33].

High‐angle annular dark‐field scanning transmission electron microscopy (HAADF‐STEM) images clearly visualize the coexistence of γ‐MnO_2_ [1 × 1] + [1 × 2] tunnels and β‐MnO_2_ [1 × 1] tunnels within CS‐M4 (Figure 2h and Figures S18 and S19), aligning perfectly with the XRD refinement [34, 35, 36]. Electron energy loss spectroscopy (EELS) analysis reveals constant Mn L_2, 3_‐edge peak positions across the γ‐phase region (point a), phase boundary (point b), and β‐phase region (point c) (Figure 2i), confirming the uniformity of Mn valence distribution and structural‐chemical stability. Geometric phase analysis (GPA) demonstrates no significant lattice distortion in either CS‐M4 or S‐M (Figure 2j and Figure S20a), indicating that the incorporation of the β‐phase establishes a robust interfacial structure characterized by high crystallinity and minimal lattice strain; in contrast, D‐M exhibits multiple regions of lattice deformation and stress concentration (Figure S20b).

### 2.3 Electrochemical Performance and Mechanism Insight

To assess the practical applicability of the scalable EMD, we fabricated laboratory‐scale coin cells, high‐loading pouch cells for next‐generation energy storage, and LR6 batteries using both D‐M and CS‐M4 cathodes.

**FIGURE 3:**
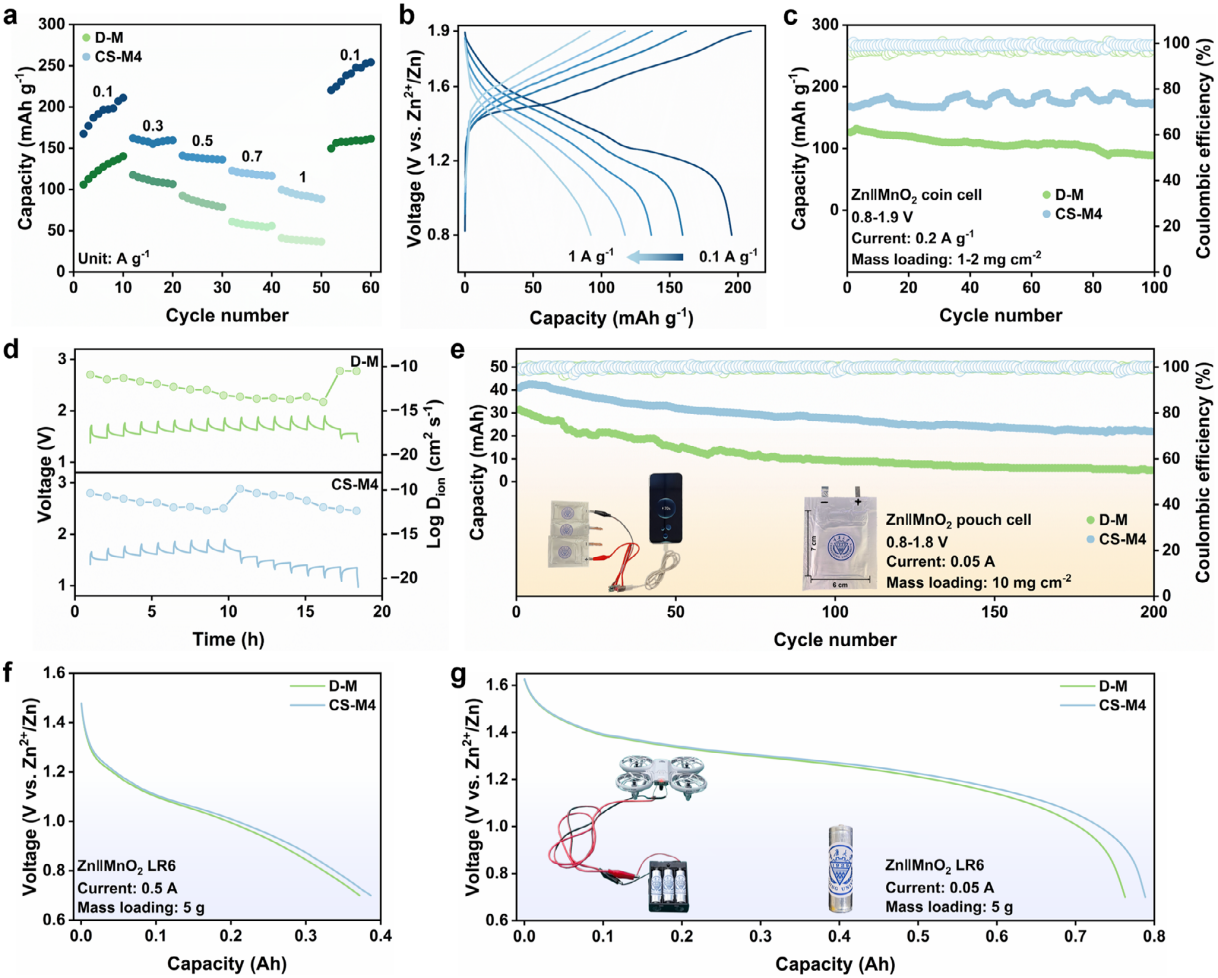
EMD battery performance tests. (a) Rate performance. (b) GCD profiles of CS‐M4 at different current densities. (c) Cycling performance of coin cells. (d) CS‐M4 and D‐M GITT profiles and corresponding ion diffusion coefficients. (e) Cycling performance of pouch cells. (f) Discharge curves of alkaline LR6 batteries under high‐current conditions. (g) Discharge curves of alkaline LR6 batteries under low‐current conditions.

Coin cell testing demonstrated that the CS‐M4 cathode delivers significantly superior specific discharge capacities relative to D‐M across current densities from 0.1 to 1 A g^−1^ (Figure 3a). Upon returning to 0.1 A g^−1^, CS‐M4 recovered a capacity of 239.66 mAh g^−1^, far exceeding the 157.75 mAh g^−1^ of D‐M, highlighting its robust rate capability and structural reversibility. The corresponding galvanostatic charge‐discharge (GCD) profiles are presented in Figure 3b and Figure S21. During extended cycling at 0.2 A g^−1^ (Figure 3c), CS‐M4 retained a capacity of 179.84 mAh g^−1^ after 100 cycles with negligible degradation, whereas D‐M decayed to 88.98 mAh g^−1^ (70.68% retention). The GCD profiles at various cycle numbers are detailed in Figure S22. This superior performance is attributed to the robust tunnel architecture and abundant active sites engendered by the chaotic suspension electrolysis. Furthermore, CS‐M4 outperforms other hydrothermally synthesized MnO_2_ polymorphs (α‐, β‐, and δ‐MnO_2_) in both cycling stability and specific capacity (Figure S23 and Table S1) [37, 38, 39].

To investigate the underlying electrochemical kinetics, galvanostatic intermittent titration technique (GITT) measurements were conducted. The results indicate that CS‐M4 possesses higher ion diffusion coefficients than D‐M throughout the charge–discharge process (Figure 3d), confirming faster ion migration kinetics. This suggests that the oxygen vacancies and optimized crystal structure introduced by chaotic electrolysis effectively lower the ion diffusion barrier, thereby enhancing rate performance and reaction kinetics [40, 41].

To evaluate scalability, pouch cells were assembled with a cathode loading of 10 mg cm^−2^. At a current of 0.05 A, the CS‐M4 cell delivered a higher initial capacity of 42.56 mAh compared to 31.5 mAh for D‐M and maintained stable performance over 200 cycles, whereas the D‐M cell suffered significant capacity decay (Figure 3e). The insets display the pouch cell dimensions and a demonstration powering a mobile phone using three cells in series [42, 43]. Furthermore, when integrated into LR6 batteries, CS‐M4 demonstrated superior discharge capabilities: delivering 0.39 Ah at a high current of 0.5 A and 0.79 Ah at a low current of 0.05 A (Figure 3f,g). The insets illustrate the fabricated LR6 battery and a drone powered by three LR6 cells in series. These results verify that CS‐M4 is fully compatible with commercial LR6 assembly processes and significantly boosts the performance of alkaline Zn‐MnO_2_ batteries.

**FIGURE 4:**
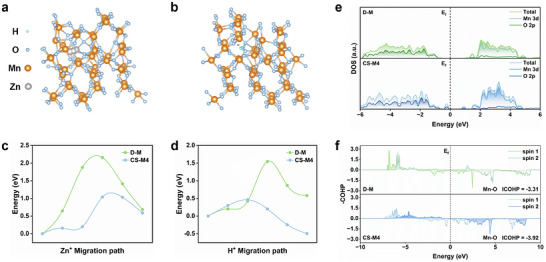
Theoretical analysis of D‐M and CS‐M4. (a, b) Schematic illustrations of optimal migration pathways for Zn^2+^ and H^+^ within the CS‐M4 lattice. (c, d) Comparison of migration energy barriers for Zn^2+^ and H^+^ in D‐M vs. CS‐M4. (e) Comparison of TDOS and PDOS for D‐M and CS‐M4. (f) COHP analysis of Mn–O bonds in D‐M and CS‐M4.

To elucidate the intrinsic electrochemical mechanism of CS‐M4 at the microscopic level, we performed first‐principles calculations based on density functional theory (DFT). First, the optimal diffusion pathways for Zn^2+^ and H^+^ within the tunnel structures of CS‐M4 and D‐M were simulated (Figure 4a,b and Figure S24). The results indicate that the open tunnel architecture and defect sites engineered by chaotic suspension electrolysis significantly lower the ion diffusion barriers. Specifically, the migration barrier for Zn^2+^ in CS‐M4 is only 1.04 eV, markedly lower than the 2.16 eV observed in D‐M (Figure 4c); similarly, the H^+^ migration barrier is reduced substantially from 1.55 eV in D‐M to 0.46 eV in CS‐M4 (Figure 4d). These exceptionally low barriers directly confirm the accelerated ion transport kinetics in CS‐M4, which aligns highly with the preceding GITT results [44].

Beyond optimizing ion kinetics, regulating the electronic structure is equally critical. Figure 4e presents the total and projected density of states (TDOS and PDOS) for D‐M and CS‐M4. Compared to D‐M, CS‐M4 exhibits a narrower band gap and a higher density of states near the Fermi level. This electronic optimization is primarily attributed to the redistribution of local charge density induced by oxygen vacancies, which substantially boosts intrinsic electronic conductivity, consistent with the high current response observed in CAFM measurements [45, 46].

Finally, to decipher the mechanism underlying long‐term cycling stability, the bonding characteristics of Mn─O bonds were analyzed using the crystal orbital Hamiltonian population (COHP) method (Figure 4f). The COHP analysis confirms that, despite the presence of oxygen vacancies, the Mn─O bonding strength in CS‐M4 remains superior to that in D‐M. This reinforced Mn─O bonding network imparts greater rigidity to the framework, effectively suppressing structural distortion caused by the Jahn‐Teller effect, thereby ensuring structural integrity during prolonged cycling [47].

### 2.4 Thermal Stability and Kinetic Analysis

**FIGURE 5:**
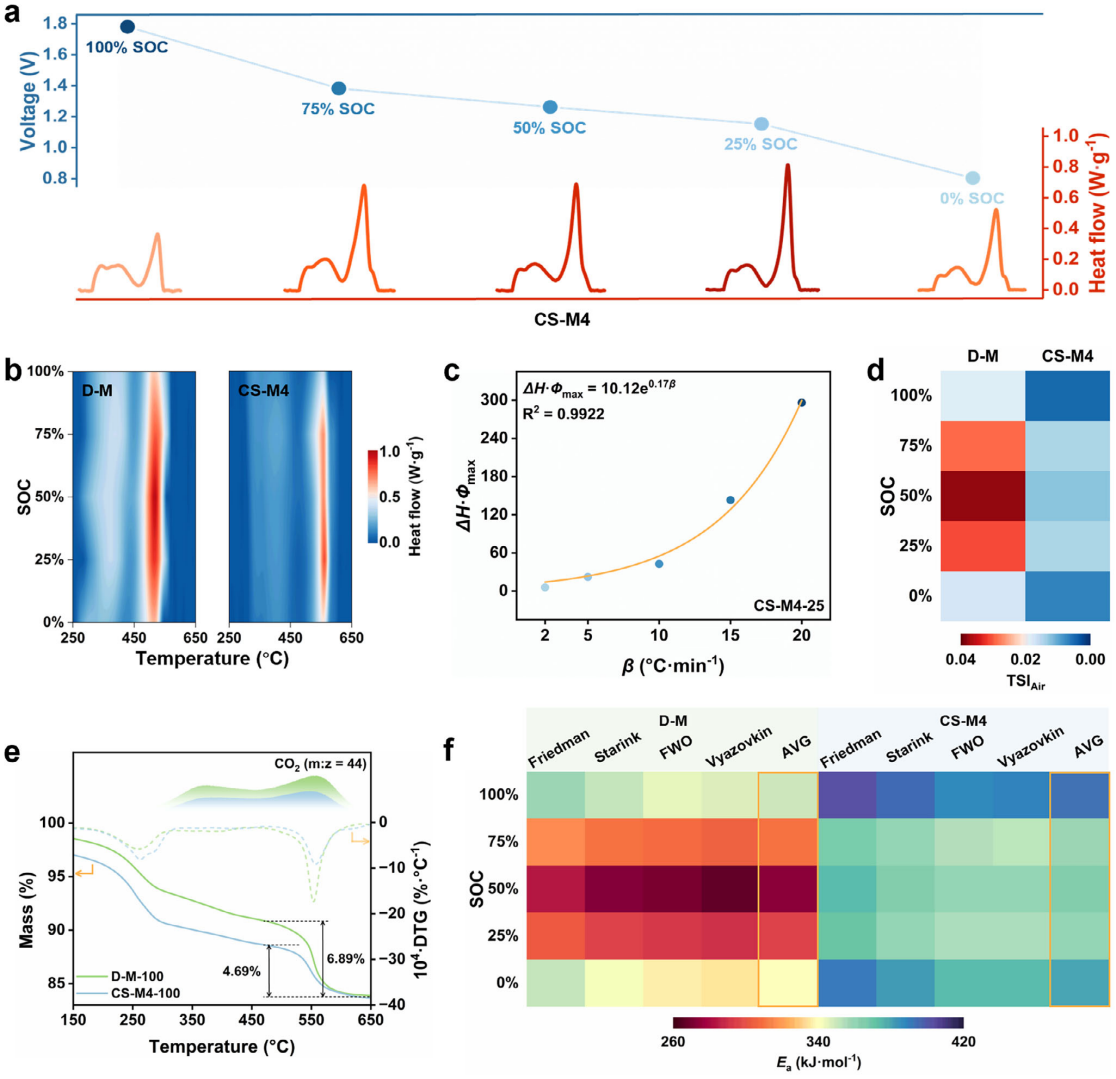
Thermal analysis of battery cathode materials. (a) DSC curves of CS‐M4 at different SOCs. (b) Evolution of DSC curves with SOC for D‐M and CS‐M4. (c) Values of Δ*H*·*Ф*_max_ for CS‐M4 at 25% SOC under different *β* and their fitting curve. (d) TSI_Air_ heat maps for D‐M and CS‐M4 at different SOCs. (e) TG‐MS analysis of D‐M and CS‐M4 at 100% SOC. (f) Heat maps of *E*_a_ for D‐M and CS‐M4 at different SOCs calculated using different kinetic models and their average values.

While aqueous electrolytes endow AZMBs with intrinsic safety superior to organic systems, a rigorous assessment of thermal stability remains imperative to ensure absolute reliability in large‐scale energy storage; yet, systematic investigations into the thermal safety of aqueous batteries remain scarce. To address this gap, differential scanning calorimetry (DSC) measurements were conducted on D‐M and CS‐M4 pouch cells at various states of charge (SOC) after 10 cycles (Figure S25). As illustrated in Figure 5a, CS‐M4 exhibits a notably lower enthalpy change (Δ*H*) at both 100% and 0% SOC. Analyzing the evolution of heat flow with SOC (Figure 5b) reveals that the peak temperature (*T*_p_) of CS‐M4 is consistently 40°C higher than that of D‐M across all SOCs (Figure S26), accompanied by a reduced maximum heat flow (*Φ*_max_), preliminarily indicating superior thermal stability.

However, relying on single conventional thermal stability parameters (Δ*H*, *Φ*_max_, and *T*_p_) is often insufficient for accurately assessing practical thermal stability, as these values are contingent upon experimental conditions such as the heating rate (*β*). Indeed, the DSC profiles of the CS‐M4 cathode (25% SOC) exhibit significant variation with *β* (Figure S27). We identified a distinct exponential relationship between Δ*H*·*Φ*_max_ and *β* (Figure 5c) and subsequently introduced a correction factor, e^0.17^ *^β^*, to propose a normalized evaluation metric, TSI_Air_ (where the subscript denotes the experimental atmosphere):

(3) $$TSI_{Air}=\frac{\Delta H\cdot \phi_{max}}{T_{P}\cdot e^{0.17\beta }}$$

This metric effectively eliminates the dependency on *β*, rendering the evaluation more objective and universal. As shown in Figure 5d and Figure S28, compared to conventional parameters, TSI_Air_ provides a more effective and comprehensive differentiation of thermal stability across various materials and SOCs [48].

To investigate the thermal decomposition mechanism from the perspective of chemical bond scission, thermogravimetry‐mass spectrometry (TG‐MS) analysis was performed on D‐M and CS‐M4 at 100% SOC [49]. During the second weight‐loss stage, corresponding to Mn─O bond dissociation, CS‐M4 exhibited a significantly lower weight loss (4.69%) compared to D‐M (6.89%), accompanied by reduced CO_2_ evolution (Figure 5e). This directly corroborates the superior thermal stability of the Mn─O bonds in CS‐M4, aligning perfectly with the theoretical COHP calculations discussed earlier, thereby achieving a mutual validation between macroscopic thermal analysis and microscopic chemical bonding theory.

To further quantify the resistance to thermal runaway from a reaction kinetics perspective, the activation energy (*E*_a_) parameter was introduced. According to the consensus of the International Confederation for Thermal Analysis and Calorimetry (ICTAC), constructing reliable kinetic models necessitates experimental data acquired at multiple heating rates [50, 51, 52]. Given the susceptibility of DSC to signal fluctuations in weakly exothermic systems, we selected the more robust thermogravimetric (TG) data as the basis for kinetic analysis (Figure S29). Using data collected at various heating rates (*β* = 2, 5, 10, 15, and 20°C min^−1^) and selecting ten specific conversion points (*α* = 0.1–0.9, and 0.95), we employed four complementary kinetic models to calculate *E*_a_ (Table S2) for cross‐validation. Specifically, the Friedman and Starink methods are derived from the Arrhenius equation; the former utilizes a differential model obtained by taking the logarithm of the reaction rate equation, while the latter is an improved form of the KAS method that enhances fitting precision via a corrected exponent. The FWO method serves as an integral model based on the linear relationship between lg*β* and 1/*T* at a constant *α*, whereas the Vyazovkin method represents a model‐free integral isoconversional technique that determines *E*_a_ by minimizing the difference in kinetic functions across different temperature paths [53, 54, 55]. The specific equations are:

Friedman model:

(4) $$\ln\frac{\beta d\alpha }{dT}=\ln\left[Af\left(\alpha \right)\right]\;-\frac{E_{a}}{RT}$$

Starink model:

(5) $$\ln\;\left(\frac{\beta }{T^{1.92}}\right)=C_{s}\;-1.0037\frac{E_{a}}{RT}$$

FWO model:

(6) $$\mathrm{lg}\beta =\mathrm{lg}\left(\frac{AE_{a}}{RG\left(\alpha \right)}\right)-2.315-0.4567\frac{E_{a}}{RT}\;$$

Vyazovkin model:

(7) $$-\ln\;t_{\alpha ,t}=\ln\left[\frac{A}{G\left(\alpha \right)}\right]-\frac{E_{a}}{RT}\;$$

where *T* is temperature (K), *R* is the gas constant (8.314 J mol^−1^ K^−1^), *A* is the pre‐exponential factor, and *t_α_*_,_ *_t_* is the time (s) required to reach a specific *α*. The calculated mean *E*_a_ values are visualized as a heatmap in Figure 5f, with detailed calculation procedures and *α*‐*E*_a_ correlations provided in Figures S30–S33 and Note S2. Both independent assessment methods—TSI_Air_ and *E*_a_—consistently indicate the superior thermal stability of CS‐M4 pouch cells over D‐M. This convergence not only confirms the rationality and reliability of these metrics but also suggests their broader applicability for evaluating thermal safety in other battery chemistries. Notably, we identify 100% SOC as the most thermally stable state for AZMBs—a counterintuitive finding that contrasts sharply with the behavior of lithium‐ion batteries and has been rarely reported. This underscores the unique safety advantages and significant potential of AZMBs for large‐scale energy storage applications.

### 2.5 Industrial Scalability and Sustainability

**FIGURE 6:**
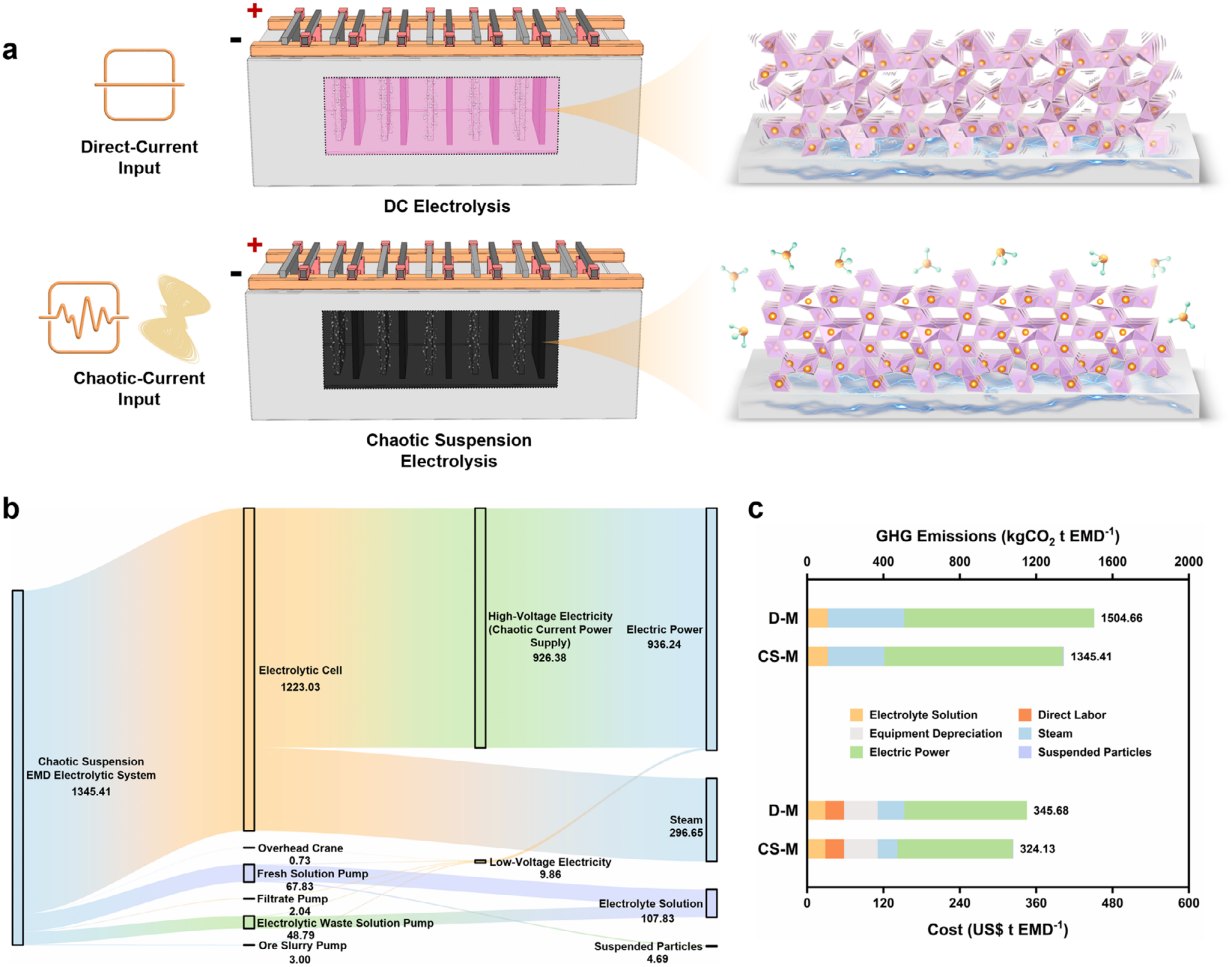
Assessment of industrial sustainability and economic feasibility. (a) Schematic illustration of the reaction processes of MnO_2_ under direct‐current electrolysis and chaotic suspension electrolysis. (b) Sankey diagram of CO_2_ emission analysis for the chaotic suspension electrolysis process. (c) Greenhouse gas emissions and cost analysis comparing the conventional and chaotic suspension electrolysis processes.

To evaluate the industrial potential and environmental benefits of the chaotic suspension electrolysis strategy, we established a comprehensive evaluation framework spanning mechanistic verification to pilot‐scale testing, LCA, and TEA. As illustrated in Figure 6a, this strategy fundamentally reshapes electrolysis kinetics through the synergistic action of chaotic currents and suspended particles.

The aperiodic oscillations induced by chaotic signals significantly enhance mass transfer within the electrolyte, effectively mitigating concentration polarization. Averaged results from three independent replicates indicate that, at identical current densities, the mean cell voltage for CS‐M4 preparation is reduced by 6.01% compared to D‐M (Figure S34). Simultaneously, the suspended particles serve as extrinsic nucleation sites, drastically improving product crystallinity and obviating the reliance on high‐temperature electrolysis (>95°C). Consequently, CS‐M4 preparation can be conducted at a reduced temperature of 80°C, effectively lowering steam heating consumption. Experiments demonstrate that at this temperature, the product purity remains stably above 92%—superior to traditional EMD—with excellent Faradaic efficiency (Figure S35 and Note S3).

To validate scalability, we successfully conducted pilot‐scale preparation (Figure S36), confirming the robustness of this strategy in a macroscopic manufacturing scenario. We compared the CO_2_ emission Sankey diagrams of traditional DC electrolysis (Figure S37) and chaotic suspension electrolysis (Figure 6b). The analysis reveals that the chaotic suspension approach significantly lowers the carbon footprint per unit product, driven by the synergistic reduction in cell voltage and operating temperature. Comprehensive LCA and TEA assessments (Figure 6c) indicate that producing one metric ton of CS‐M4 reduces CO_2_ emissions by 159.25 kg and saves USD 21.55 [56, 57, 58]. Furthermore, accounting for regional variations in global energy prices, we performed a sensitivity analysis on electricity and steam costs based on 2025 pricing data from major EMD‐producing nations and actual industrial procurement figures (Figure S38) [59, 60]. Extrapolating this paradigm to the global EMD industry (annual capacity 716.8 kilotons) suggests a theoretical annual reduction of 114.1 kilotons of CO_2_ emissions and economic savings of USD 15.45 million. These results compellingly demonstrate that chaotic suspension electrolysis serves as a universal solution combining high‐performance manufacturing capability with environmental and economic sustainability.

## 3 Conclusions

This study pioneers a “programmable chaotic suspension electrolysis” strategy that integrates nonlinear dynamics into electrochemical deposition, achieving atomic‐level precision tailoring of electrode microstructures at a macroscopic scale. Relative to conventional laboratory synthesis, this method boosts MnO_2_ yield by orders of magnitude and has been validated via industrial pilot testing. The resulting optimized product features abundant in situ oxygen vacancies and a robust γ/β intergrown tunnel framework. This architecture endows AZMBs with superior rate capability and cycling stability while significantly enhancing the discharge capacity of LR6 batteries. Addressing the critical gap in thermal safety research for aqueous batteries, we established a systematic evaluation framework based on a normalized metric (TSI_Air_) and multiple kinetic models (*E*_a_). This assessment confirms the superior thermal stability of CS‐M4 and establishes a methodological framework that may serve as a reference for evaluating thermal safety in other battery systems. Furthermore, LCA and TEA demonstrate that this strategy achieves significant energy savings by synergistically lowering the average cell voltage and electrolysis temperature. Extrapolating this paradigm to the global EMD sector (annual capacity of 716.8 kilotons) suggests a potential annual reduction of approximately 114.1 kilotons in CO_2_ emissions and cost savings of USD 15.45 million. In summary, this work not only propels the holistic upgrade of Zn‐Mn battery technology but also underscores the transformative potential of chaotic electrochemistry in realizing next‐generation low‐cost, high‐safety, and sustainable energy storage systems.

## Author Contributions

Z.L. proposed the research direction and supervised the project. Z.W. designed and performed the experiments. J.Y. contributed to the theoretical calculation and experimental analysis. J.Z., X.C., and Q.Z. supported the ECA, LCA, and TEA. L.S., W.L., J.L., and Y.F. assisted with parts of the experiments and data analysis. J.G., Z.C., J.D., and C.T. contributed to data curation and validation. Q.C., G.Z., and C.L. contributed to theoretical and model development and supported data analysis. Z.W., J.Y., and Z.L. discussed the results and co‐wrote the manuscript. All authors participated in the discussion and the preparation of the manuscript.

## Conflicts of Interest

The authors declare no conflicts of interest.

## Supporting information




**Supporting File**: advs76766‐sup‐0001‐SuppMat.docx.

## Data Availability

The data that support the findings of this study are available from the corresponding author upon reasonable request.

## References

[1] X. Jia , C. Liu , Z. G. Neale , J. Yang , and G. Cao , “Active Materials for Aqueous Zinc Ion Batteries: Synthesis, Crystal Structure, Morphology, and Electrochemistry,” Chemical Reviews 120, no. 15 (2020): 7795–7866, 10.1021/acs.chemrev.9b00628. 32786670

[2] Y. Liao , H.‐C. Chen , C. Yang , et al., “Unveiling Performance Evolution Mechanisms of MnO_2_ Polymorphs for Durable Aqueous Zinc‐Ion Batteries,” Energy Storage Materials 44 (2022): 508–516, 10.1016/j.ensm.2021.10.039.

[3] H. Luo , H. Guo , X. Li , et al., “Aqueous Manganese‐Ion Batteries: The Past, Present, and Future,” Matter 8 (2025): 102379.

[4] Y. Yuan , K. He , and J. Lu , “Interfacial Chemistry in Rechargeable Batteries: From Fundamentals to Applications,” Angewandte Chemie International Edition 63 (2023): 202213791.

[5] N. Zhang , Y.‐R. Ji , J.‐C. Wang , P.‐F. Wang , Y.‐R. Zhu , and T.‐F. Yi , “Understanding of the Charge Storage Mechanism of MnO_2_‐Based Aqueous Zinc‐Ion Batteries: Reaction Processes and Regulation Strategies,” Journal of Energy Chemistry 82 (2023): 423–463, 10.1016/j.jechem.2023.03.052.

[6] J. Chacón‐Borrero , X. Chang , Z. Min , et al., “Boosting High‐Loading Zinc‐Ion Battery Performance: Zn‐Doped δ‐MnO_2_ Cathodes to Promote Zn^2+^ Storage,” Energy Storage Materials 81 (2025): 104486.

[7] X. Zhao , F. Zhang , H. Li , et al., “Dynamic Heterostructure Design of MnO_2_ for High‐Performance Aqueous Zinc‐Ion Batteries,” Energy & Environmental Science 17, no. 10 (2024): 3629–3640, 10.1039/D4EE00341A.

[8] Y. Chen , C. Lin , X. Chen , et al., “Modulating the Structure of Interlayer/Layer Matrix on δ‐MnO_2_ via Cerium Doping‐Engineering toward High‐Performance Aqueous Zinc Ion Batteries,” Advanced Energy Materials 14 (2024): 2304303.

[9] T. Zhang , T. Li , Y. Shen , et al., “Enhanced Kinetics and Stability of Zn‐MnO_2_ Batteries with a Multifunctional TiO_2_ Coating,” Advanced Materials 37 (2025): 2505082. 10.1002/adma.202505082 40492895

[10] M. Yang , J. Zhu , J. Lin , et al., “A Guest Cation Screening Principle for Enabling Customized Cathode/Electrolyte Interface Chemistry and Self‐Enhanced Aqueous Zinc‐Ion Batteries,” Angewandte Chemie International Edition 64 (2025): 202510893. 10.1002/anie.202510893 40637380

[11] T. Li , N. Zhang , B. Liu , et al., “Unlocking the Critical Role of Cations Doping in MnO_2_ Cathode With Enhanced Reaction Kinetics for Aqueous Zinc Ion Batteries,” Advanced Functional Materials 35 (2025): 2423755.

[12] J. Liang , Y. Zhao , L. Ren , et al., “Dual Anions Doping Enhanced Conductivity and Stability of Layered δ‐MnO2 Cathode for Aqueous Zinc‐Ion Battery,” Advanced Functional Materials 35 (2025): 2501135.

[13] S. Wang , Z. Yuan , X. Zhang , et al., “Non‐Metal Ion Co‐Insertion Chemistry in Aqueous Zn/MnO_2_ Batteries,” Angewandte Chemie International Edition 60, no. 13 (2021): 7056–7060, 10.1002/anie.202017098. 33443304

[14] Y. Xu , G. Zhang , X. Wang , et al., “Efficient Modulation d/p‐Band Center Proximity in Birnessite‐Type MnO_2_ by Cation/Anion Co‐Doping for Enhanced Dual‐Ion Storage,” Advanced Functional Materials 35 (2025): 2500137.

[15] X. Li , J. Wang , H. Xue , et al., “Tuning α‐MnOOH Formation via Atomic‐Level Fe Introduction for Superior OER Performance,” Advanced Functional Materials 35 (2025): 2503360.

[16] T. Wang , J. Jin , X. Zhao , X. Qu , L. Jiao , and Y. Liu , “Unexpected Elevated Working Voltage by Na^+^/Vacancy Ordering and Stabilized Sodium‐Ion Storage by Transition‐Metal Honeycomb Ordering,” Angewandte Chemie International Edition 63 (2024): 202409152. 10.1002/anie.202409152 38923635

[17] J. Yang , C. Li , Q. Zhang , et al., “A Memristive Hyperchaotic Oscillator With Complete Control and its Application in the Electrolysis of Manganese,” Chaos, Solitons & Fractals 183 (2024): 114832.

[18] J. Yang , C. Li , Q. Zhang , et al., “Robust, Self‐Healing AIE Fluorescent Supramolecular Elastomers for Smart Anti‐Counterfeiting,” Chemical Engineering Science 293 (2024): 120030.

[19] J. Yang , Z. Wu , C. Li , et al., “Enhancing Manganese Electrodeposition Efficiency at High Current Densities Utilizing A Novel Variable Gradient Chaotic Current Technique,” Journal of Cleaner Production 503 (2025): 145373.

[20] J. Yang , C. Li , Q. Zhang , et al., “Constructing Chaotic Pulse Electrodeposition of Metallic Manganese via Complete Control of a Memristive Hyperchaotic System,” Chemical Engineering Journal 503 (2025): 157582.

[21] C. Li , J. C. Sprott , X. Zhang , L. Chai , and Z. Liu , “Constructing Conditional Symmetry in Symmetric Chaotic Systems,” Chaos, Solitons & Fractals 155 (2022): 111723.

[22] X. Zhang , C. Li , K. Huang , Z. Liu , and Y. Yang , “A Chaotic Oscillator with Three Independent Offset Boosters and Its Simplified Circuit Implementation,” IEEE Trans Circuits Syst II Express Briefs 71 (2024): 51–55.

[23] K. Huang , C. Li , X. Zhang , I. Moroz , and Z. Liu , “Constructing a Memristive Chaotic Oscillator With 2‐D Offset Boosting,” IEEE Transactions on Computer‐Aided Design of Integrated Circuits and Systems 44, no. 1 (2025): 294–303, 10.1109/TCAD.2024.3434478.

[24] T. M. Kamsma , R. van Roij , and C. Spitoni , “A Simple Mathematical Theory for Simple Volatile Memristors and Their Spiking Circuits,” Chaos, Solitons & Fractals 186 (2024): 115320.

[25] Z. Wu , J. Yang , S. Qin , et al., “Modulating MnO2 Crystal Structure and Electrochemical Performance Via Suspension Stirring Electrolysis,” Chemical Engineering Journal 503 (2025): 158614.

[26] C. Li and J. C. Sprott , “Variable‐Boostable Chaotic Flows,” Optik 127, no. 22 (2016): 10389–10398, 10.1016/j.ijleo.2016.08.046.

[27] X. Zhang , C. Li , T. Lei , H. Fu , and Z. Liu , “Offset Boosting in a Memristive Hyperchaotic System,” Physica Scripta 99 (2023): 015247.

[28] X. Li , D. He , Q. Zhou , et al., “Deciphering Anomalous Zinc Ion Storage in Intermediate‐State MnO_2_ During Layer‐to‐Tunnel Structural Transition,” Energy & Environmental Science 17, no. 23 (2024): 9195–9204, 10.1039/D4EE03293D.

[29] S. Wang , K. Ji , J. Yang , et al., “Spin‐Polarization‐Regulated Orbital Potential in Interfacially Engineered α‐MnO_2_ for Ultra‐Stable High‐Rate Zinc‐Ion Batteries,” Nano Energy 146 (2025): 111561.

[30] C. Julien , “Raman Spectra of Birnessite Manganese Dioxides,” Solid State Ionics 159, no. 3‐4 (2003): 345–356, 10.1016/S0167-2738(03)00035-3.

[31] C. M. Julien , M. Massot , and C. Poinsignon , “Lattice Vibrations of Manganese Oxides,” Spectrochimica Acta Part A: Molecular and Biomolecular Spectroscopy 60, no. 3 (2004): 689–700, 10.1016/S1386-1425(03)00279-8. 14747095

[32] S. Wang , X. Guo , K. Huang , et al., “Cooperative Jahn‐Teller Effect and Engineered Long‐Range Strain in Manganese Oxide/Graphene Superlattice for Aqueous Zinc‐Ion Batteries,” Nature Communications 16 (2025): 5191. 10.1038/s41467-025-60558-y PMC12137938 40467665

[33] Q. Li , M. Xu , S. Wei , et al., “Zn^2+^‐Blocking Effects of a Proton‐Rich Polyaniline Layer Enable Ah‐Level Zn–MnO_2_ Batteries,” Energy & Environmental Science 18, no. 16 (2025): 7939–7949, 10.1039/D5EE02213D.

[34] Y. Yuan , K. He , B. W. Byles , et al., “Deciphering the Atomic Patterns Leading to MnO_2_ Polymorphism,” Chemistry 5, no. 7 (2019): 1793–1805, 10.1016/j.chempr.2019.03.021.

[35] Y. Yuan , W. Yao , B. W. Byles , et al., “Revealing the Atomic Structures of Exposed Lateral Surfaces for Polymorphic Manganese Dioxide Nanowires,” Small Structure 2 (2020): 2000091.

[36] Y. Yuan , C. Liu , B. W. Byles , et al., “Ordering Heterogeneity of [MnO_6_] Octahedra in Tunnel‐Structured MnO_2_ and Its Influence on Ion Storage,” Joule 3, no. 2 (2019): 471–484, 10.1016/j.joule.2018.10.026.

[37] Y. Wang , H. Zhou , S. Wei , et al., “Boosting Ion Transport in Manganese Dioxide Cathodes Through Electronically Tuned Molecular Intercalants,” Journal of the American Chemical Society 147, no. 45 (2025): 41297–41307, 10.1021/jacs.5c08301. 41164856

[38] Q. Zhang , J. Zhao , X. Chen , et al., “Unveiling the Energy Storage Mechanism of MnO_2_ Polymorphs for Zinc–Manganese Dioxide Batteries,” Advanced Functional Materials 34 (2024): 2306652.

[39] L. Wu , Z. Li , Y. Xiang , et al., “Unraveling the Charge Storage Mechanism of β‐MnO_2_ in Aqueous Zinc Electrolytes,” ACS Energy Letters 9, no. 12 (2024): 5801–5809, 10.1021/acsenergylett.4c02559.

[40] C. Cheng , S. Bian , Y. You , et al., “Al Pinning Effect in Birnessite for High‐Performance Ammonium‐Ion Storage,” Advanced Materials 38 (2025): 12356. 10.1002/adma.202512356 41039773

[41] H. Yang , W. Zhou , D. Chen , et al., “The Origin of Capacity Fluctuation and Rescue of Dead Mn‐Based Zn–Ion Batteries: A Mn‐Based Competitive Capacity Evolution Protocol,” Energy & Environmental Science 15, no. 3 (2022): 1106–1118, 10.1039/D1EE03547A.

[42] X. Xue , Z. Liu , S. Chandrasekaran , et al., “Interface‐Controlled Redox Chemistry in Aqueous Mn^2+^/MnO_2_ Batteries,” Advanced Materials 37 (2025): 2419505. 10.1002/adma.202419505 40259491

[43] J. Zhao , X. Ge , A. Kumar , et al., “Regulating the Coordination Environment of Single‐Atom Catalysts for High‐Performance Lithium‐Sulfur Batteries,” Energy Storage Materials 80 (2025): 104085.

[44] H. Yang , T. Zhang , D. Chen , et al., “Protocol in Evaluating Capacity of Zn–Mn Aqueous Batteries: A Clue of pH,” Advanced Materials 35, no. 24 (2023): 2300053, 10.1002/adma.202300053. 37060108

[45] S. Wang , K. Ji , S. Yao , et al., “Fast‐Charging Aqueous Zinc Batteries Enabled by Enhanced O–H Bond Cleavage via d‐p Spin Exchange Interactions,” ACS Energy Letters 10, no. 6 (2025): 2986–2996, 10.1021/acsenergylett.5c00836.

[46] J. Li , X. Yang , J. Wang , et al., “Highly Efficient Mn^2+^ Deposition Induced By H‐Vacancies of NiMn‐LDH Nanosheets for Durable Zinc Ion Batteries,” Energy Storage Materials 74 (2025): 103887.

[47] W. Fan , S. Tian , L. Qin , et al., “Inner‐Sphere Electron Transfer Enabling Highly Reversible Mn^2+^/MnO_2_ Conversion Toward Energy‐Dense Electrolytic Zinc–Manganese Batteries,” Journal of the American Chemical Society 147, no. 22 (2025): 18694–18703, 10.1021/jacs.5c01648. 40397793

[48] Z. Cui , C. Liu , F. Wang , and A. Manthiram , “Navigating Thermal Stability Intricacies of High‐Nickel Cathodes for High‐Energy Lithium Batteries,” Nature Energy 10, no. 4 (2025): 490–501, 10.1038/s41560-025-01731-x.

[49] Z. Liu , M. Qin , B. Fu , M. Li , S. Liang , and G. Fang , “Effective Proton Conduction in Quasi‐Solid Zinc‐Manganese Batteries via Constructing Highly Connected Transfer Pathways,” Angewandte Chemie International Edition 64 (2024): 202417049. 10.1002/anie.202417049 39532684

[50] A.‐C. Huang , Z.‐P. Li , Y.‐C. Liu , et al., “Thermal Hazard Analysis of the Exothermic Reaction of Chlorosulfonic Acid and Organic Compounds,” Journal of Loss Prevention in the Process Industries 72 (2021): 104561.

[51] A.‐C. Huang , C.‐F. Huang , Y. Tang , Z.‐X. Xing , and J.‐C. Jiang , “Thermal Hazard Assessment of Methyl Tert‐Butyl Ether and Its Mixture With Methanol Using DSC and Advanced Thermokinetics,” Journal of Loss Prevention in the Process Industries 69 (2021): 104374.

[52] A.‐C. Huang , C.‐F. Huang , Z.‐X. Xing , J.‐C. Jiang , and C.‐M. Shu , “Thermal Hazard Assessment of the Thermal Stability of Acne Cosmeceutical Therapy Using Advanced Calorimetry Technology,” Process Safety and Environmental Protection 131 (2019): 197–204, 10.1016/j.psep.2019.09.016.

[53] Z.‐H. Wu , Y. Wu , Y. Tang , J.‐C. Jiang , and A.‐C. Huang , “Evaluation of Composite Flame‐Retardant Electrolyte Additives Improvement on the Safety Performance of Lithium‐Ion Batteries,” Process Safety and Environmental Protection 169 (2023): 285–292, 10.1016/j.psep.2022.11.035.

[54] C.‐Z. Zhang , J.‐C. Jiang , A.‐C. Huang , et al., “A Novel Multifunctional Additive Strategy Improves the Cycling Stability and Thermal Stability of SiO/C Anode Li‐Ion batteries,” Process Safety and Environmental Protection 164 (2022): 555–565, 10.1016/j.psep.2022.06.046.

[55] Y.‐P. Yang , J.‐C. Jiang , A.‐C. Huang , et al., “3‐(Trifluoromethyl)Benzoylacetonitrile: A Multi‐Functional Safe Electrolyte Additive for LiNi_0.8_Co_0.1_Mn_0.1_O_2_ Cathode of High Voltage Lithium‐Ion Battery,” Process Safety and Environmental Protection 160 (2022): 80–90, 10.1016/j.psep.2022.02.018.

[56] J. Li , R. Shi , J. Wang , et al., “Degradation Mechanisms of Electrodes Promotes Direct Regeneration of Spent Li‐Ion Batteries: A Review,” Advanced Materials 37 (2024): 2313273. 10.1002/adma.202313273 38533901

[57] F. Yang , X. Chen , G. Qu , et al., “Electrode Separation Via Water Electrolysis for Sustainable Battery Recycling,” Nature Sustainability 8, no. 5 (2025): 520–529, 10.1038/s41893-025-01539-3.

[58] Y. Guo , L. Shi , X. Shi , et al., “Scalable Metal–Organic Framework‐Based Electrodes for Efficient Alkaline Water Electrolysis,” Nature Chemical Engineering 2, no. 8 (2025): 474–483, 10.1038/s44286-025-00262-2.

[59] U. S. Energy information administration , https://www.eia.gov/electricity/data.php.

[60] International energy agency . https://www.iea.org/reports/electricity‐2025/prices#abstract.

