# Supplementary material for: Programmable Chaotic Suspension Electrolysis for Scalable Manufacturing of Vacancy‐Tunable Electrolytic MnO2
Source: Adv Sci (Weinh). 2026 Jul 23:e76766. Online ahead of print. doi: 10.1002/advs.76766 (PMC13393285; doi:10.1002/advs.76766)
Supplement: Supplementary file 1 — Supporting File: advs76766‐sup‐0001‐SuppMat.docx. [file ADVS-9999-e76766-s001.docx]

**Supporting Information for**

**Programmable Chaotic Suspension Electrolysis for Scalable Manufacturing of Vacancy-Tunable Electrolytic MnO_2_**

**Zhihao Wu^1^, Jie Yang^1^, Yidan Fan^1^, Wentao Li^1^, Jiaxing Li^1^, Jing Guo^1^, Chunbiao Li^2^, Lei Shi^3^, Jiaoyuan Zhang^4^, Xingna Cai^1^, Zukang Chen^1^, Qian Zhang^1^, Jun Du^1^,** **Changyuan Tao^1^,** **Qizhi Chen^3^, Guocan Zheng^1^, and Zuohua Liu^1^***

^1^School of Chemistry and Chemical Engineering, Chongqing University, Chongqing, China.

^2^School of Artificial Intelligence, Nanjing University of Information Science and Technology, Nanjing, China.

^3^Guangxi Huiyuan Manganese Industry Co., Ltd., Laibin, China.

^4^School of Chemistry and Chemical Engineering, Guangxi University, Nanning, China.

*e-mail: liuzuohua@cqu.edu.cn

**Table of Contents**

1. Materials and Methods (P1–3)

2. Notes S1–S4 (P4–9)

3. Figures S1–S38 (P10–47)

4. Tables S1–S9 (P48–53)

**Materials and Methods**

**Chemicals**

Manganese sulfate monohydrate (MnSO_4_·H_2_O, 99%), zinc sulfate heptahydrate (ZnSO_4_·7H_2_O, 99.5%), potassium permanganate (KMnO_4_, 99.5%), ammonium persulfate ((NH_4_)_2_S_2_O_8_, 98%), and ammonium chloride (NH_4_Cl, 99.5%) were purchased from Aladdin. Concentrated sulfuric acid (H_2_SO_4_, 98%) was obtained from Chuandong Chemical. N-Methyl-2-pyrrolidone (NMP), acetylene black, and polyvinylidene fluoride (PVDF) were sourced from Shenzhen Kejing.

**Electrolysis process**

The electrolyte for preparing D-M, S-M, and CS-M1 to CS-M4 contained 0.45 M Mn^2+^ and 1.0 M H^+^, with a current density of 60 A m^−2^. The electrolysis temperature was 80 °C for S-M and CS-M1 to CS-M4, and 95 °C for D-M. A graphite plate served as the cathode, and a coated titanium plate was used as the anode. The effective electrode areas for standard lab-scale, scaled-up lab-scale, and industrial pilot-scale electrolysis were 5 × 5 × 2 cm^2^, 15 × 12 × 2 cm^2^, and 55 × 40 × 2 cm^2^, respectively. Suspension particles were sieved through a 500-mesh screen and dispersed in the electrolyte via ultrasonication for 20 min. The chaotic system dynamics were solved using the MATLAB ode45 solver; circuit simulations were performed using Multisim; and the hardware circuit was implemented on a printed circuit board (PCB).

**Structural characterization**

XRD (Panalytical EMPYREAN, Cu Kα radiation) was used for phase and crystal structure analysis, with Rietveld refinement performed using the GSAS software. Morphology and microstructure were characterized by SEM (Hitachi Regulus 8230), AFM (Oxford Cypher S), and HAADF-STEM (JEOL ARM200F). Raman spectroscopy (HORIBA LabRAM HR Evolution, 532 nm wavelength) was employed to analyze the stretching vibration modes of chemical bonds in the [MnO_6_] octahedra. EELS, XPS (Thermo Fisher ESCALAB 250Xi), and EPR (Bruker EMXplus) were used to analyze chemical composition, valence states, and oxygen vacancies. Mn K-edge XAS data were collected at the 1W2B beamline of the Beijing Synchrotron Radiation Facility (BSRF).

**Battery assembly and testing**

The cathode slurry was prepared by mixing active material, acetylene black, and PVDF in a mass ratio of 8:1:1 in NMP, coated onto a titanium mesh, and vacuum-dried at 80 °C for 10 h. The anode was a 15 μm thick zinc foil. The electrolyte was an aqueous solution of 2 M ZnSO_4_ and 0.2 M MnSO_4_. For coin cells, the cathode loading was 1–2 mg cm^−2^, the electrode diameter was 16 mm, and 100 μL of electrolyte was added. For pouch cells, the cathode loading was 10 mg cm^−2^, the electrode size was 6 × 7 cm^2^, and 2 mL of electrolyte was added. Commercial alkaline LR6 batteries were assembled in strict accordance with industry standards. All battery tests were performed on a Neware test system (CT-4008Q). GITT measurements were conducted at 0.1 A g^−1^ using a cycle of 5 min pulse and 60 min relaxation.

**Thermal analysis**

Pouch cells after cycling were disassembled, and the cathodes were washed with deionized water, vacuum-dried at 80 °C for 10 h, and the active materials were collected. TG-MS (NETZSCH STA 449 F5 + QMS 403 D) was performed under an argon atmosphere at a *β* of 10 °C min^−1^, with a sample mass of approximately 8 mg. DSC (NETZSCH DSC 404 F3) and TG (Mettler Toledo TGA2) were conducted in air at *β* = 10 °C min^−1^ (single rate) and *β* = 2, 5, 10, 15, and 20 °C min^−1^ (multiple rates), with a sample mass of approximately 8 mg.

**Theoretical calculations**

Spin-polarized DFT calculations were performed using VASP. The projector augmented-wave method was employed, van der Waals interactions were corrected using the DFT-D3 method, and the exchange-correlation functional was treated within the PBE generalized gradient approximation. A plane-wave cutoff energy of 450 eV was used. The convergence criteria for energy and force were set to 10^−4^ eV and 0.02 eV Å^−1^, respectively. A vacuum layer of 15 Å was applied perpendicular to the slab to eliminate periodic interactions.

**Sustainability assessment**

LCA and TEA were conducted using SimaPro software, invoking the ecoinvent 3.10 database. These analyses were grounded in the most recent authorized real-world industrial data, which were systematically collected and integrated based on a specific production line scale. Detailed energy consumption, power load, electricity consumption calculations, and material balance tables are provided in Tables S3–S9 and Note S4.

**Note S1: Construction, Dynamical Analysis, and Multistability Characteristics of the Five-Dimensional Chaotic System**

This study presents the design of a novel five-dimensional chaotic system, which was evolved from the foundational three-dimensional *VB*15 chaotic system.$\begin{aligned} \left\{ \begin{aligned} &\dot{x}=az-yz \\ &\dot{y}=z^{2}-by \\ &\dot{z}=x-z \end{aligned} \right.\#VB15 \end{aligned}$

When the system parameters are set to *a* = 4.0, *b* = 0.4, and the initial conditions are (0.1, 0.1, 0.1), the system exhibits well-defined chaotic dynamics.

The Lyapunov exponents and bifurcation diagrams serve as critical indicators for identifying the dynamical regime of a system. When the system is in a periodic state, its largest Lyapunov exponent remains at zero, and the corresponding bifurcation diagram displays a finite set of discrete points. Conversely, under chaotic conditions, the system exhibits a positive largest Lyapunov exponent, accompanied by bifurcation diagrams characterized by continuous and densely distributed point sets. Furthermore, if the Lyapunov exponents remain consistently positive and stable while a specific parameter varies, it indicates that the parameter does not influence the chaotic nature of the system, thereby reflecting robust chaotic dynamics with respect to that parameter dimension.

To systematically investigate the influence of system parameters on the dynamical behavior, we examined the effects of parameters *a*, *b*, and *c* on both the Lyapunov exponents and bifurcation diagrams. The results demonstrate that parameters *a* and *c* play a significant role in regulating the system's behavior, capable of driving transitions between periodic and chaotic states. For instance, when *c* = 0.9, the largest Lyapunov exponent of the system is zero, and the bifurcation diagram shows a single-point distribution, indicating a period-1 state. In contrast, when *c* decreases to 0.7, the system transitions into a chaotic state (Figures S5a, b, e and f). On the other hand, parameter *b* can modulate the amplitude of the chaotic signal without altering the Lyapunov exponents, indicating that the system maintains stable chaotic characteristics during variations in *b*, thereby demonstrating favorable parameter robustness (Figures S5c and d).

Furthermore, the system exhibits rich multistability, whereby it converges to distinct attractors under different initial conditions. Taking parameter *c* as an example, when *c* = 1.0, the system exhibits coexisting period-1 attractors (Figure S6a), whose basin structures are shown in Figure S6b. At *c* = 0.8, coexisting period-2 attractors emerge (Figure S6c), while at *c* = 0.75, coexisting chaotic attractors are observed (Figure S6e). The basin structures for each of these coexisting states have been fully characterized (Figures S6d and f), further revealing the system's high sensitivity to initial conditions and its inherent symmetric organization.

**Note S2: Thermal Analysis Methodology and Rationale for Kinetic Modeling**

**2.1 Rationale for Sample Mass and Test Atmosphere Selection**

In the DSC tests, a cathode material mass of approximately 8 mg was selected based on the following considerations: an excessive sample mass would lead to rapid pressure buildup within the crucible due to evolved decomposition gases, potentially causing crucible displacement and distortion of the heat flow signal; conversely, an insufficient mass would result in weak signal intensity, poor signal-to-noise ratio, and compromised consistency in data trends across multiple heating rates. To ensure data comparability, TG was performed using an identical sample mass.

Given that aqueous zinc-ion batteries are assembled in air, all DSC and TG measurements were conducted under an air atmosphere. In contrast, a pure argon atmosphere was employed for the TG-MS tests. This choice was made to eliminate interference from atmospheric oxygen, thereby enabling precise detection of the CO_2_ signal generated from the reaction between oxygen (released from Mn–O bond breakage in MnO_2_) and carbon. This approach allows for the quantitative assessment of Mn–O bond dissociation and the thermal stability of the materials.

**2.2 Rationale for Model Selection and Data Suitability**

Although both DSC and TG data can be utilized for kinetic analysis, DSC is susceptible to signal fluctuations in systems with slow or unstable heat release, leading to significant errors in multiple *β* fitting. In contrast, TG modeling, which is inherently based on mass changes, is less affected by heat transfer variations. Consequently, TG analysis maintains superior applicability and reliability for weakly exothermic systems. Therefore, TG data served as the primary basis for kinetic analysis in this study.

**Note S3: Faradaic Efficiency Calculation and Product Purity Analysis Methods**

**3.1 Faradaic Efficiency Calculation**

Following electrolysis, the anode plate was extracted, thoroughly rinsed with deionized water, and dried at 80 °C for 10 h until constant weight was achieved. The mass of deposited MnO_2_ was determined by the mass difference of the plate before and after electrolysis. The Faradaic efficiency (*η*) was subsequently calculated using the following equation:

$$\begin{aligned} \eta=\frac{\Delta M}{\Delta tIE_{{MnO}_{2}}} \end{aligned}$$

where Δ*M*/Δ*t* (g h^−1^) represents the mass deposition rate of MnO_2_, *I* denotes the average current (A), and *E*_MnO2_ is the electrochemical equivalent of MnO_2_ (1.6216 g A^−1^ h^−1^).

The industrial EMD process, refined over years of optimization, inherently achieves high Faradaic efficiency. The measured efficiency slightly exceeding 100% is primarily attributed to the presence of crystalline water in the product (Figure S35). Results from 15 independent replicate experiments confirm that the efficiency consistently resides within the range of 105–110%. These findings demonstrate that the chaotic suspension electrolysis process maintains exceptionally high Faradaic efficiency while simultaneously reducing the operating temperature.

**3.2 MnO_2_ Purity Determination**

The product purity was determined according to standardized industry methods by the collaborating enterprise. The primary procedure consisted of the following steps:

(1) Preparation of a 0.1067 mol L^−1^ Na_2_S_2_O_3_ standard solution and a 0.5% starch indicator solution.

(2) Preparation of four test samples (each containing 0.10 g MnO_2_, 4 g KI, and 50 mL H_2_O) and one blank sample (without MnO_2_).

(3) After allowing the reaction to proceed for 50 min, titration with the Na_2_S_2_O_3_ standard solution was performed until the solution colour faded from pale blue to colourless.

(4) Data from three parallel experiments showing good agreement were selected, and the MnO_2_ purity (*w*) was calculated using the following formula, incorporating the measured moisture content (*W*) of the sample:

$$\begin{aligned} w=\frac{c\times\left( V_{1}-V_{2} \right)\times{10}^{-3}\times86.94}{2\times m\times\left( 1-W \right)}\times100 \end{aligned}$$

where *c* is the concentration of the sodium thiosulfate solution (mol L^−1^), *V*_1_ and *V*_2_ are the titration volumes of the test sample and blank sample, respectively (mL), *m* is the mass of the sample (g), and 86.94 is the molar mass of MnO_2_ (g mol^−1^).

**Note S4: Data Foundation and System Boundaries for Sustainability Assessment**

LCA and TEA presented in this study are based on authorized actual operational data from 2022, provided by a specific manganese producer. The company's total annual production capacity for EMD is approximately 180,000 tonnes. However, as the production process parameters vary across different lines to meet specific client requirements, this study selected the most universally representative conventional EMD production process to ensure consistency and representativeness in the analysis. The annual output of the corresponding production line is 75,000 tonnes. All data presented in Tables S3–S9 were systematically collected, collated, and integrated based on this specific production line scale.


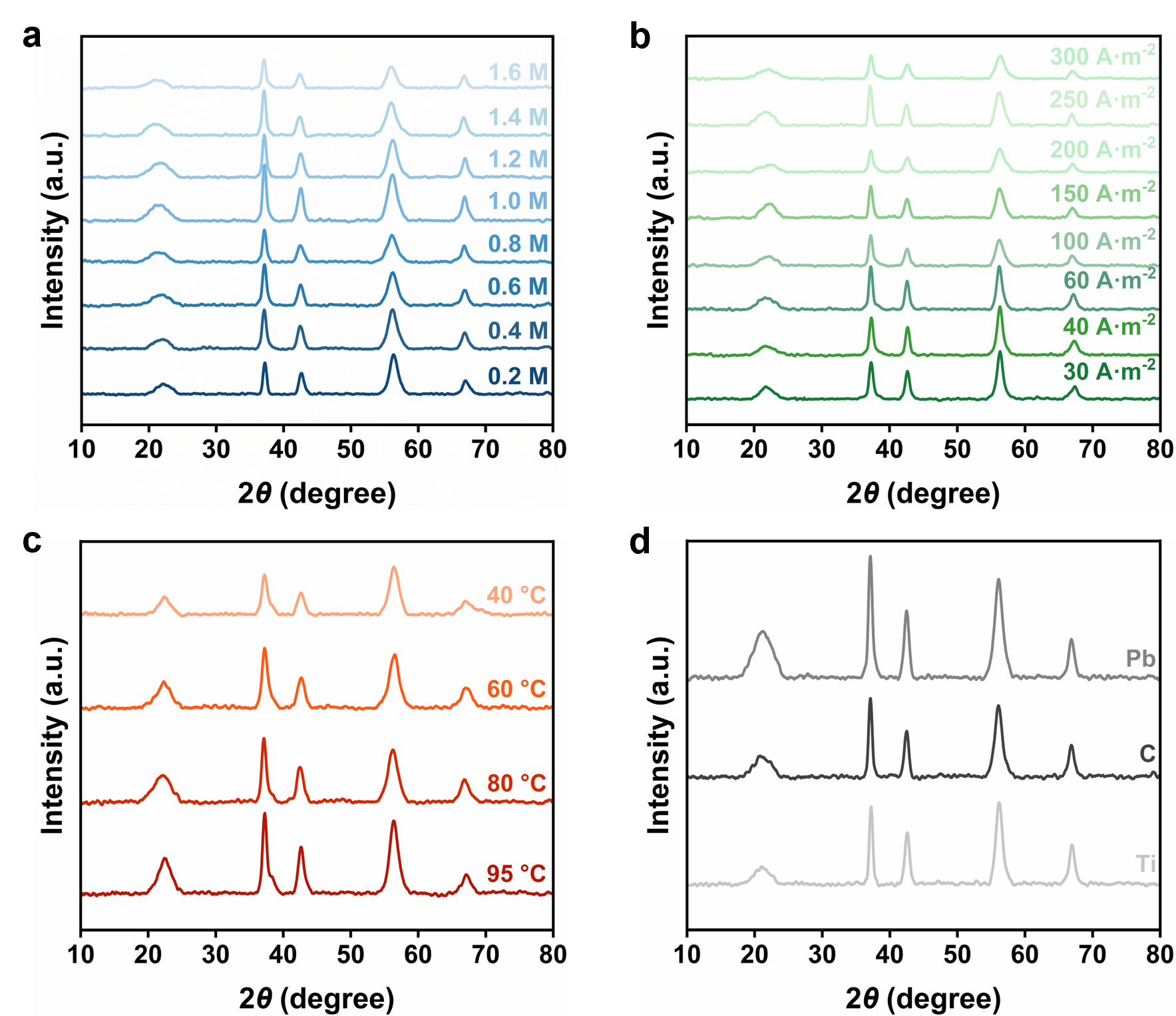


**Figure S1.** XRD patterns of EMD products obtained under different electrolytic conditions. (a) H^+^ concentration, (b) current density, (c) electrolysis temperature, and (d) anode material.


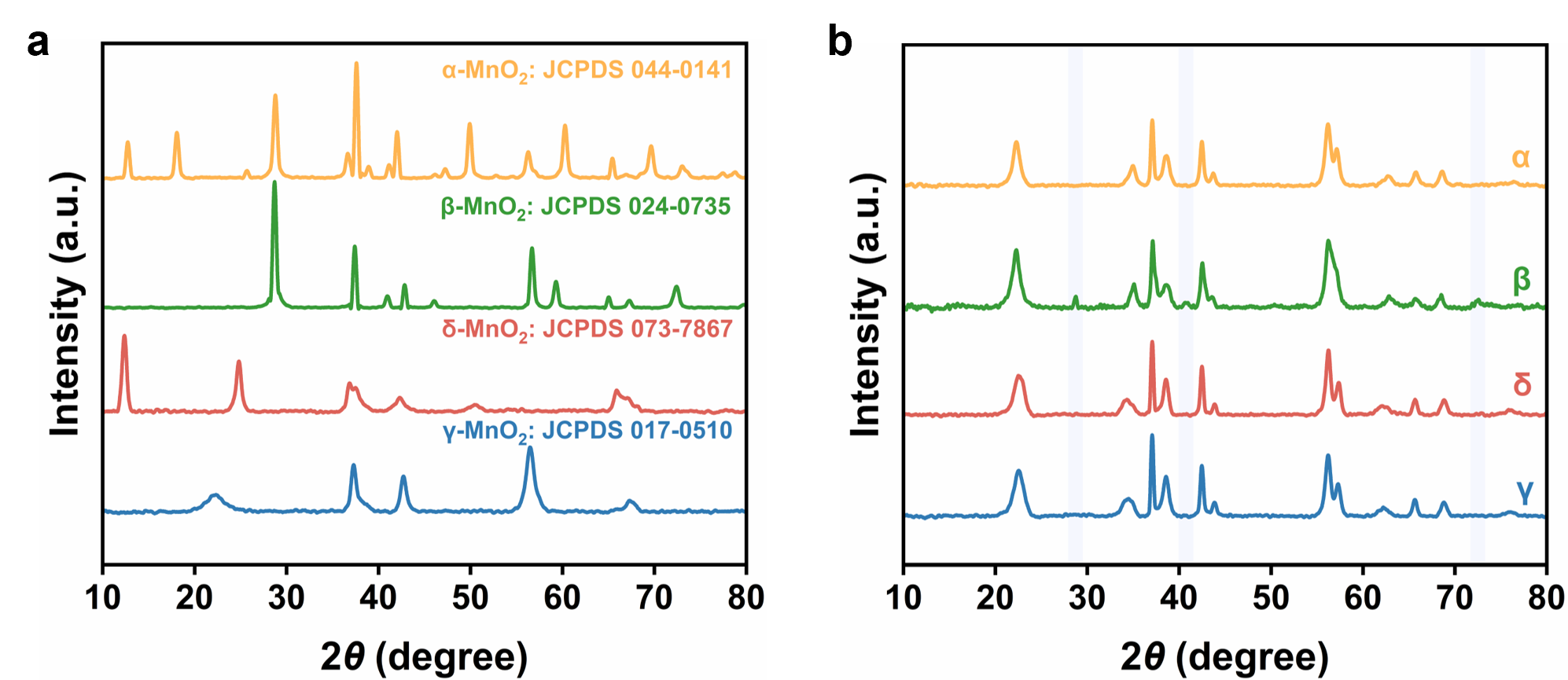


**Figure S2.** Influence of the crystal phase of suspended particles on the structure of S-M. (a) XRD patterns of different MnO_2_ crystal phases. (b) XRD patterns of the resulting S-M products when the corresponding MnO_2_ phases were employed as suspended particles (addition amount: 1.0 g L^−1^).


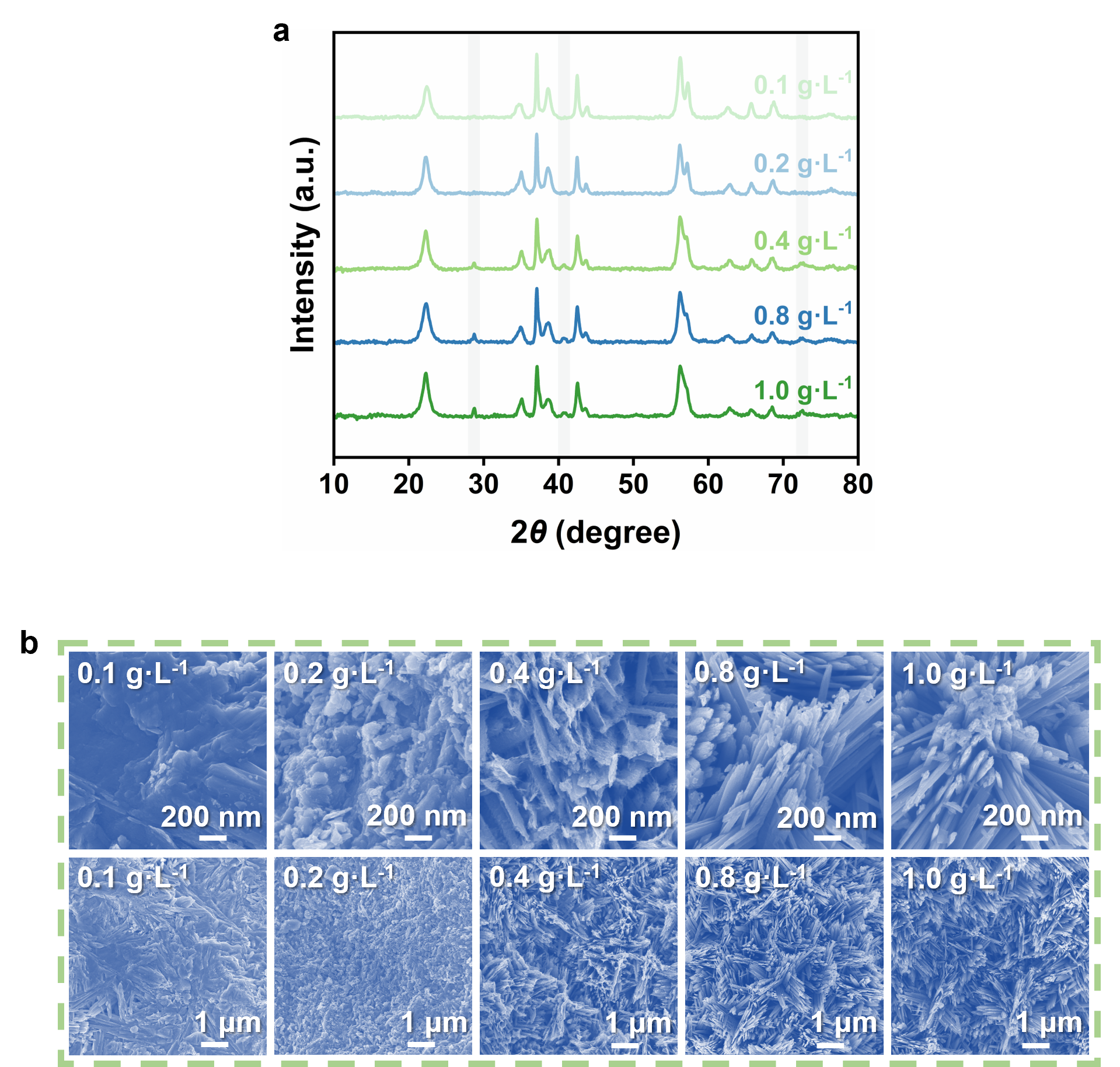


**Figure S3.** Tailoring the structure and morphology of S-M through β-MnO_2_ suspension additive amount. (a) XRD patterns and (b) SEM morphology of S-M obtained with different additive amounts.


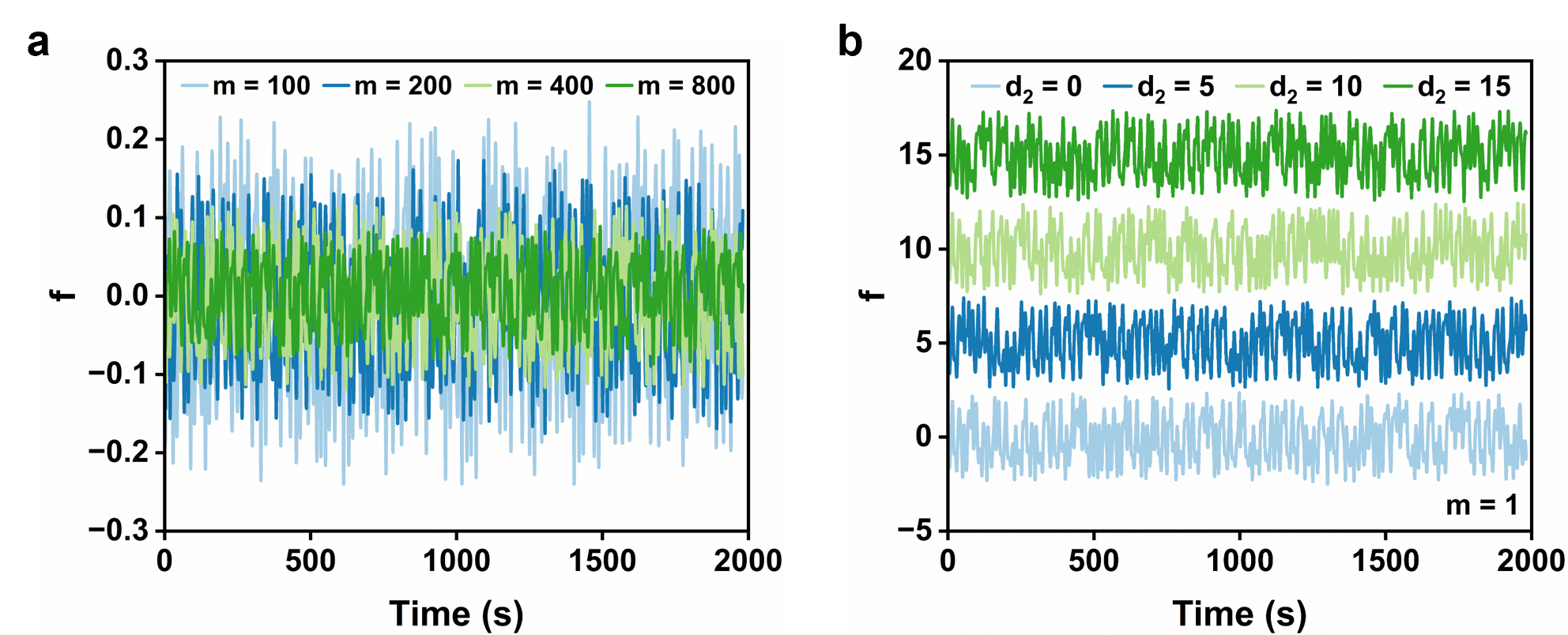


**Figure S4.** Independent control of output signals through parameters in the chaotic circuit. The system demonstrates decoupled tuning capabilities with (a) parameter *m* controlling the signal amplitude and (b) parameter *d*_2_ regulating the signal bias.


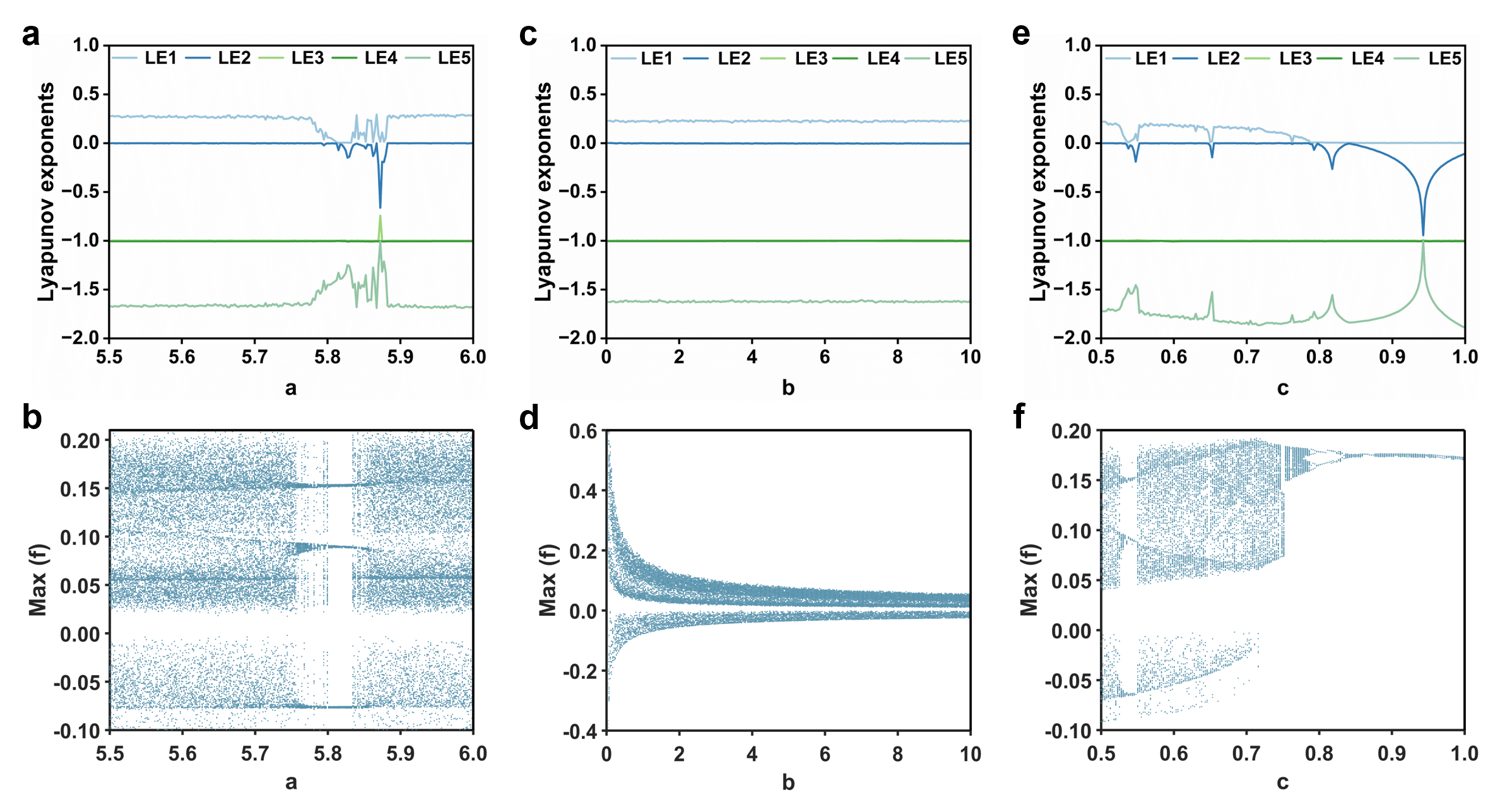


**Figure S5.** Dynamical evolution of the chaotic system under parameter variations. The effects of system parameters on Lyapunov exponent spectra and bifurcation diagrams are respectively illustrated for: (a, b) parameter *a*, (c, d) parameter *b*, and (e, f) parameter *c*.


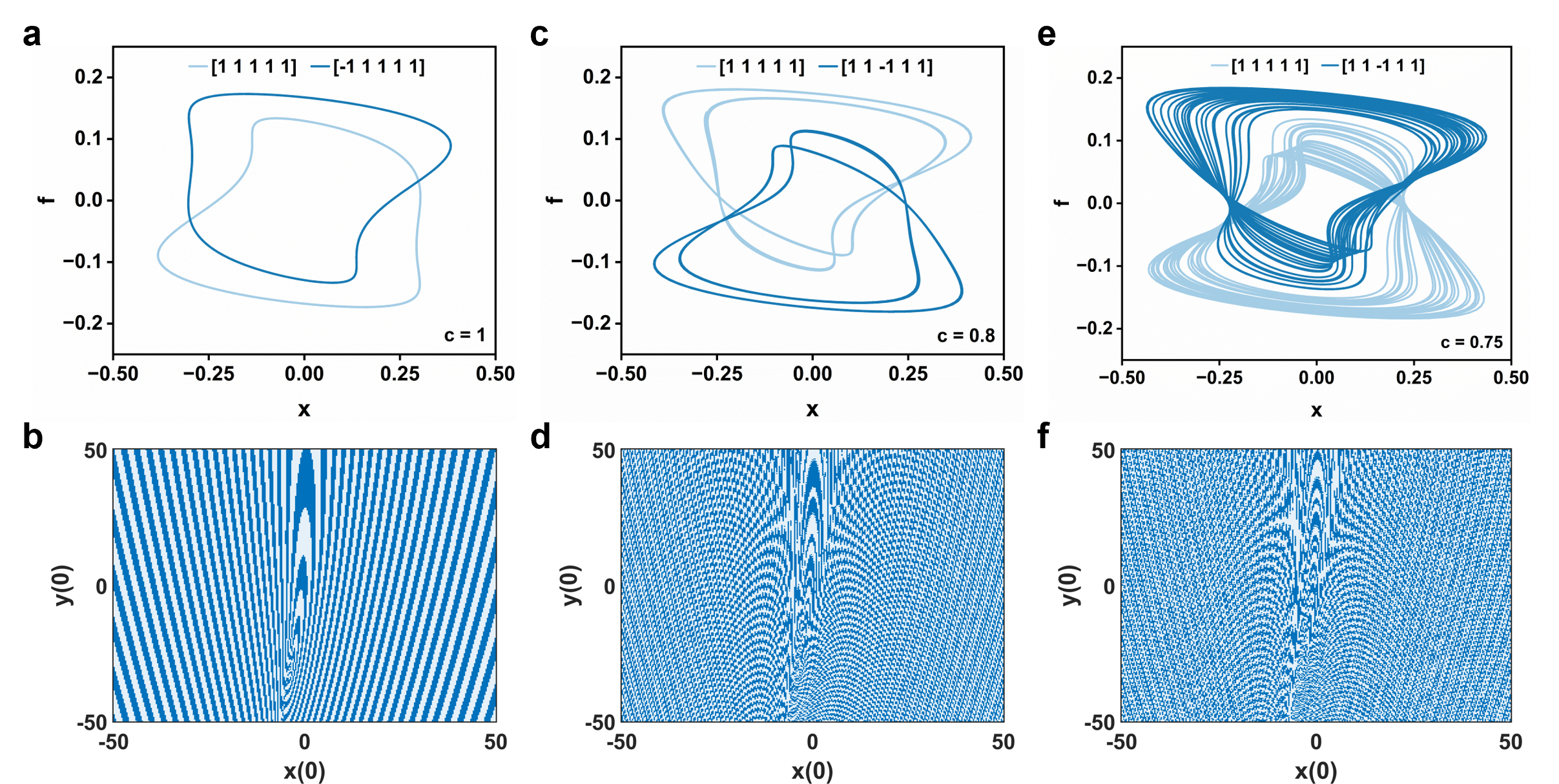


**Figure S6.** Multistability regulated by parameter *c* in the chaotic system. (a, b) Coexisting period-1 attractors and their basin of attraction at *c* = 1; (c, d) coexisting period-2 attractors and corresponding basin at *c* = 0.8; (e, f) coexisting chaotic attractors with their basin structure at *c* = 0.75.


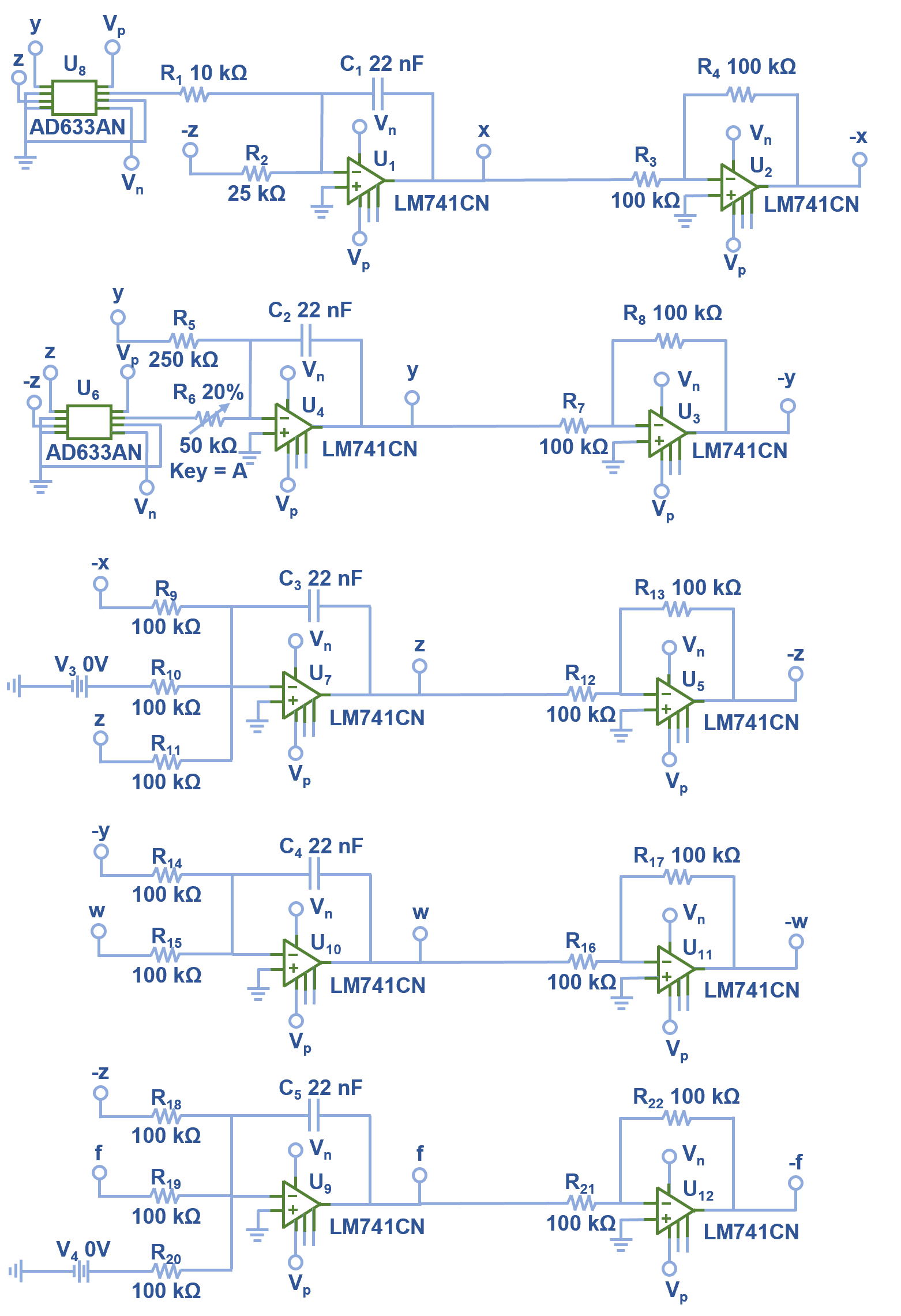


**Figure S7.** Hardware implementation of the chaotic circuit: system architecture and corresponding component parameters.


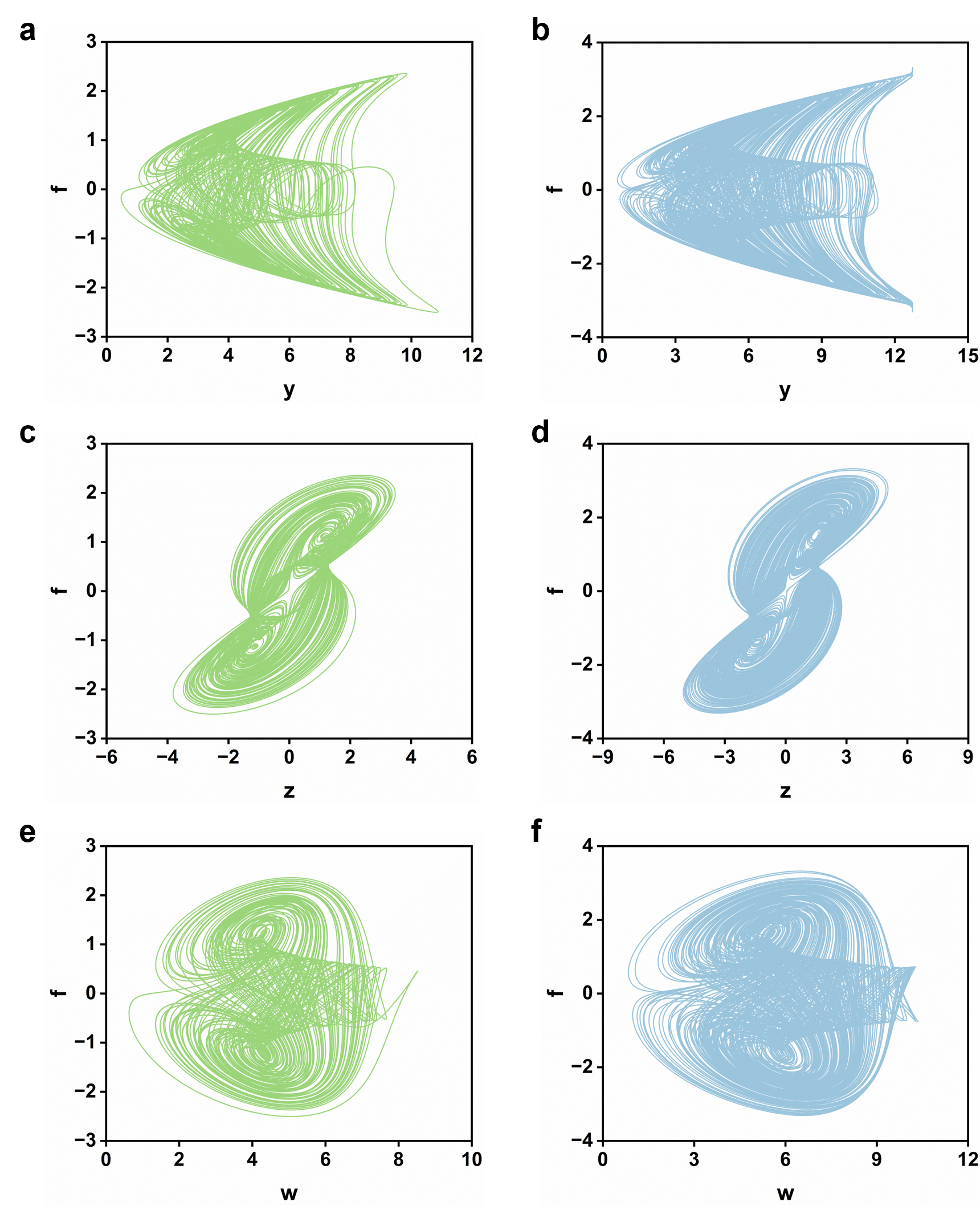


**Figure S8.** Validation of consistency between theoretical design and circuit simulation of the chaotic system. Phase portrait comparisons between theoretical numerical simulations and circuit simulations are shown for signal pairs: (a, b) *y*-*f*; (c, d) *z*-*f*; and (e, f) *w*-*f*.


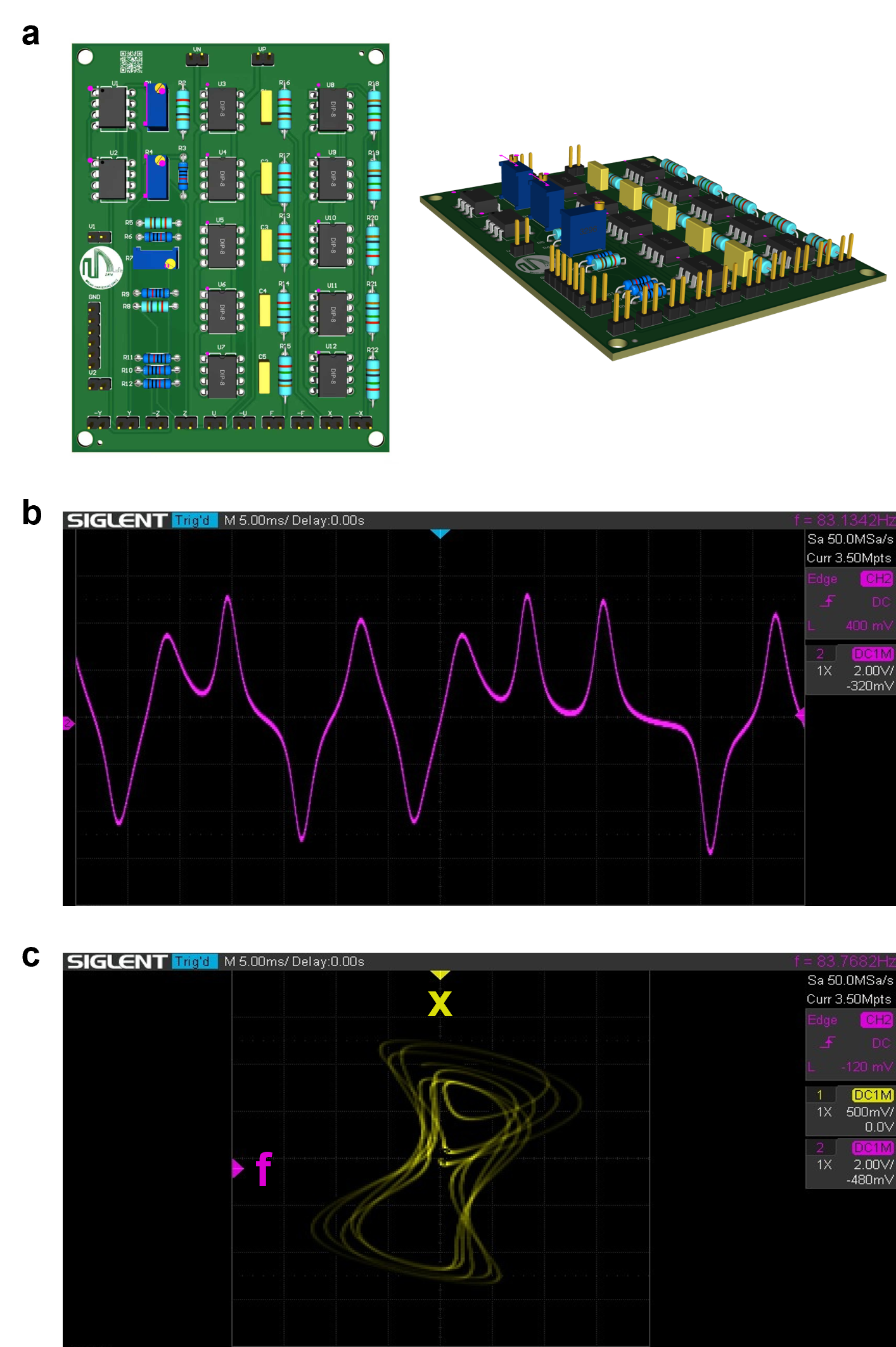


**Figure S9.** Hardware implementation and experimental signal characterization of the chaotic circuit. (a) Three-dimensional physical layout of the implemented circuit; (b) Experimentally measured waveform of chaotic signal *f* captured by oscilloscope; (c) Experimentally obtained *x*-*f* phase portrait.


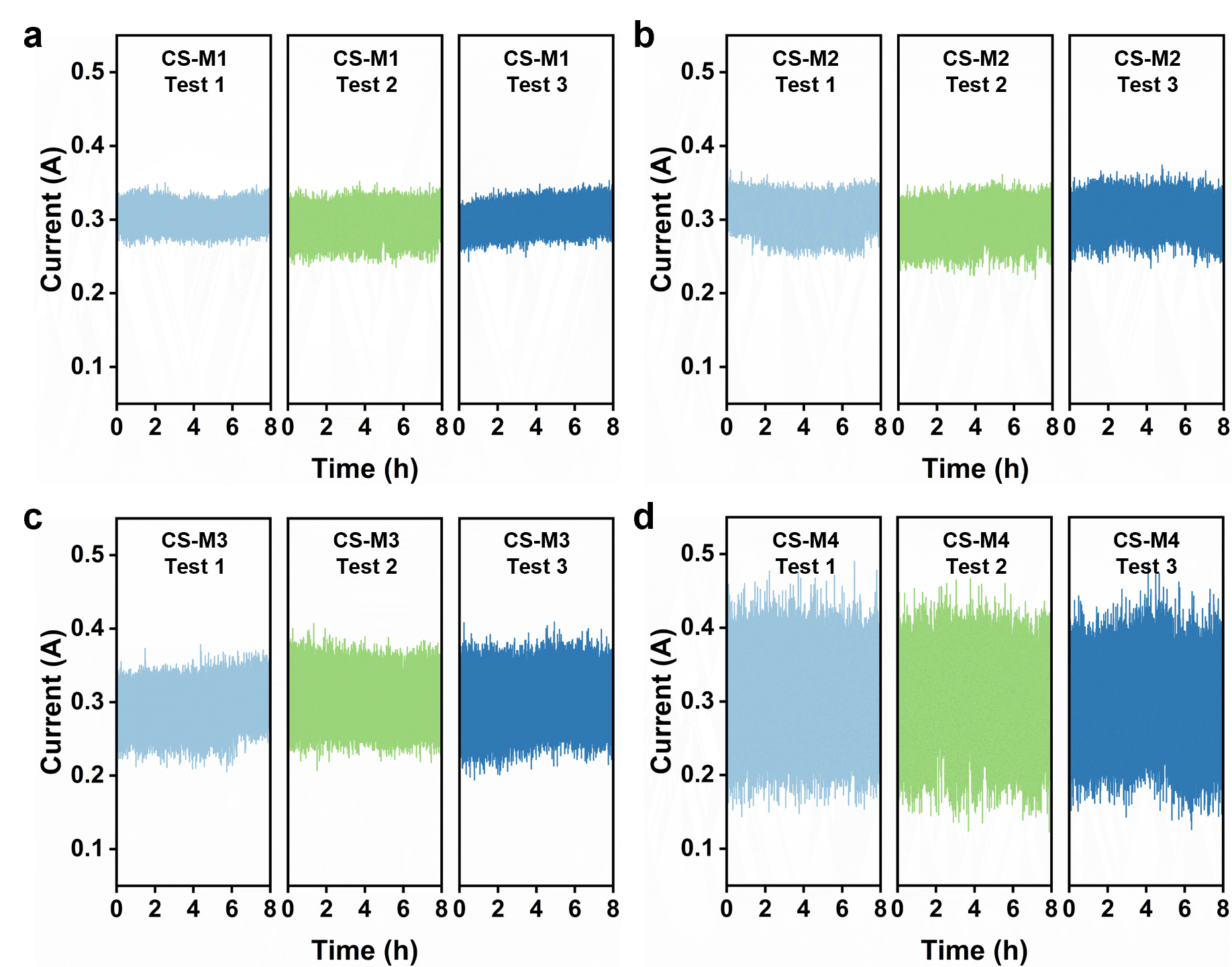


**Figure S10.** Experimental acquisition of chaotic-current signals with varying amplitudes during the CS-M production process. (a) CS-M1, (b) CS-M2, (c) CS-M3, and (d) CS-M4, shown in order of increasing amplitude.

The electrolysis was conducted at a fixed current density of 60 A m^−2^, corresponding to an average current of 0.3 A based on an effective electrode area of 5 × 5 × 2 cm^2^. To ensure process reproducibility and circuit operational stability given the nonlinear nature of the chaotic-current, each amplitude condition was tested with three independent replicates, with each electrolysis lasting 8 h.


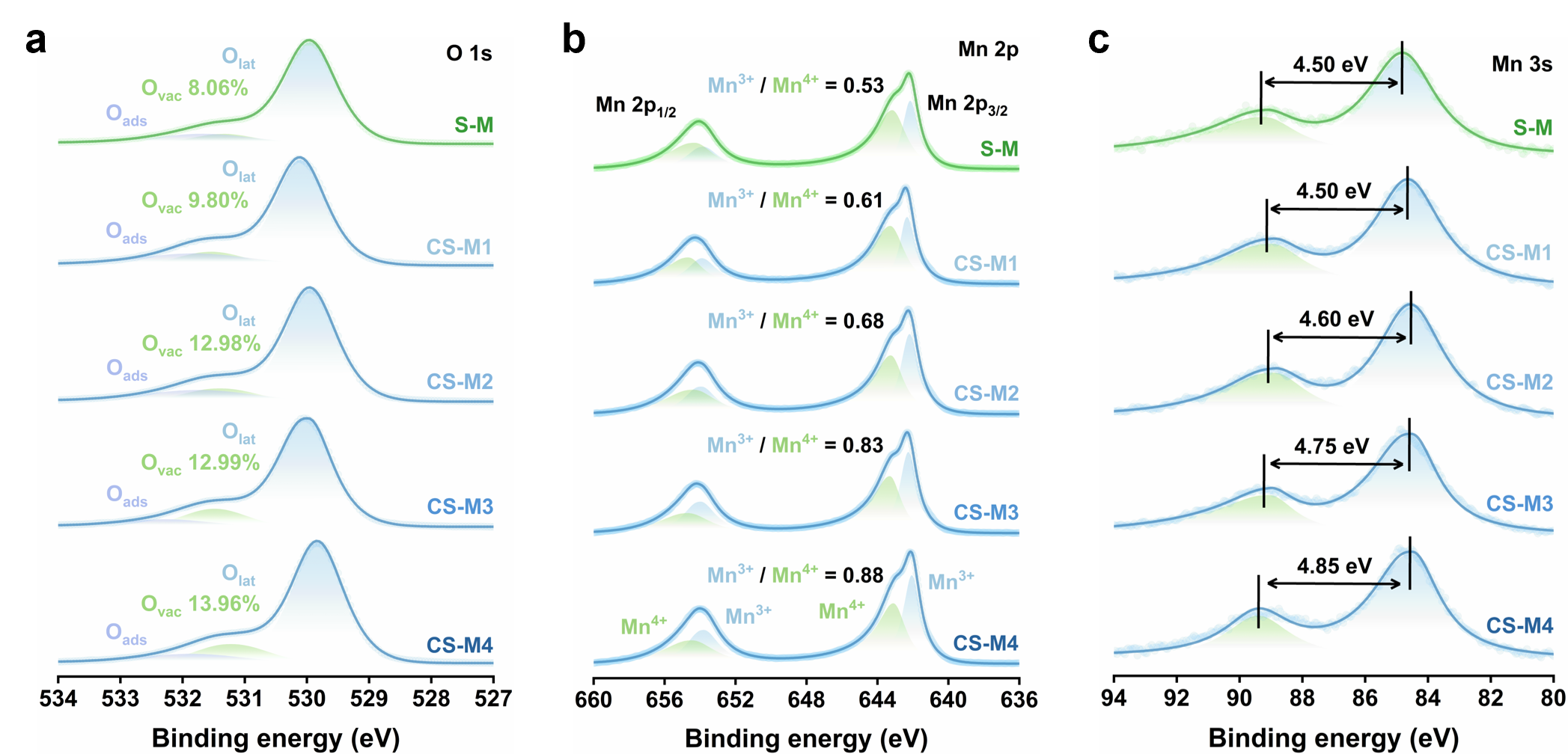


**Figure S11.** XPS analysis of (a) O 1s, (b) Mn 2p, and (c) Mn 3s core levels for S-M, CS-M1, CS-M2, CS-M3, and CS-M4.


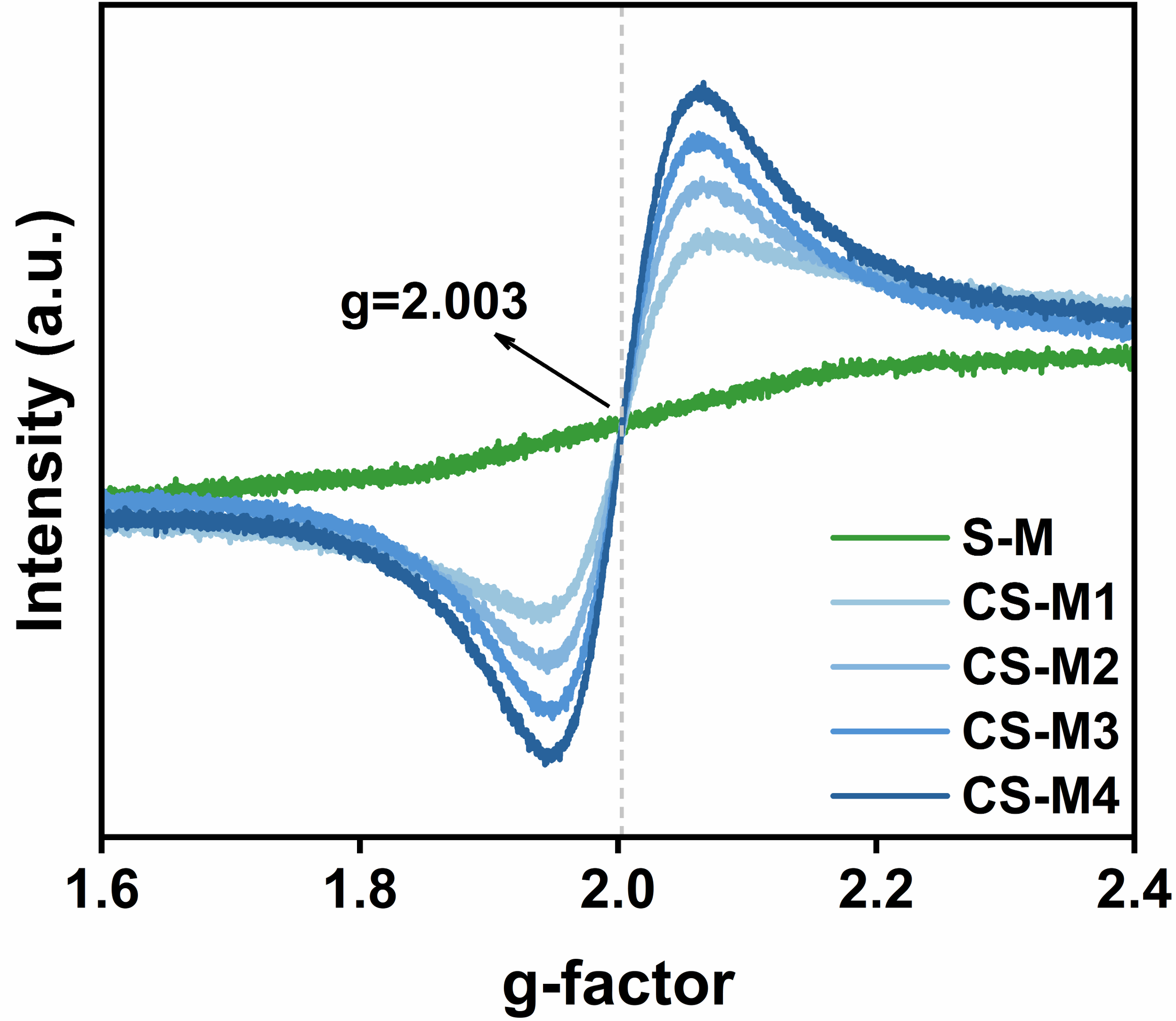


**Figure S12.** EPR spectra of S-M, CS-M1, CS-M2, CS-M3, and CS-M4.


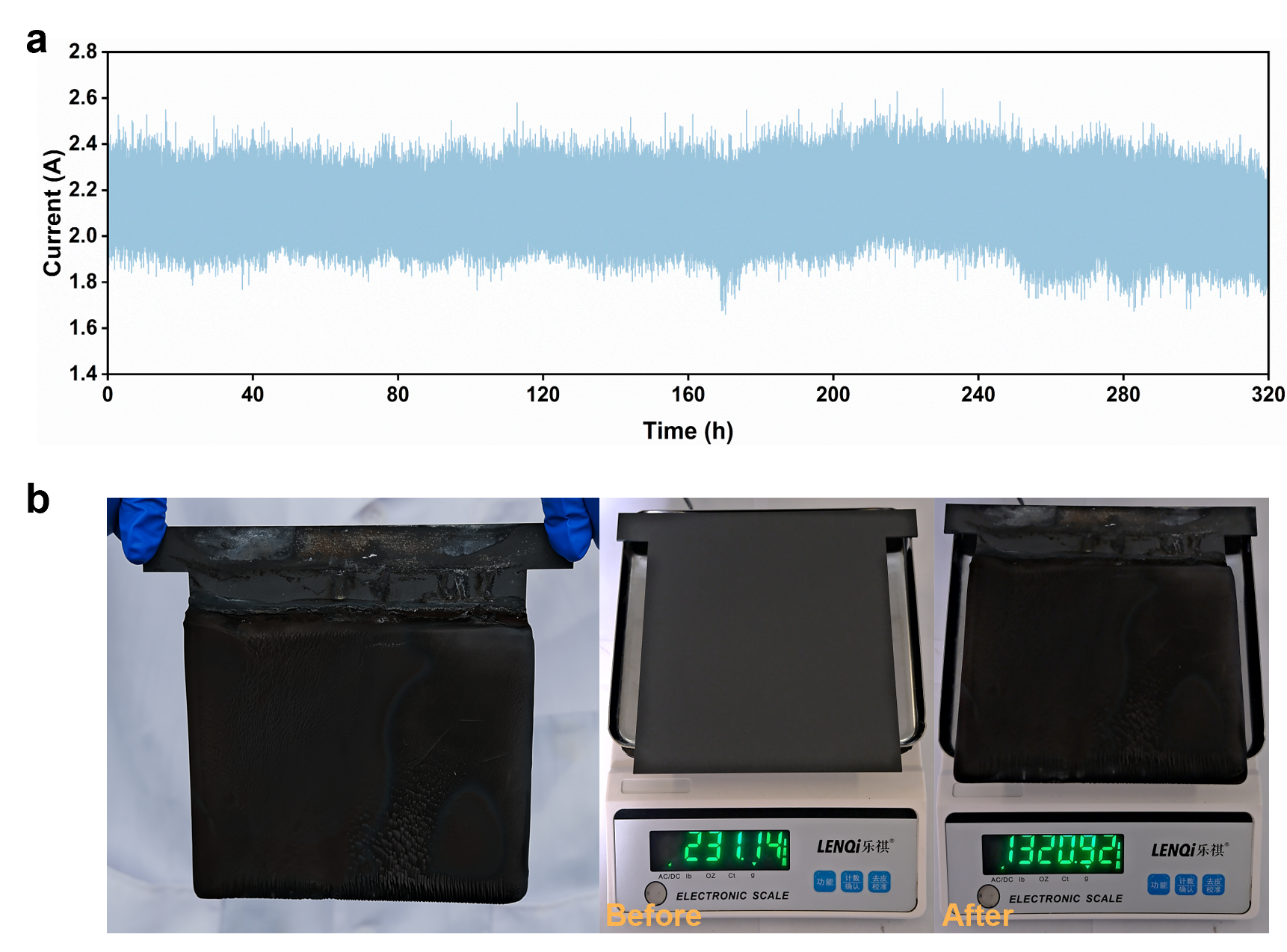


**Figure S13.** (a) Current signal acquisition during laboratory-scale amplification of chaotic suspension electrolysis; (b) morphology of kilogram-scale MnO_2_ deposited on the electrode plate via scaled-up chaotic suspension electrolysis.

The electrolysis was performed at a fixed current density of 60 A m^−2^. With an effective electrode area of 15 × 12 × 2 cm^2^, the average current was maintained at 2.16 A throughout the approximately two-week operation. These signals confirm that the chaotic suspension electrolysis process maintains stable operation under scaled-up conditions.

After two weeks of electrolysis, the deposited plate was cooled to room temperature, washed with deionized water, and dried at 80 °C for 10 h. The measured mass gain of 1089.78 g demonstrates the successful preparation of kilogram-level MnO_2_ on a single plate at laboratory scale.


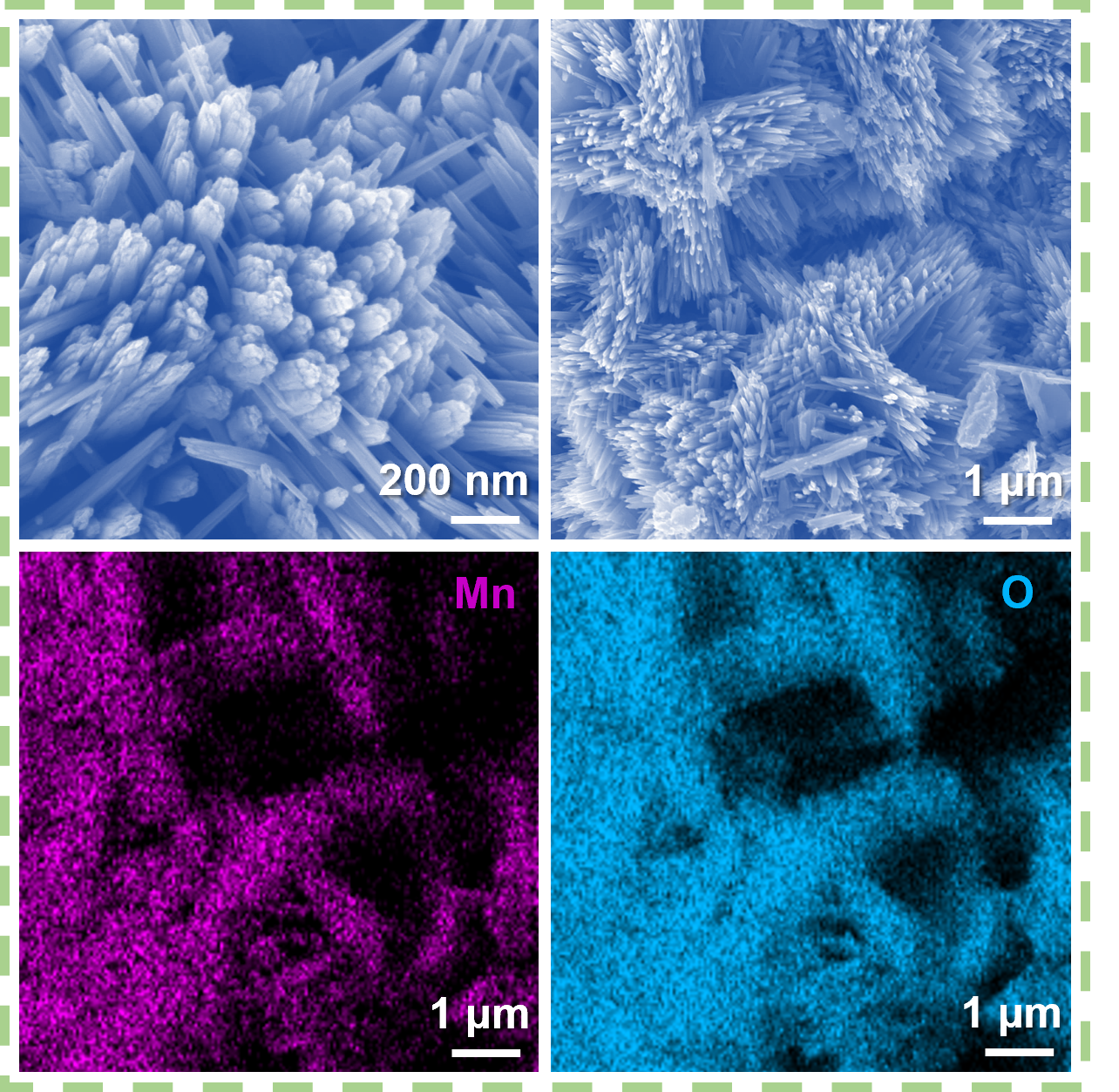


**Figure S14.** SEM morphology and EDS elemental mapping of CS-M4.


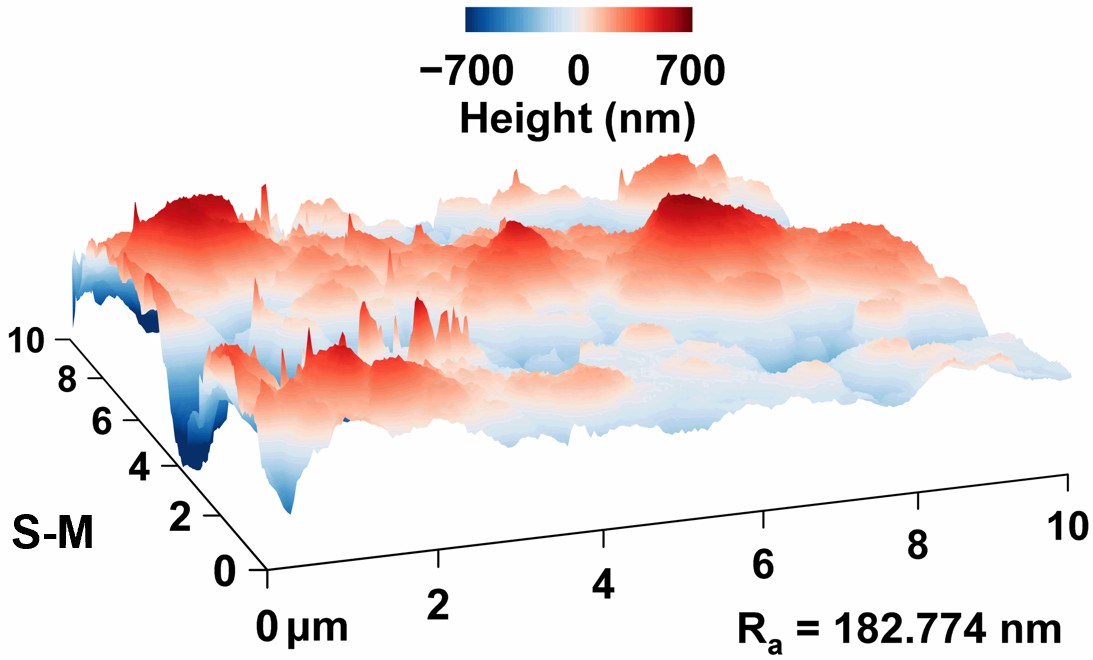


**Figure S15.** Three-dimensional topographic image of S-M obtained by AFM.


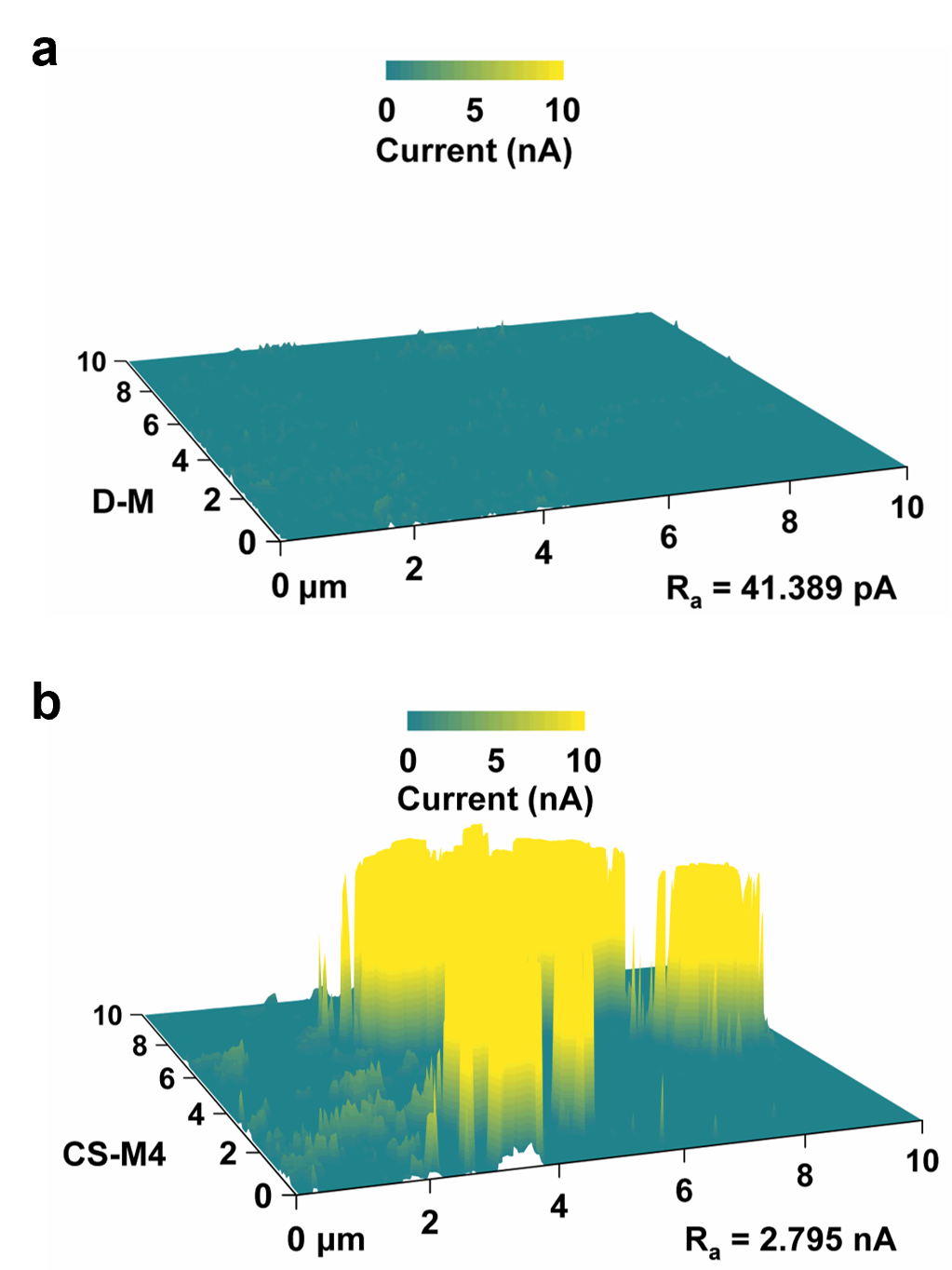


**Figure S16.** Three-dimensional conductive topographic images acquired by CAFM. (a) D-M, (b) CS-M4.


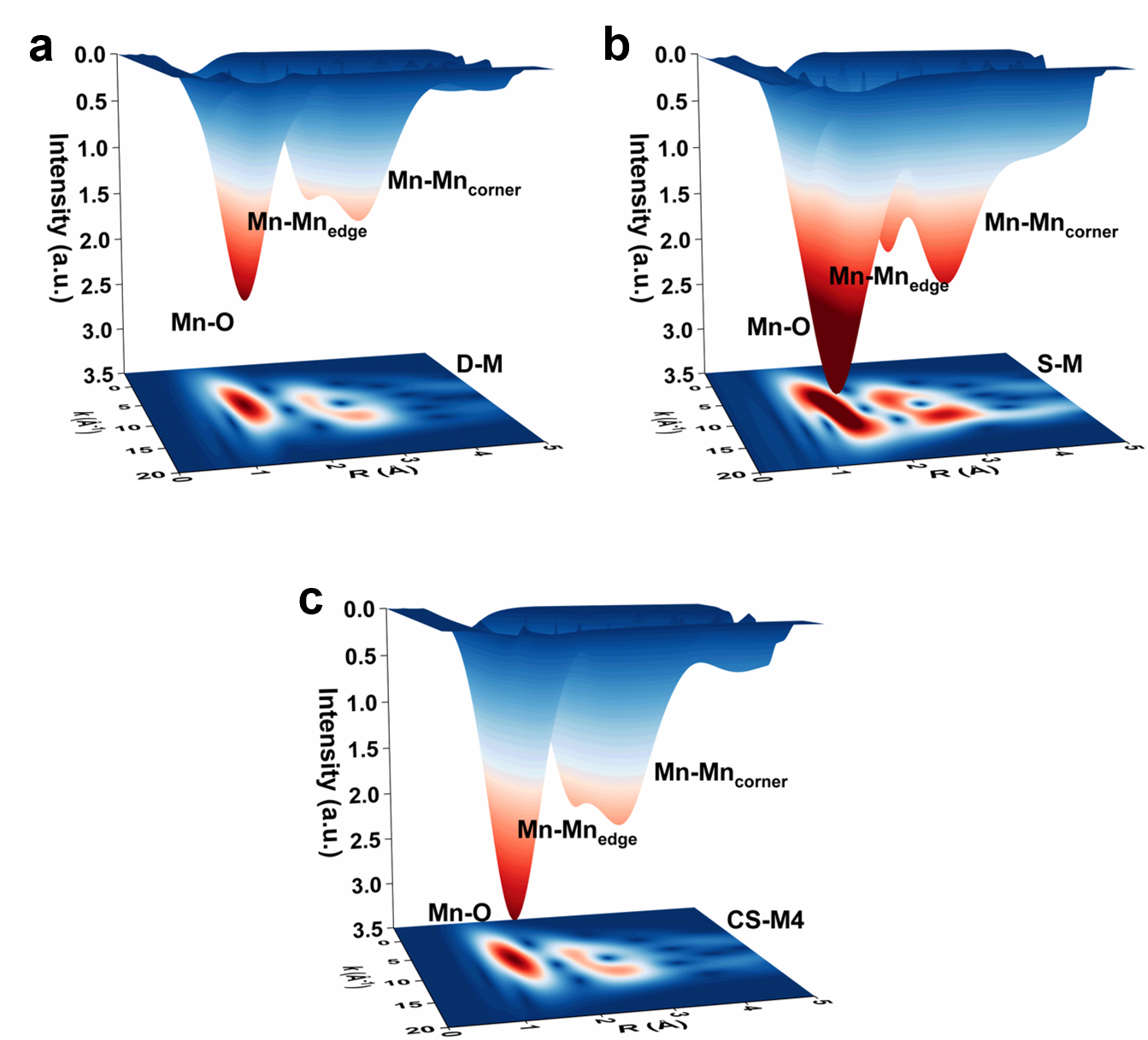


**Figure S17.** Wavelet transform analysis of the EXAFS signals for (a) D-M, (b) S-M and (c) CS-M4.


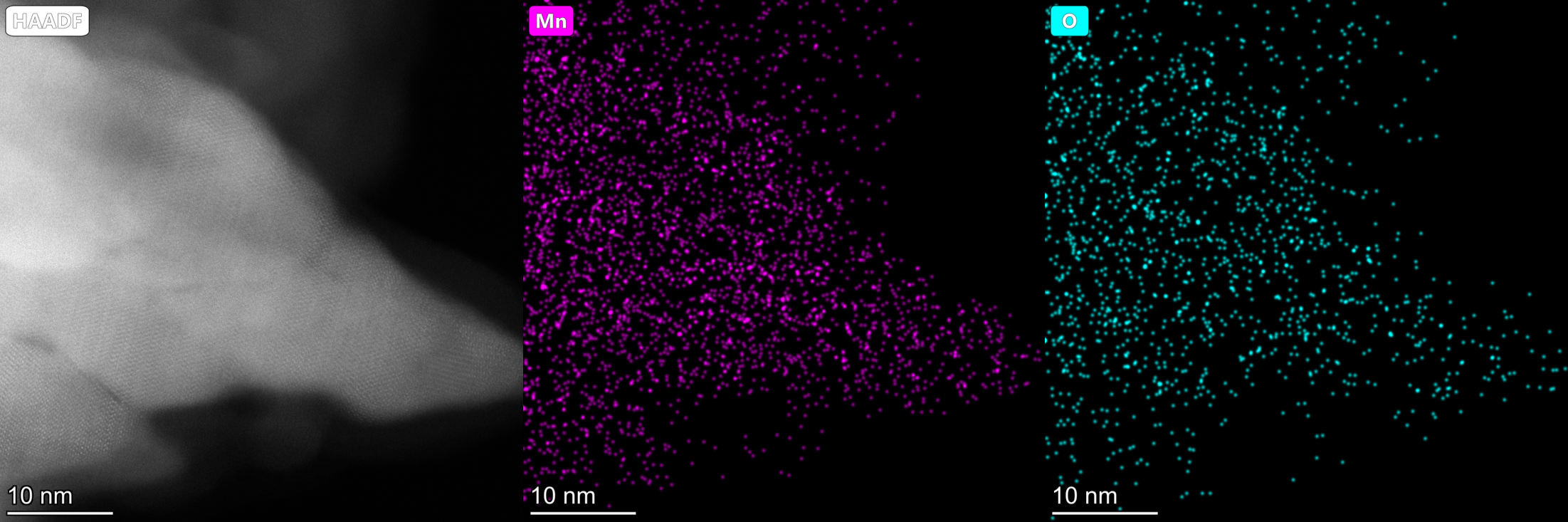


**Figure S18.** HAADF-STEM image and elemental mapping of CS-M4.


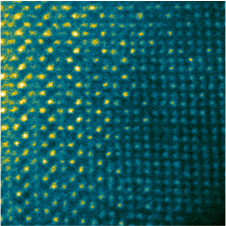


**Figure S19.** HAADF-STEM image of the γ/β phase transition region in CS-M4.


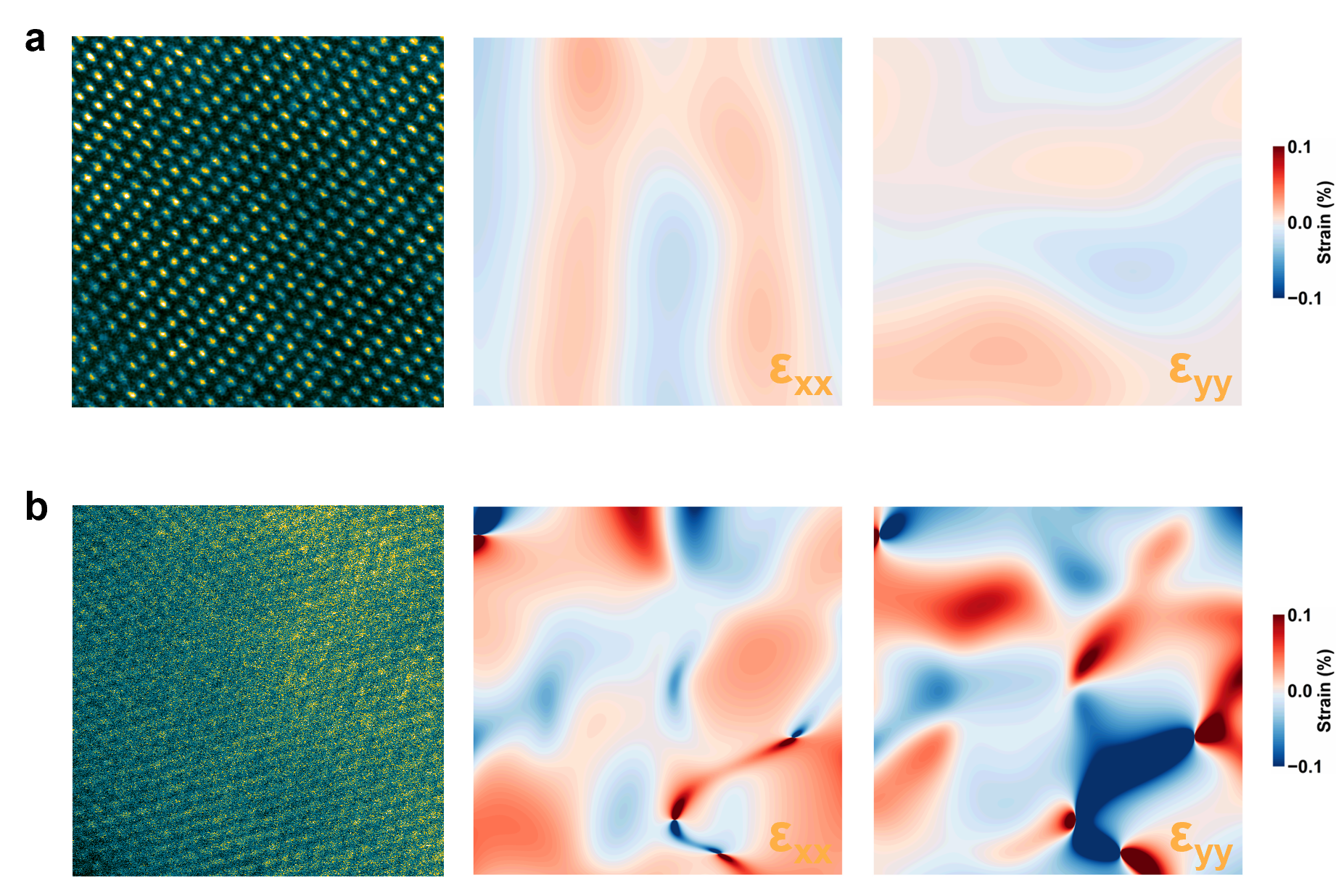


**Figure S20.** HAADF-STEM image of (a) S-M and (b) D-M, along with the corresponding GPA strain diagrams along the ε_xx_ and ε_yy_ directions.


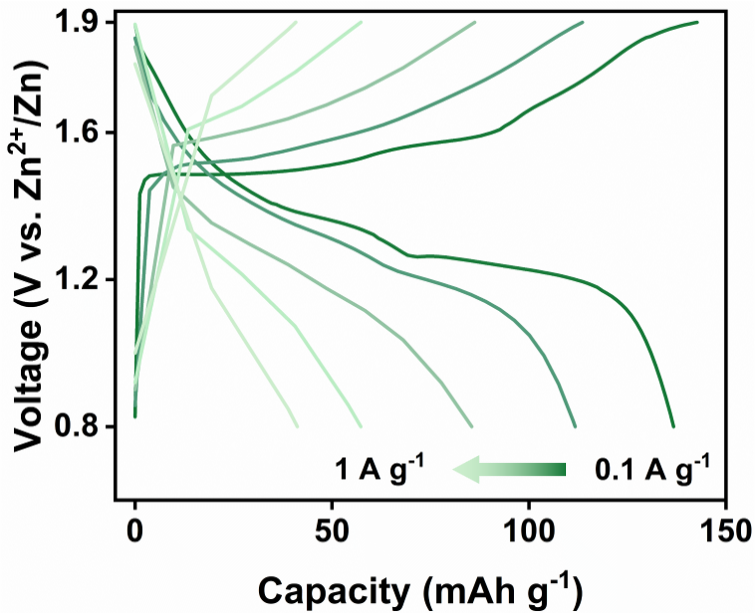


**Figure S21.** GCD profiles for D-M at various current densities corresponding to the rate capability test.


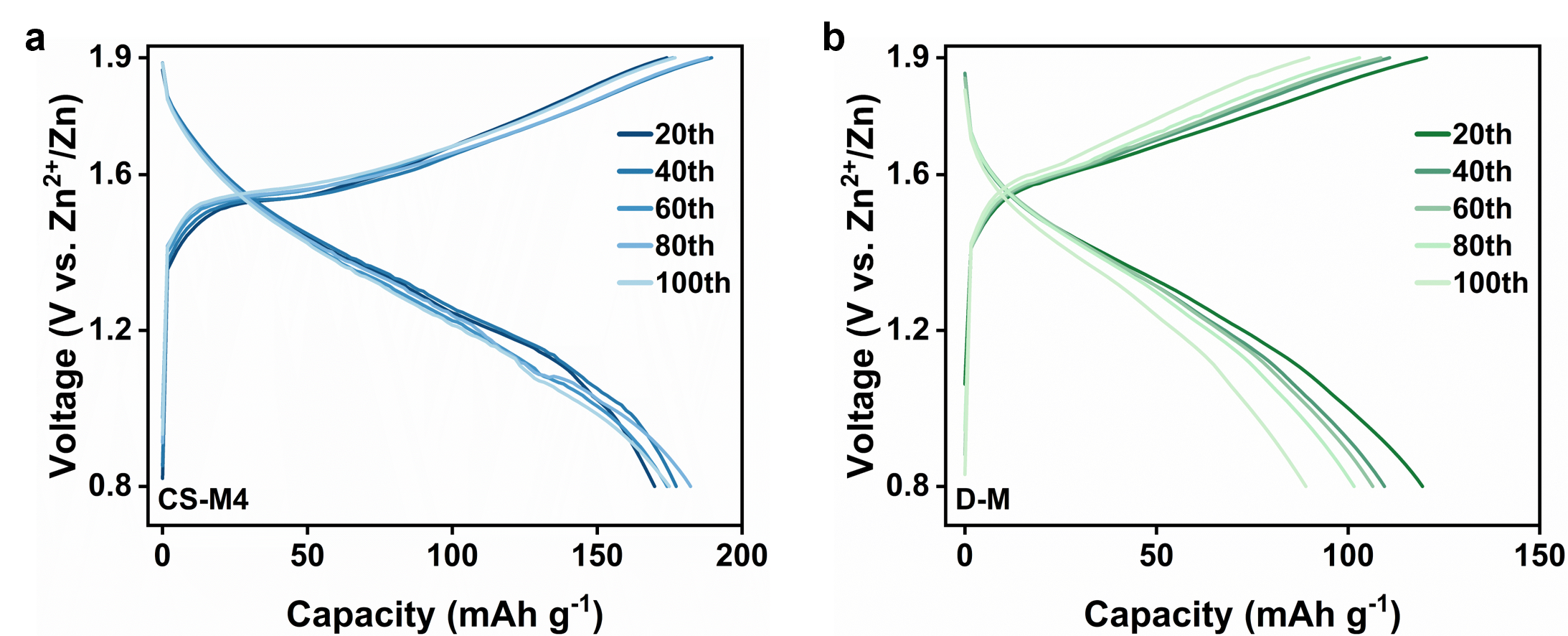


**Figure S22.** Comparison of GCD curves for (a) CS-M4 and (b) D-M at selected cycle intervals (20th, 40th, 60th, 80th, and 100th) during long-term cycling tests at a current density of 0.2 A g^−1^.


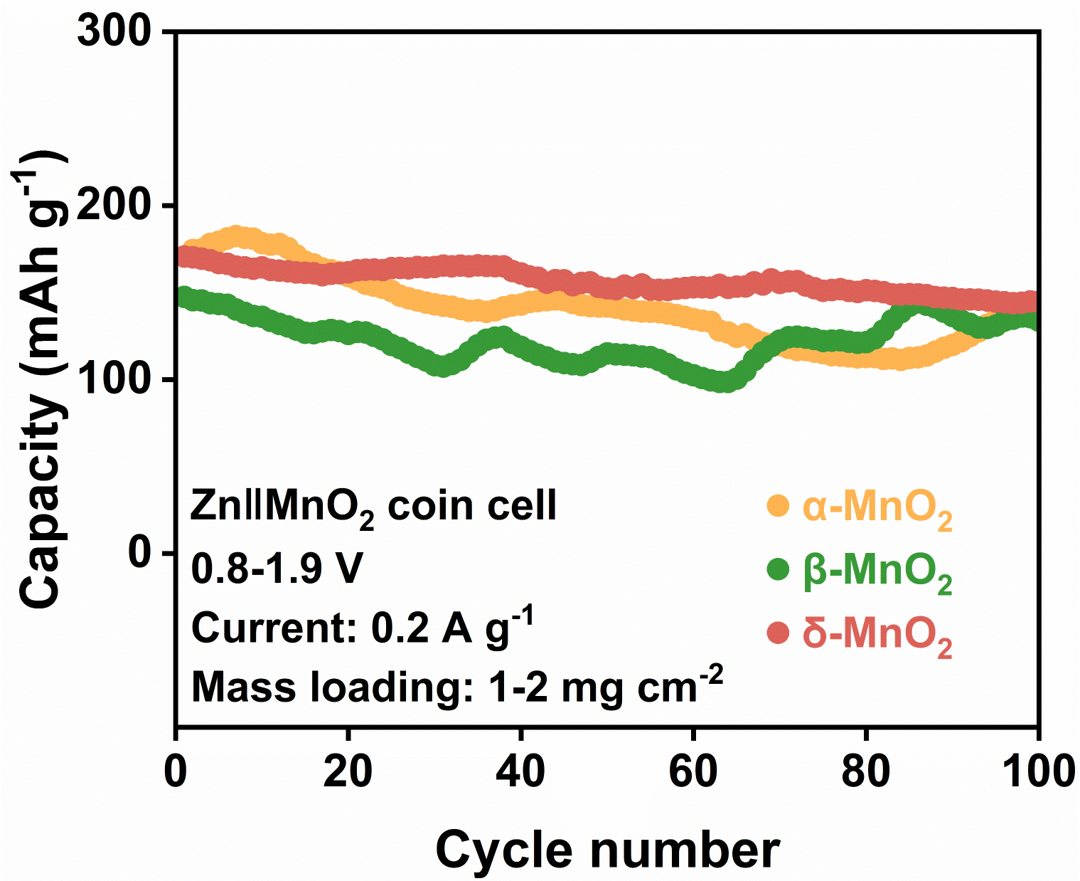


**Figure S23.** Comparative long-term cycling performance of hydrothermally synthesized α-, β- and δ-MnO_2_ at a current density of 0.2 A g^−1^.


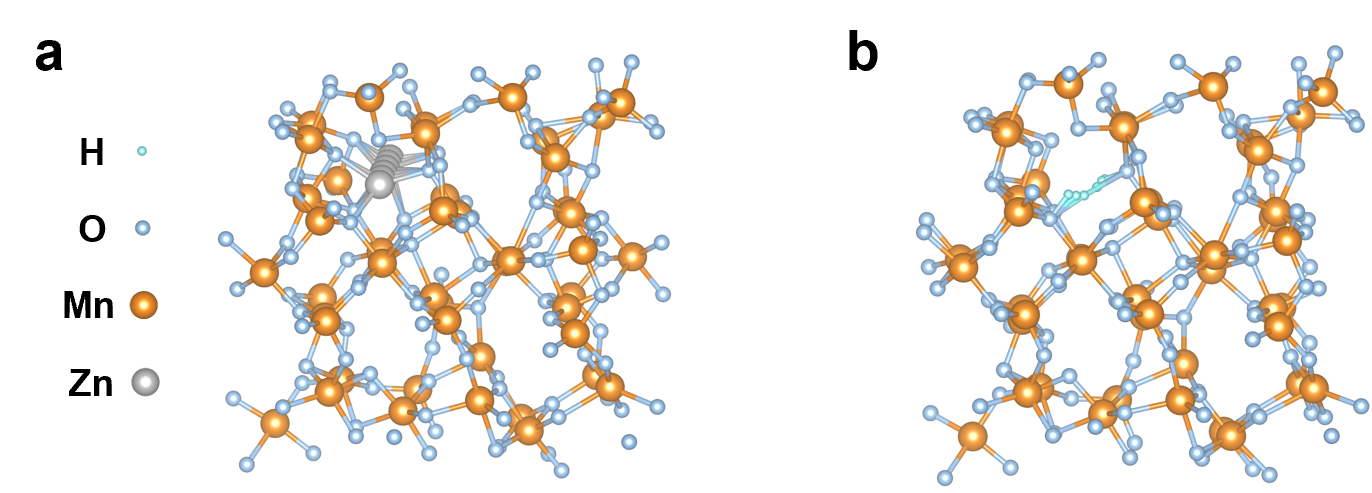


**Figure S24.** Schematic illustrations of (a) Zn^2+^ and (b) H^+^ migration pathways in D-M.


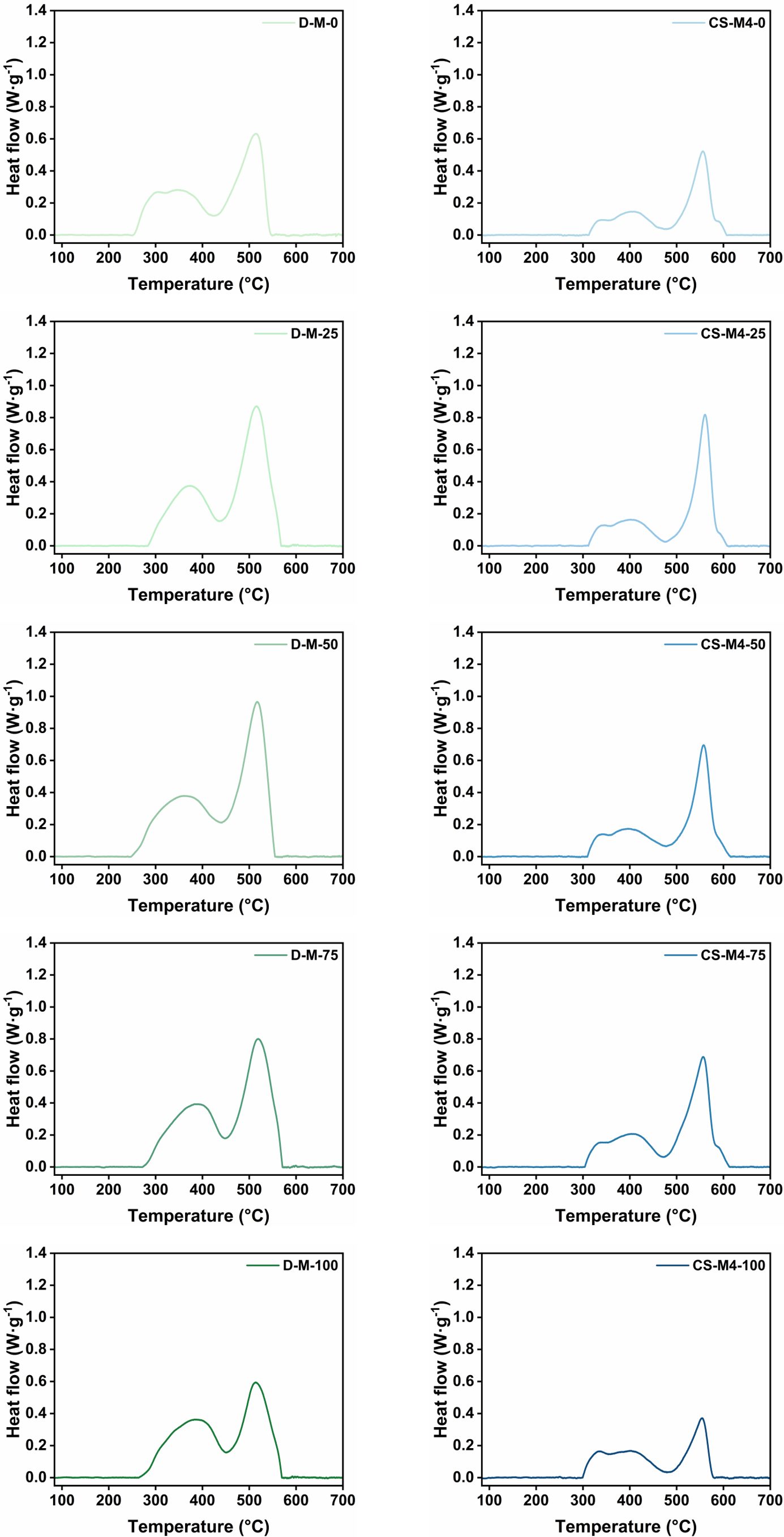


**Figure S25.** Heat-flow curves of the cycled D-M and CS-M4 pouch cells at various SOC.


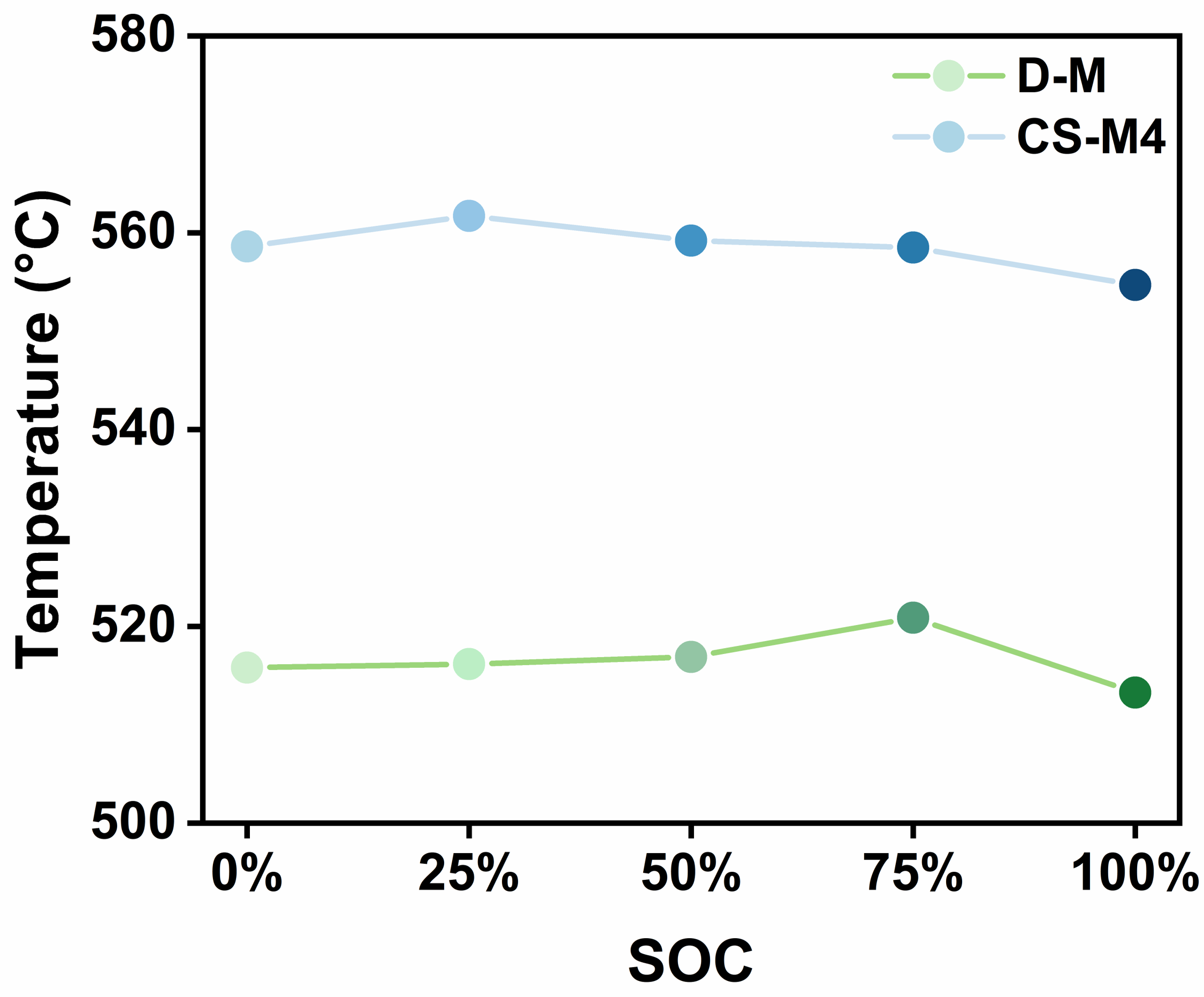


**Figure S26.** *T*_p_ of the DSC heat-flow curves for D-M and CS-M4 pouch cells at different SOCs.


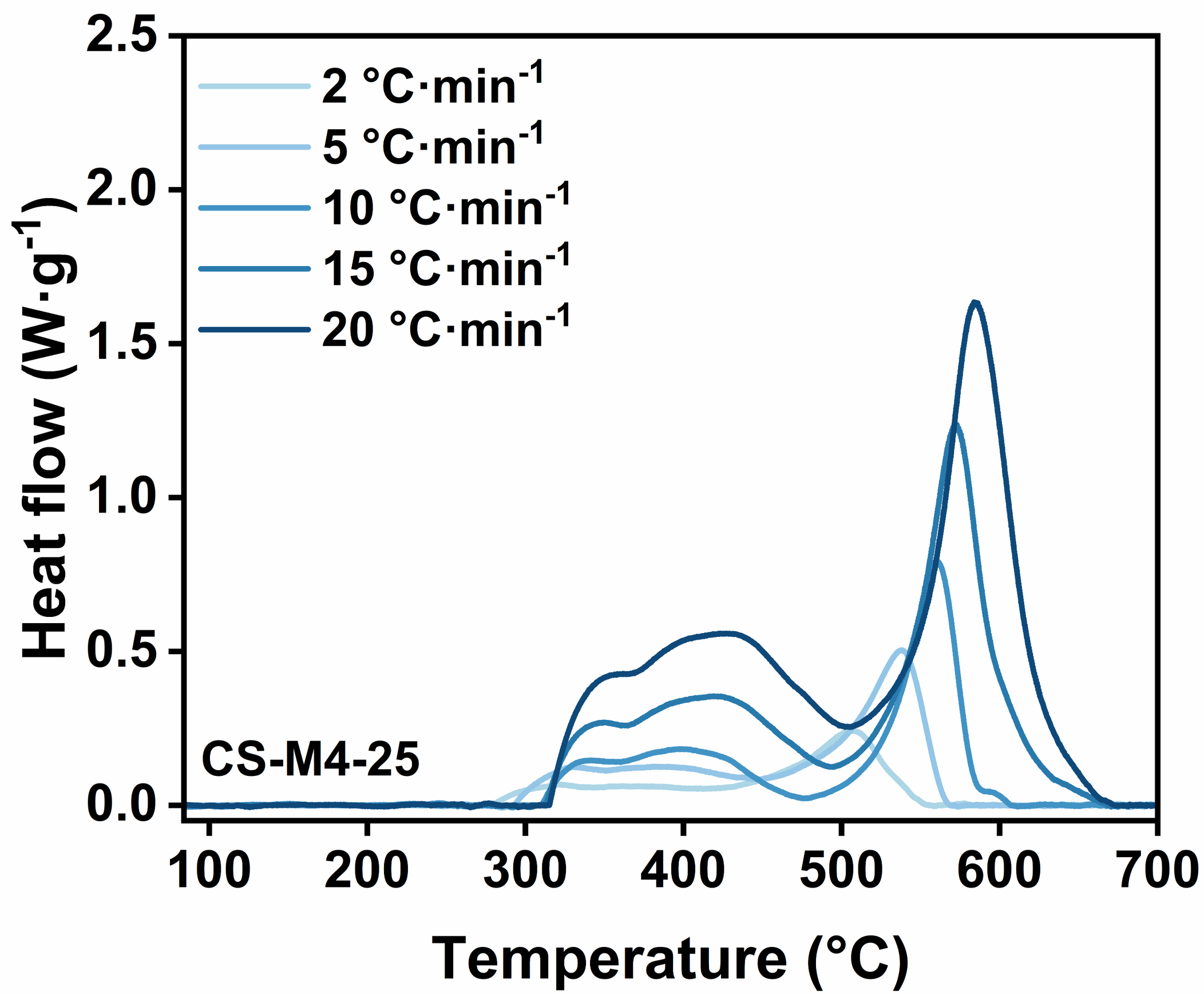


**Figure S27.** Heat-flow curves for CS-M4 at 25% SOC under different *β*.


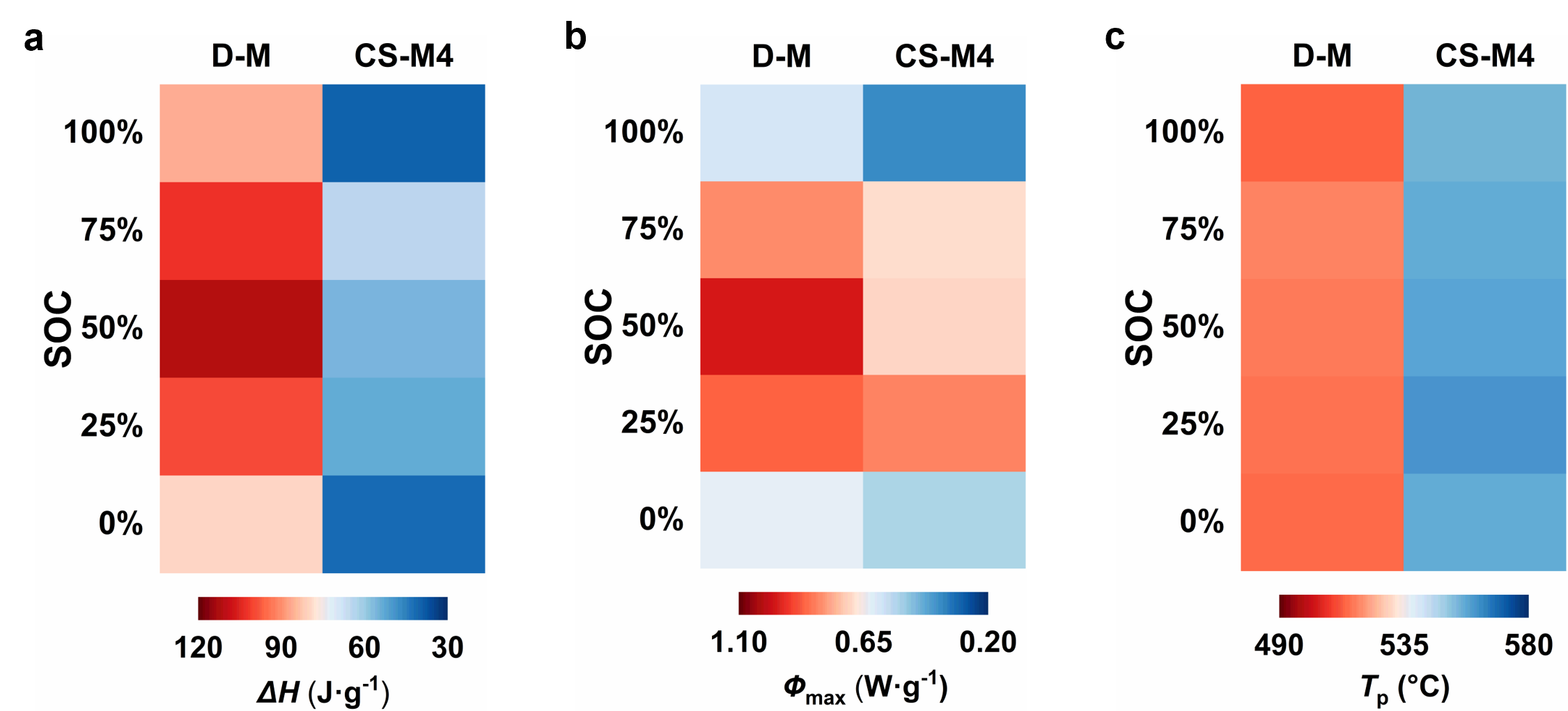


**Figure S28.** Comparative heatmaps of D-M and CS-M4 based on conventional thermal stability parameters: (a) Δ*H*, (b) *Ф*_max_, and (c) *T*_p_.


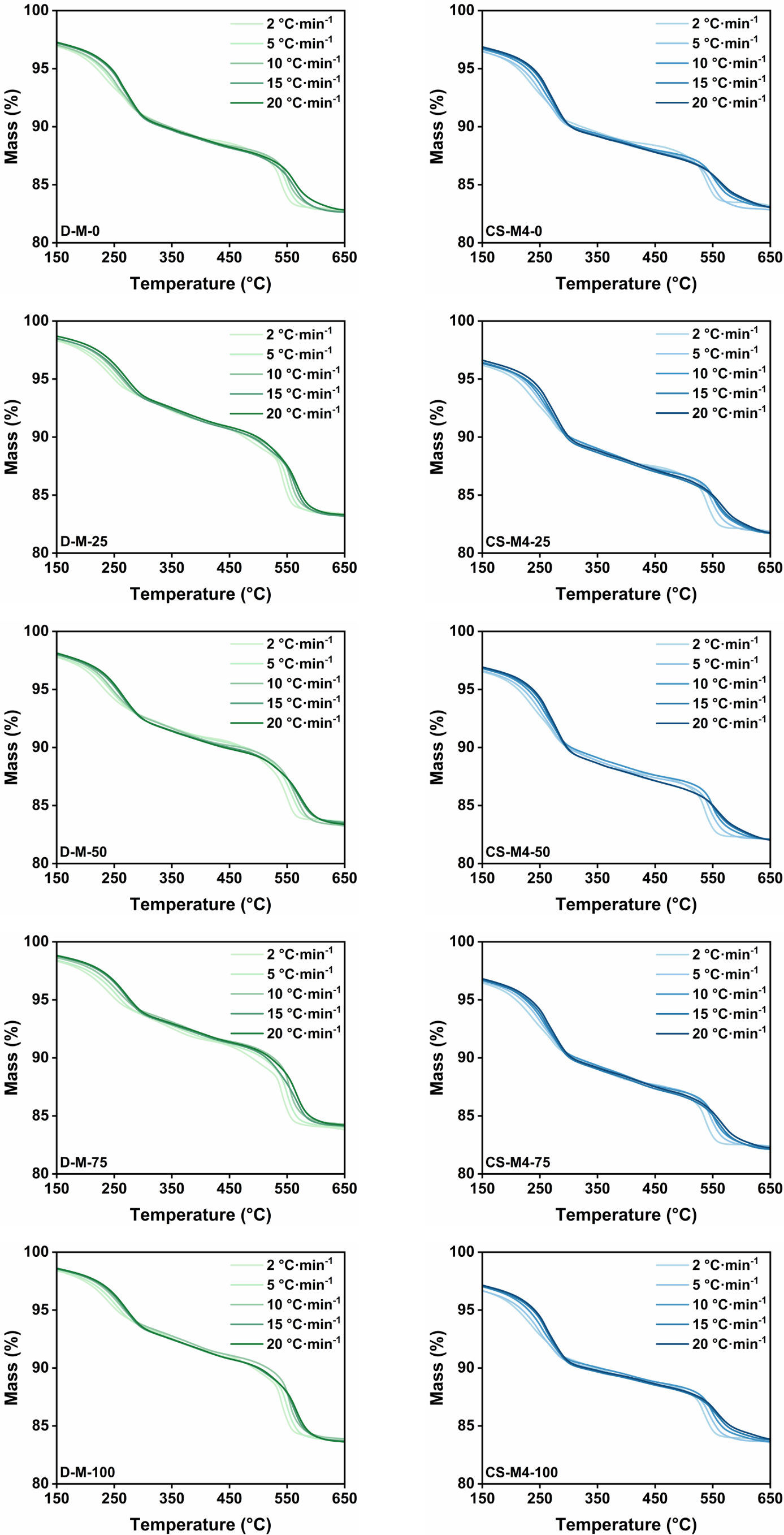


**Figure S29.** TG curves of the cycled D-M and CS-M4 pouch cells at various SOCs and under different *β*.


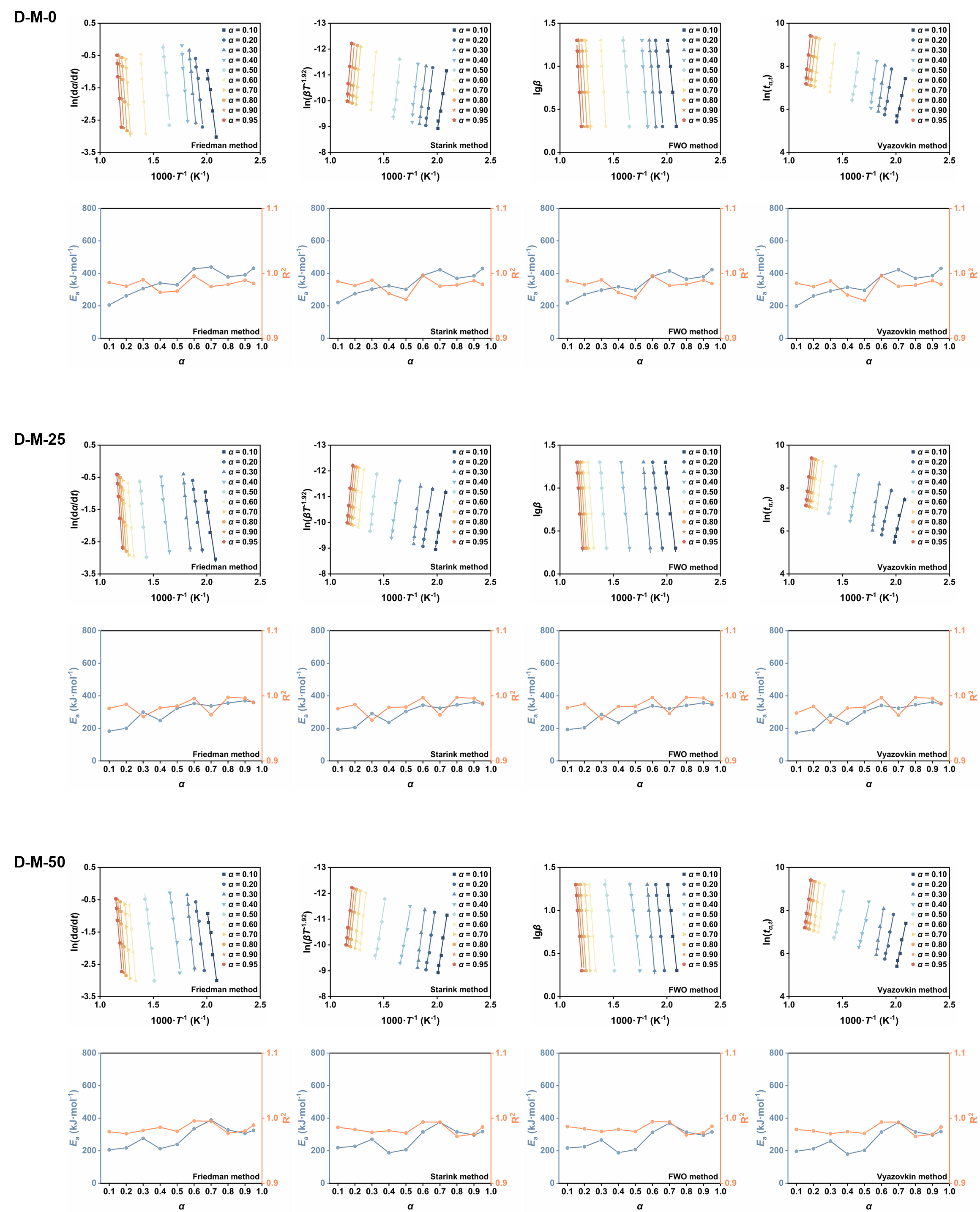


**Figure S30.** *E*_a_ calculated using four kinetic models (Friedman, Starink, FWO, and Vyazovkin) based on the TG data of D-M at different SOCs (0%, 25%, and 50%). The *E*_a_ and R^2^ values at various *α* for each model are also provided.


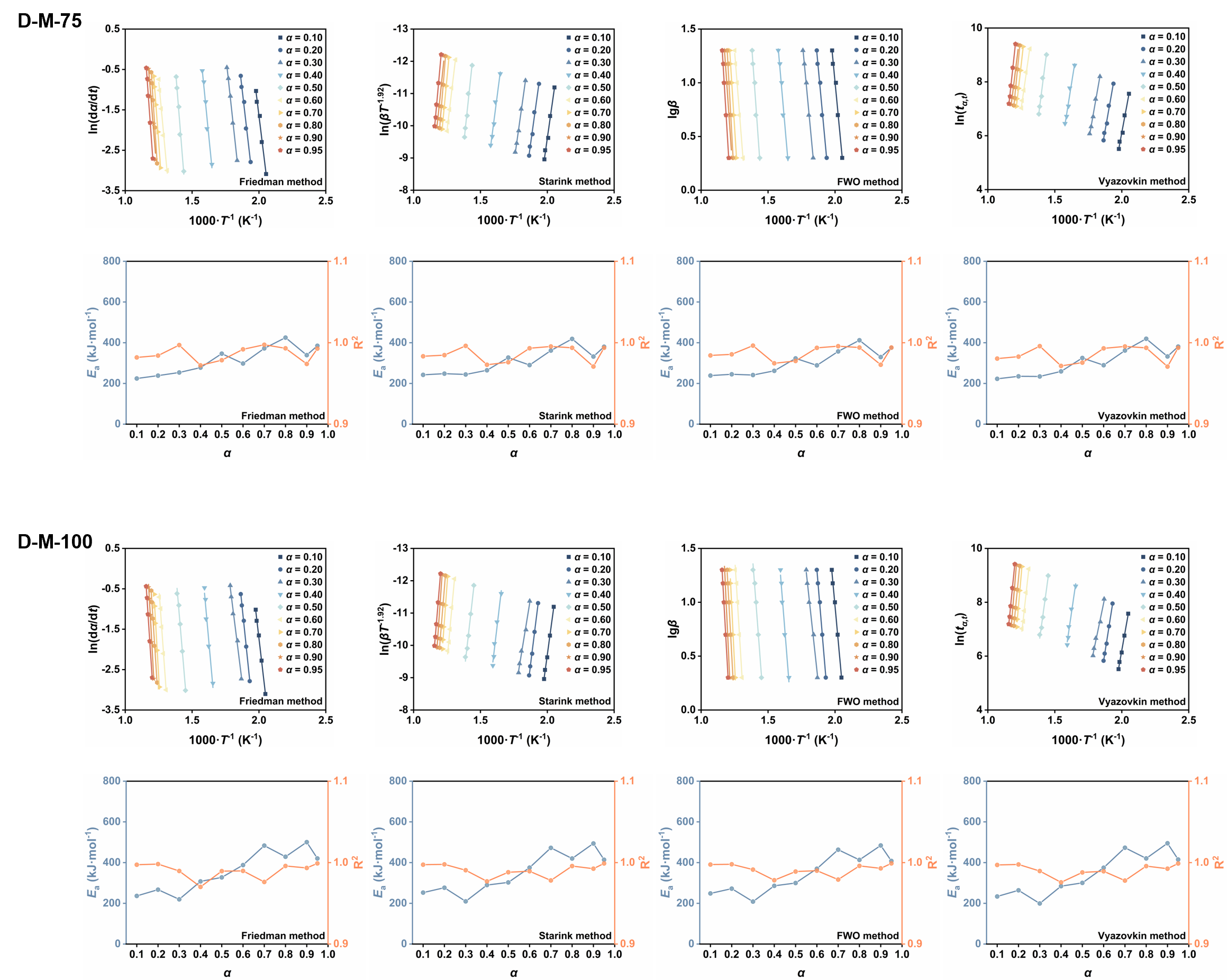


**Figure S31.** *E*_a_ calculated using four kinetic models (Friedman, Starink, FWO, and Vyazovkin) based on the TG data of D-M at different SOCs (75% and 100%). The *E*_a_ and R^2^ values at various *α* for each model are also provided.


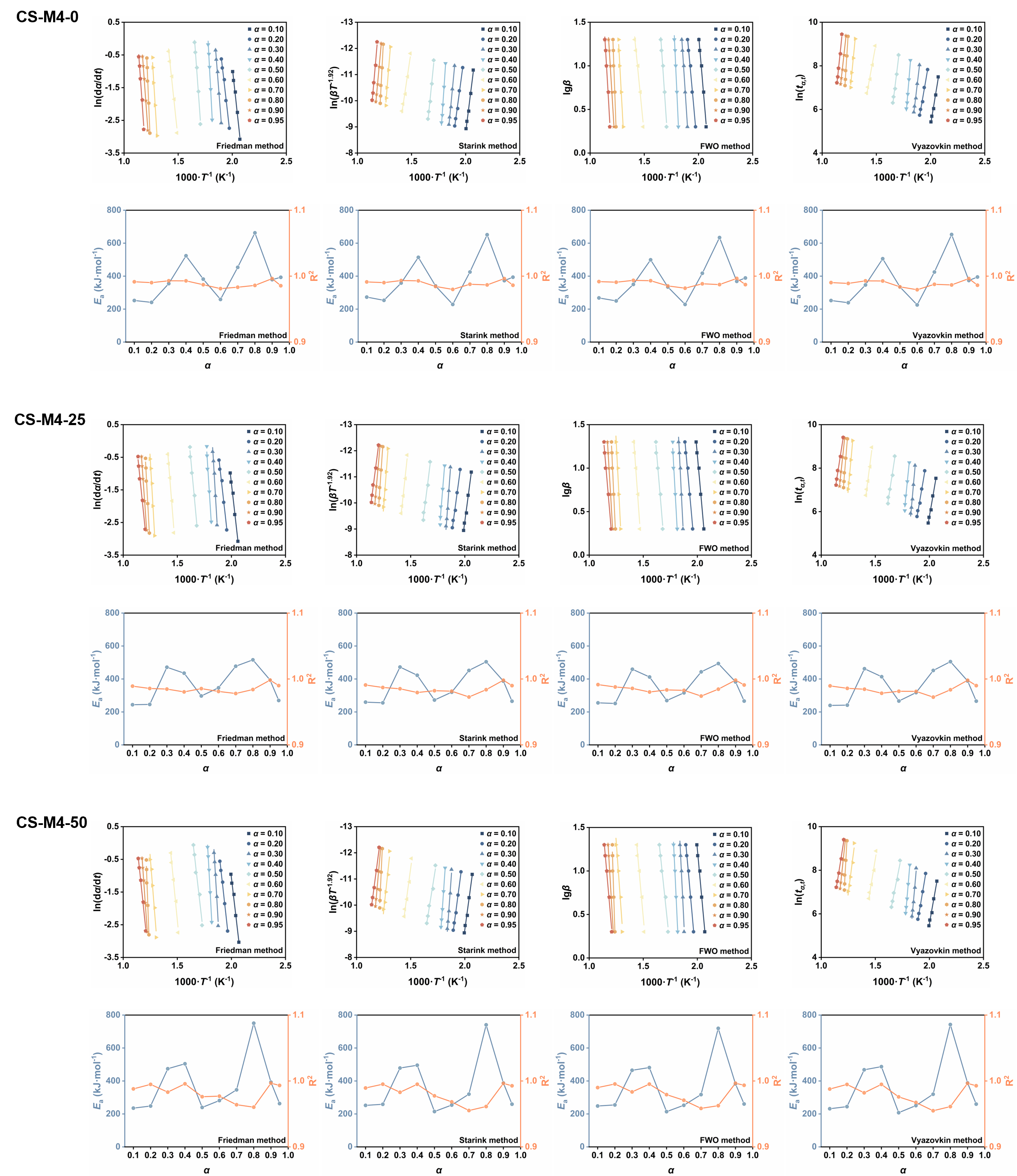


**Figure S32.** *E*_a_ calculated using four kinetic models (Friedman, Starink, FWO, and Vyazovkin) based on the TG data of CS-M4 at different SOCs (0%, 25%, and 50%). The *E*_a_ and R^2^ values at various *α* for each model are also provided.


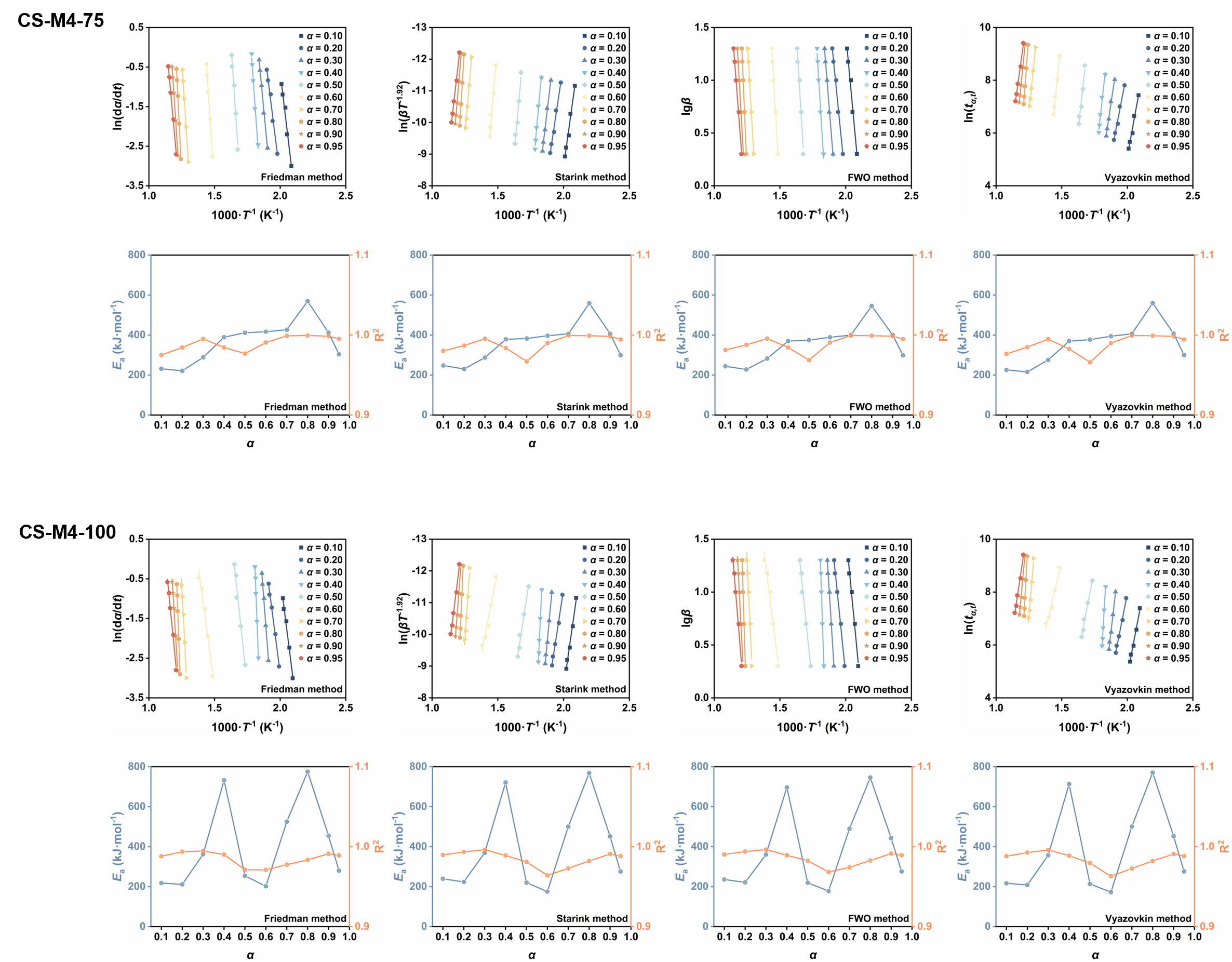


**Figure S33.** *E*_a_ calculated using four kinetic models (Friedman, Starink, FWO, and Vyazovkin) based on the TG data of CS-M4 at different SOCs (75% and 100%). The *E*_a_ and R^2^ values at various *α* for each model are also provided.


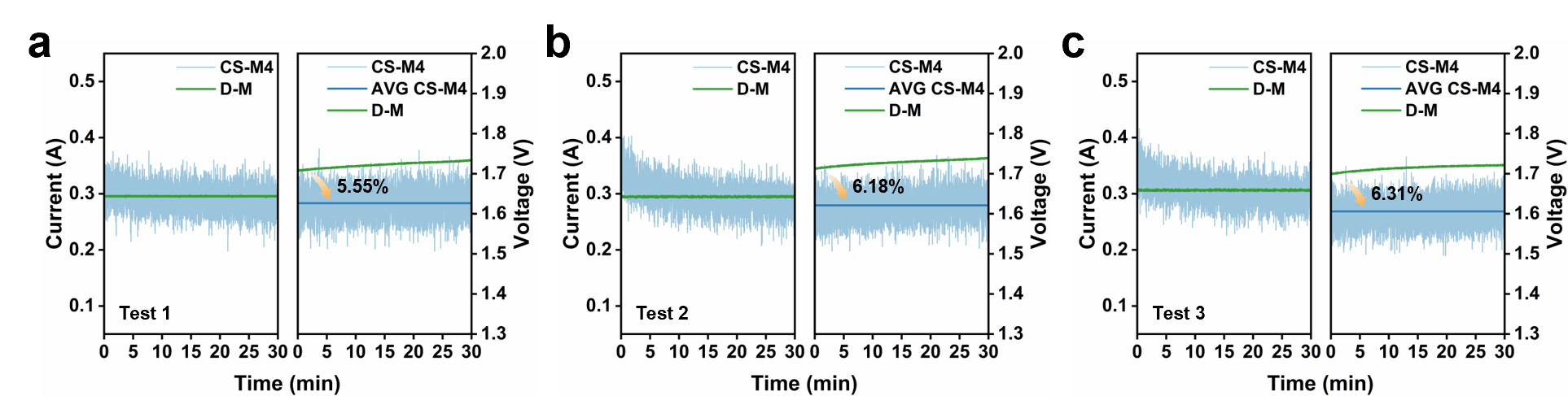


**Figure S34.** Three independent replicate tests of the energy-saving effect of the chaotic suspension electrolysis process.

First, a 30 min chaotic suspension electrolysis was conducted, controlling the average current at 0.3 A while collecting voltage-current signals. This was followed by a 30 min conventional direct-current electrolysis based on the measured average current, with signals collected accordingly. The energy-saving effect was determined by calculating the average voltage difference between the two processes. This test was repeated three times to ensure reliability of the results, and the final data represent the average of the three tests.


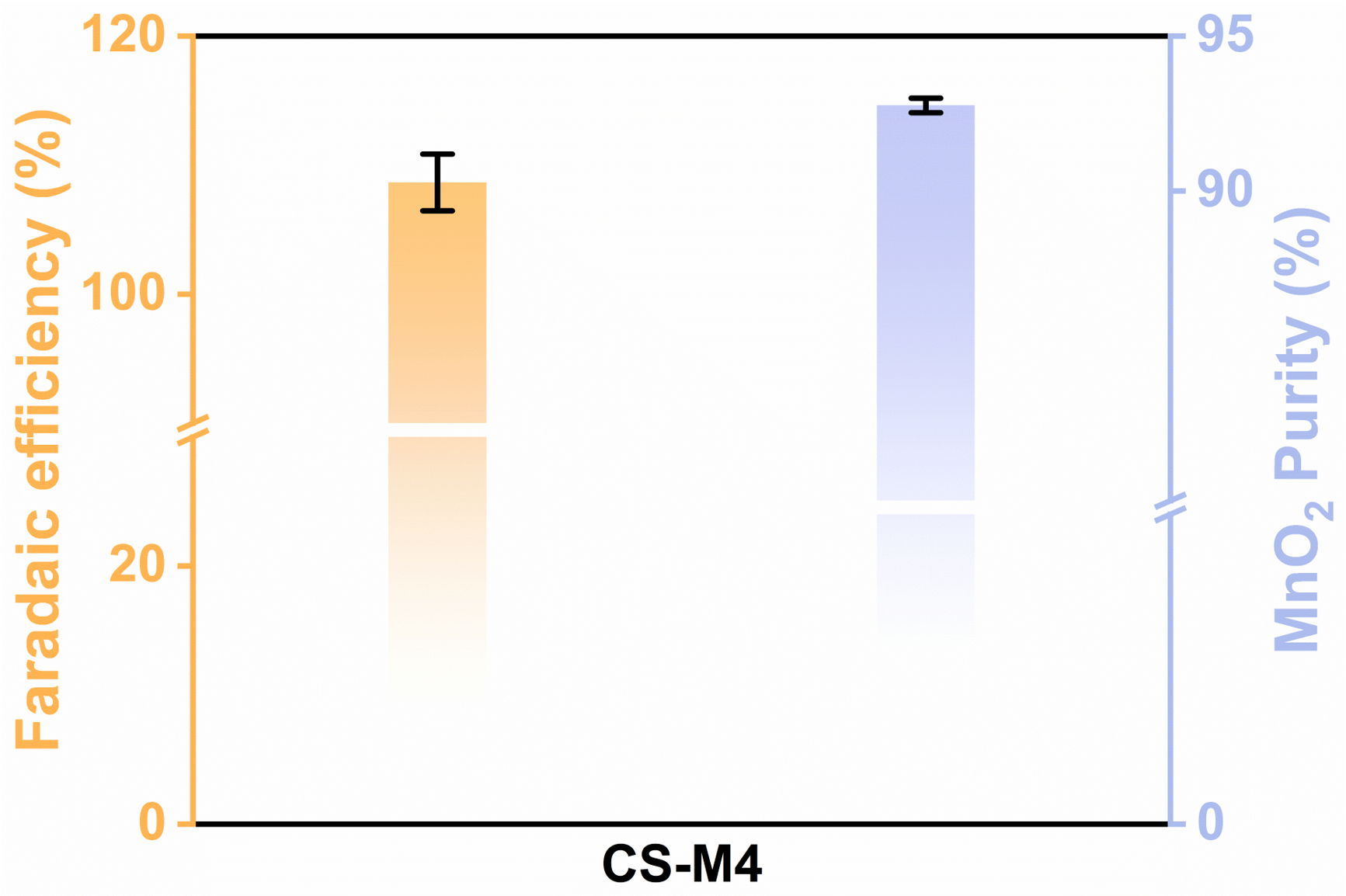


**Figure S35.** Analysis of Faradaic efficiency and MnO_2_ purity for CS-M4.


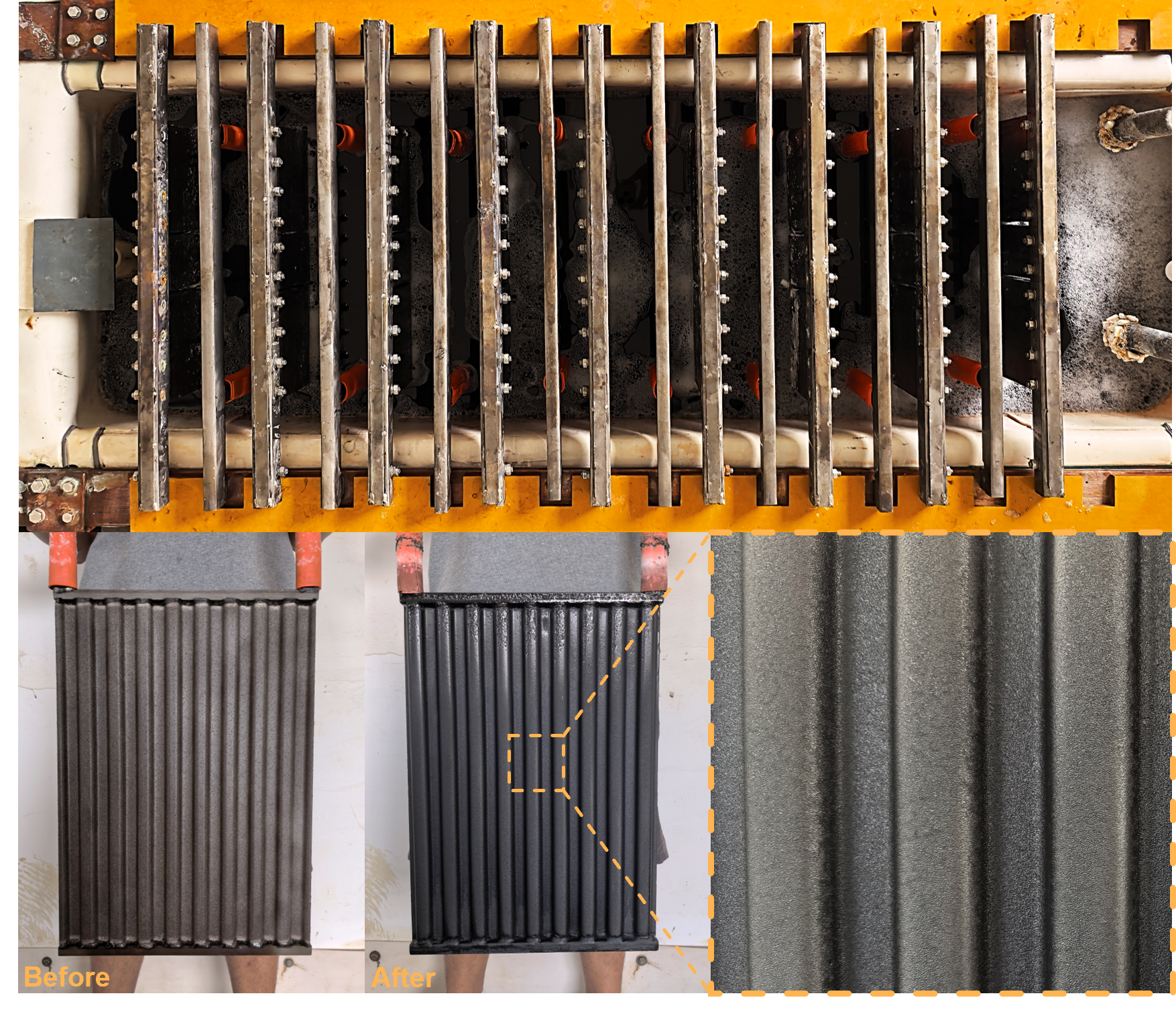


**Figure S36.** Photographs of the actual electrolysis site at industrial pilot scale.


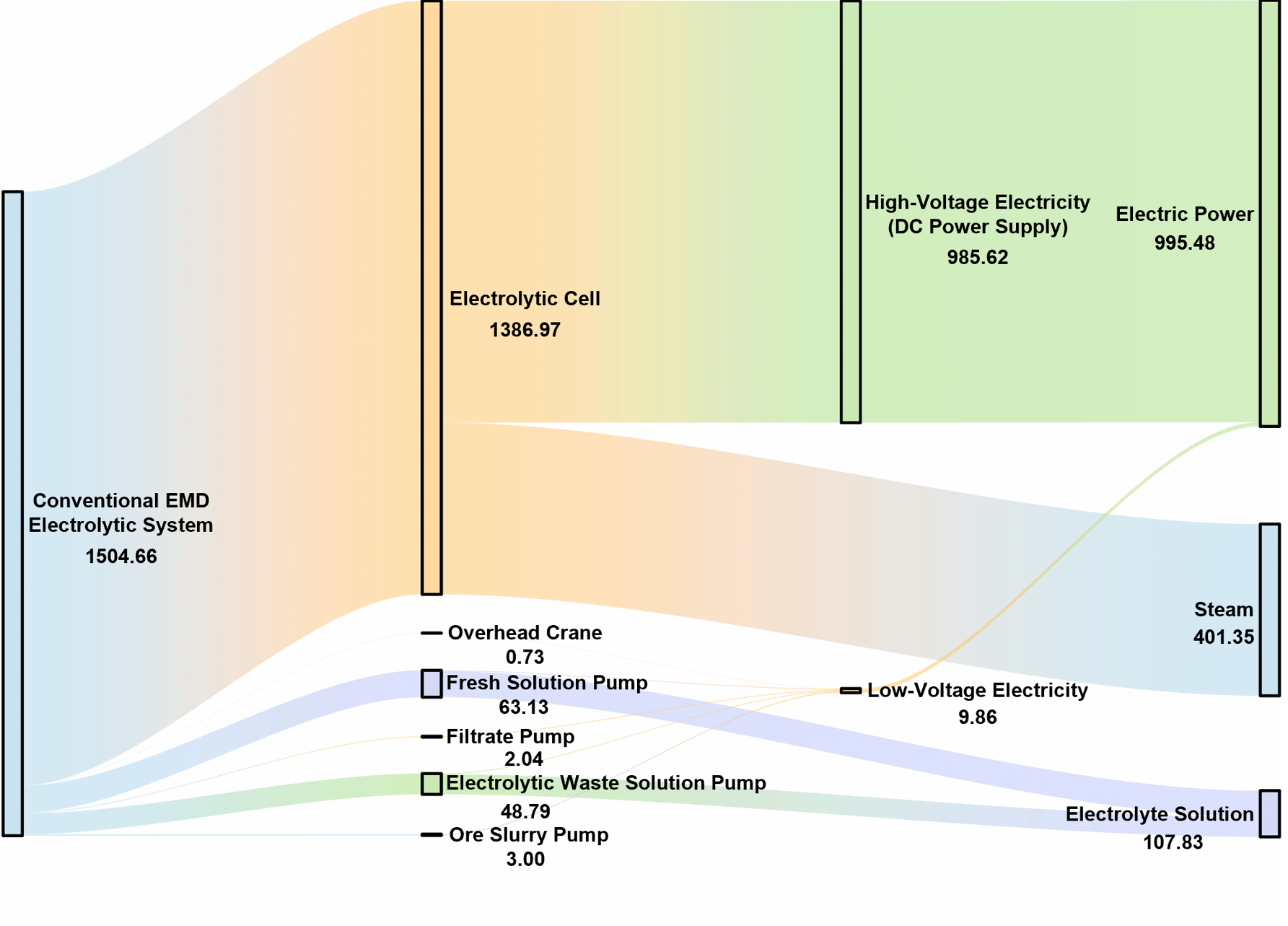


**Figure S37.** Sankey diagram of CO_2_ emission analysis for the conventional direct-current electrolysis process.


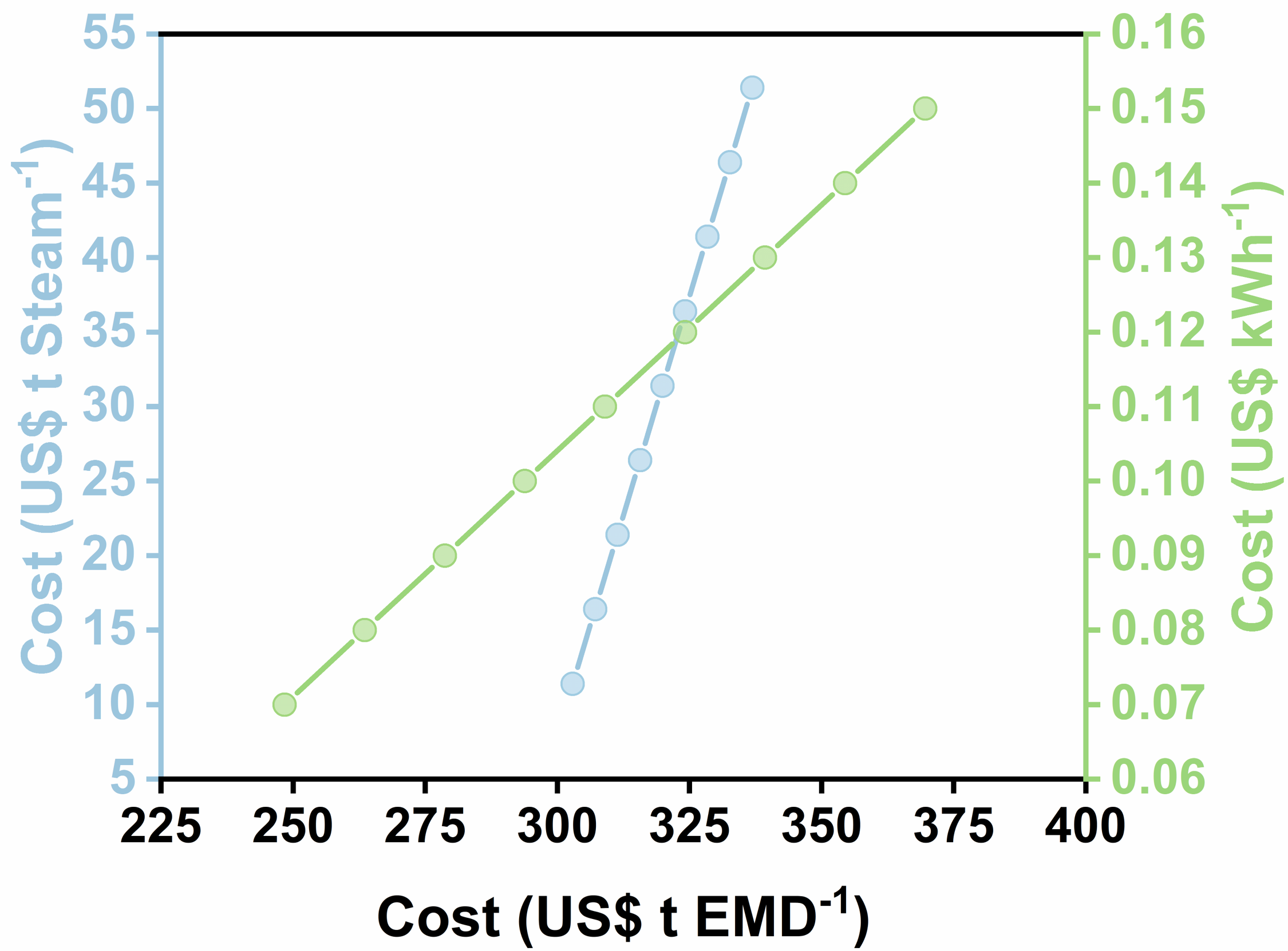


**Figure S38.** Sensitivity analysis of EMD production cost to electricity and steam prices.

**Table S1** Hydrothermal synthesis parameters for different MnO_2_ polymorphs.

| MnO_2_ Polymorph | Crystal Structure | Structure | Synthesis Method |
| --- | --- | --- | --- |
| α-MnO_2_ | 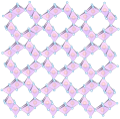 | [2 × 2] tunnel | A solution was prepared by dissolving 0.948 g of KMnO_4_ in 70 mL of deionized water, followed by the addition of 3 mL of H_2_SO_4_. The mixture was transferred into a 100 mL Teflon-lined stainless steel autoclave and reacted at 150 °C for 24 h. The product was collected by centrifugation, washed with deionized water until neutral, and dried at 60 °C for 12 h. |
| β-MnO_2_ | 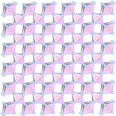 | [1 × 1] tunnel | A solution was prepared by dissolving 0.755 g of MnSO_4_ and 1.14 g of (NH_4_)_2_S_2_O_8_ in 40 mL of deionized water. The mixture was transferred into a Teflon-lined stainless steel autoclave and reacted at 140 °C for 12 h. The product was collected by vacuum filtration, sequentially washed with deionized water and ethanol, and dried at 60 °C for 12 h. |
| δ-MnO_2_ | 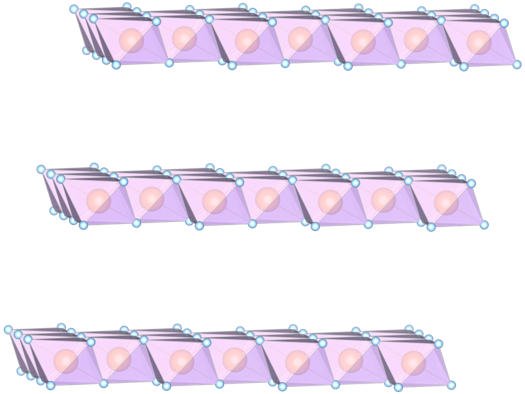 | Layer | A solution was prepared by dissolving 0.1264 g of KMnO_4_ and 0.0427 g of NH_4_Cl in 40 mL of deionized water. The mixture was transferred into a 50 mL Teflon-lined stainless steel autoclave and reacted at 140 °C for 24 h. The product was collected by vacuum filtration, washed with deionized water, and dried at 60 °C for 12 h. |

**Table S2** *E*_a_ (kJ mol^−1^) values for D-M and CS-M4 at various SOCs and their mean values, calculated using four thermodynamic models.

|  | D-M | | | | | CS-M4 | | | | |
| --- | --- | --- | --- | --- | --- | --- | --- | --- | --- | --- |
| SOC | Friedman | Starink | FWO | Vyazovkin | AVG | Friedman | Starink | FWO | Vyazovkin | AVG |
| 0 | 350.91 | 341.51 | 336.16 | 335.35 | 340.98 | 389.98 | 381.02 | 373.81 | 374.85 | 379.91 |
| 25 | 303.27 | 295.36 | 292.50 | 290.23 | 295.34 | 369.58 | 361.02 | 354.77 | 355.12 | 360.12 |
| 50 | 283.28 | 272.79 | 270.82 | 266.96 | 273.46 | 373.50 | 366.01 | 359.46 | 359.83 | 364.70 |
| 75 | 315.99 | 310.89 | 307.35 | 306.08 | 310.08 | 366.92 | 359.43 | 353.17 | 353.04 | 358.14 |
| 100 | 357.89 | 350.73 | 345.37 | 346.01 | 350.00 | 401.54 | 394.61 | 386.75 | 388.10 | 392.75 |

**Table S3** Energy consumption data for the milling system.

| Energy Type | Unit | Consumption | Percentage of Total |
| --- | --- | --- | --- |
| Electric Power | 10^4^ kW h | 703.80 | 100 |

**Table S4** Energy consumption data for the solution preparation system.

| Energy Type | Unit | Consumption | Percentage of Total |
| --- | --- | --- | --- |
| Electric Power | 10^4^ kW h | 597.33 | 14.1 |
| Steam | Ton | 44250 | 85.9 |

**Table S5** Energy consumption data for the electrolytic system.

| Energy Type | Unit | Consumption | Percentage of Total |
| --- | --- | --- | --- |
| Electric Power | 10^4^ kW h | 12168.24 | 63.17 |
| Steam | Ton | 86250 | 36.83 |

**Table S6** Energy consumption data for the post-treatment system.

| Energy Type | Unit | Consumption | Percentage of Total |
| --- | --- | --- | --- |
| Electric Power | 10^4^ kW h | 1109.78 | 6.29 |
| Steam | Ton | 201000 | 93.71 |

The electricity data presented in Tables S3–S6 were sourced from the local power grid, with an energy conversion standard coefficient of 0.1229 kgce kW^−1^ h^−1^. The steam was supplied by the industrial park's centralized heating system (with parameters of 0.3 MPa, 250 °C, corresponding to a specific enthalpy of 2963.47 kJ kg^−1^), and its conversion coefficient was taken as 0.1011 kgce kg^−1^. The percentage contributions of each energy type listed in the tables were calculated based on the uniform application of the aforementioned conversion coefficients.

**Table S7** Power load calculation for the electrolytic system.

| Equipment | Rated Power (kW) | Quantity | Total Capacity (kW) | Kx | cosφ | tgφ | Active Power (kW) | Reactive Power (kVar) | Apparent Power (kVA) |
| --- | --- | --- | --- | --- | --- | --- | --- | --- | --- |
| 380V Low-Voltage Power Distribution | | | | | | | | | |
| Overhead Crane | 10 | 6 | 60 | 0.2 | 0.5 | 1.73 | 12.00 | 20.76 | 24.00 |
| Fresh Solution Pump | 15 | 3 | 45 | 0.75 | 0.8 | 0.75 | 33.75 | 25.31 | 42.19 |
| Filtrate Pump | 15 | 3 | 45 | 0.75 | 0.8 | 0.75 | 33.75 | 25.31 | 42.19 |
| Electrolytic Waste Solution Pump | 15 | 3 | 45 | 0.75 | 0.8 | 0.75 | 33.75 | 25.31 | 42.19 |
| Ore Slurry Pump | 11 | 6 | 66 | 0.75 | 0.8 | 0.75 | 49.50 | 37.13 | 61.88 |
| Total | — | 24 | 294 |  |  |  | 184.20 | 149.91 |  |
| 35 kV High-Voltage Power Distribution | | | | | | | | | |
| Electrolytic Cell | 4095 | 6 | 24570 | 0.7 | 0.8 | 0.75 | 17199.00 | 12899.25 | 21498.75 |

**Table S8** Electricity consumption calculation for the electrolytic system.

| Equipment | Calculated Active Power, *P*_c_ (kW) | Annual Maximum Active Power Utilization Hours, *T*_max_ (h) | Annual Electricity Consumption, *W*_y_ (kW h) |
| --- | --- | --- | --- |
| Overhead Crane | 12.00 | 7000 | 84000 |
| Fresh Solution Pump | 33.75 | 7000 | 236250 |
| Filtrate Pump | 33.75 | 7000 | 236250 |
| Electrolytic Waste Solution Pump | 33.75 | 7000 | 236250 |
| Ore Slurry Pump | 49.50 | 7000 | 346500 |
| Electrolytic Cell | 17199.00 | 7000 | 120393000 |
| Total | — | — | 121532250 |

**Table S9** Material balance for the electrolytic system.

| Feed Name | Feed (t yr^−1^) | Product Name | Product (t yr^−1^) | Remarks |
| --- | --- | --- | --- | --- |
| Electrolyte | 142343.75 | EMD | 78418.89 |  |
| Steam | 86250.00 | Spent Electrolyte | 61600.00 | Recycled |
|  |  | Water Vapor | 22867.23 | Discharged |
|  |  | Steam Condensate | 65707.63 | Recycled |
| Total | 228593.75 | Total | 228593.75 |  |
